# Identification of essential genes for conjugative transfer in antimicrobial resistance-associated pELF-type linear plasmids of opportunistic pathogen *Enterococcus faecium*

**DOI:** 10.1371/journal.ppat.1013937

**Published:** 2026-08-12

**Authors:** Jun Kurushima, Natsuko Ota, Yuka Yoshii, Naoko Tomie, Haruyoshi Tomita

**Affiliations:** 1 Laboratory of Bacterial Drug Resistance, Gunma University Graduate School of Medicine, Showa-machi, Maebashi, Gunma, Japan; 2 Department of Bacteriology, Gunma University Graduate School of Medicine, Showa-machi, Maebashi, Gunma, Japan; University of Colorado Anschutz Medical Campus, UNITED STATES OF AMERICA

## Abstract

The pELF-type linear plasmid is a critical mobile genetic element responsible for the dissemination of various antimicrobial resistance (AMR) genes, most notably vancomycin resistance in *Enterococcus faecium*, which is a leading cause of hospital outbreaks worldwide. Despite their crucial role in the expansion of AMR, the molecular mechanisms underlying the conjugative transfer of these linear plasmids remain poorly understood. In this study, the transfer (*tra*) region of pELF2, a representative *vanA*-harboring linear plasmid was characterized. Transcriptomic data suggested that the FtsK/VirD4-type adenosine triphosphatase is encoded within a multi-gene operon. By developing a genetic manipulation framework for *E. faecium*, an extensive mutational analysis of the *tra* region was performed and the following three essential genes were identified: *traC*_B4_ (a putative VirB4 analog), *traD*_D4_ (a VirD4-like coupling protein), and *traG*_B6_ (a putative VirB6 analog). These genes are indispensable for conjugative transfer. Reporter assays experimentally confirmed the presence of a functional promoter upstream of the identified *tra* genes. We confirmed that these genes are highly conserved among pELF-type plasmid sequences deposited in public database. The study findings revealed that pELF-type plasmids utilize highly minimized conjugation machinery, which is similar to unusual systems previously identified in other gram-positive bacteria, such as *Streptomyces*. This study provides the first molecular insights into the transmission of these clinically important linear plasmids in enterococci and lays a foundation for understanding the dissemination of resistance determinants mediated by atypical mobile genetic elements.

## Author summary

Owing to its ability to acquire multidrug resistance via horizontal gene transfer, *Enterococcus faecium* is a major opportunistic pathogen responsible for severe healthcare-associated infections. Although most enterococcal resistance plasmids are circular, recently discovered linear pELF-type plasmids have emerged globally as critical vehicles for disseminating high-level resistance, such as vancomycin resistance. Despite their clinical importance, the mechanism by which these unusual linear DNA molecules move between bacterial cells remains unclear. In this study, the several essential genes that drive the conjugative transfer of pELF-type plasmids were identified. The study findings reveal that these plasmids utilize a minimized set of transfer machinery, though the potential involvement of other uncharacterized genes warrants further investigation. By analyzing more than 9,000 *E. faecium* genomes, the study further demonstrated that this specific transfer machinery is highly conserved and serves as a hallmark of this plasmid family. This study provides the first molecular blueprint for the transmission mechanism of pELF-type plasmids, thereby offering crucial insights into the role of these unique mobile elements in the global spread of antibiotic resistance in hospital environments.

## Introduction

*Enterococcus faecium* is a gram-positive coccoid bacterial species that commonly inhabits the gut microbiota of healthy humans and is widely distributed across natural environments [1]. However, *E. faecium* is an opportunistic pathogen, and vancomycin (VAN)-resistant *E. faecium* has emerged as one of the most concerning antimicrobial-resistant (AMR) bacterial pathogens [2]. Among the related bacteria, *Enterococcus* species harbor large plasmids that drive rapid genetic evolution by enabling the acquisition of new genes, such as those conferring antimicrobial resistance [3]. In *E. faecium*, horizontal gene transfer of antimicrobial resistance genes (ARGs) is mediated by conjugative circular plasmids with various *rep* types, including pMG1-like plasmid (e.g., pMG1, pHTβ, and pZB18) or pRepA_N-group plasmid (e.g., pRUM-type plasmids) [4–21].

*Enterococcus faecium* is divided into clades A1, A2, and B [22]. Clade A1 is hospital-associated with a high risk of enterococcal infection and contains the clonal complex 17 (CC17) lineage. This lineage is recognized as the highest risk lineage for human health according to conventional multilocus sequence typing [23]. Clade A2 is phylogenetically close to clade A1; however, the clade is primarily associated with animal hosts rather than human infections. Clade B *E. faecium* is a major symbiont bacterial species in the human gut and was re-designated as the species *Enterococcus lactis*; hence, the clade is genetically distinct from Clade A [24]. Notably, despite these differences in clinical risk, all *E. faecium* clades and *E. lactis* share conjugative plasmid types that harbor ARGs [25,26]. These findings emphasize that horizontal transfer via conjugative plasmids plays a key role in the dissemination of ARGs among *E. faecium* and *E. lactis* strains in various ecological sectors, including humans, animals, and environmental resources [27].

In general, the conjugative transfer of circular plasmids is a highly complex process mediated by a multi-component machinery known as the Type IV secretion system (T4SS), which forms a specialized translocation channel spanning the cell envelope [28,29]. This system typically requires more than ten different proteins, including a core complex that bridges the inner and outer membranes, and multiple adenosine triphosphatases (ATPase), such as VirB4, VirB11, and VirD4, which coordinately provide energy for apparatus assembly and substrate translocation. In contrast, linear plasmids exhibit a broad functional diversity across various taxa [30]. For instance, the prophage-like linear plasmid pBSSB1 is found in Enterobacterales (e.g., *Salmonella* and *Klebsiella*) [31], whereas lp25 and lp28–1 in *Borrelia* are essential for virulence and genome maintenance; however, they generally lack autonomous conjugative ability [32]. Among the mobile linear plasmids, those from actinomycetes are the most extensively characterized. In *Streptomyces*, the conjugation of linear plasmids, such as SLP2 and pSLA1, is primarily driven by an FtsK-like ATPase motor protein, which facilitates the transfer of double-stranded DNA [33–35]. In striking contrast to the structural complexity of the circular T4SS, the linear plasmid SAP1 from *Streptomyces* is an extremely minimalist transfer system with only two factors (the translocator TraA and essential protein TraB), which are sufficient for successful conjugation [35].

The pELF-type plasmid was recently discovered in E. faecium and is a large (>100 kb) linear DNA molecule that serves as a platform for the integration of multiple genetic elements [36]. The pELF1 plasmid was the first reported pELF-type plasmid isolated from a hospitalized patient in Japan and harbored both *vanA*- and *vanM*-type VAN resistance gene clusters [7]. Linear pELF-type plasmids have frequently been isolated in association with multiple ARGs, including those conferring glycopeptide and linezolid resistance [37–44]. In addition to antimicrobial resistance, the pELF-type plasmid harbors metabolic gene sets that confer a competitive survival advantage in the human intestine [45]. The pELF-type plasmids are highly conjugative among *Enterococcus* species and stably maintained under laboratory conditions; however, *E. faecium* and *E. lactis* appear to be their natural hosts [36]. Notably, pELF-type linear plasmids lack the conventional conjugation T4SS gene sets characterized by circular conjugative plasmids [46] or known linear conjugative plasmids from other bacterial species [34,47]. In this study, the conjugative transfer mechanisms of the pELF-type linear conjugative plasmid family were analyzed and genes essential for conjugative transfer of the pELF plasmid were identified.

## Results

### Transcriptomic analysis defined the boundaries of a putative conjugation operon in the pELF-type linear plasmid

**Fig 1:**
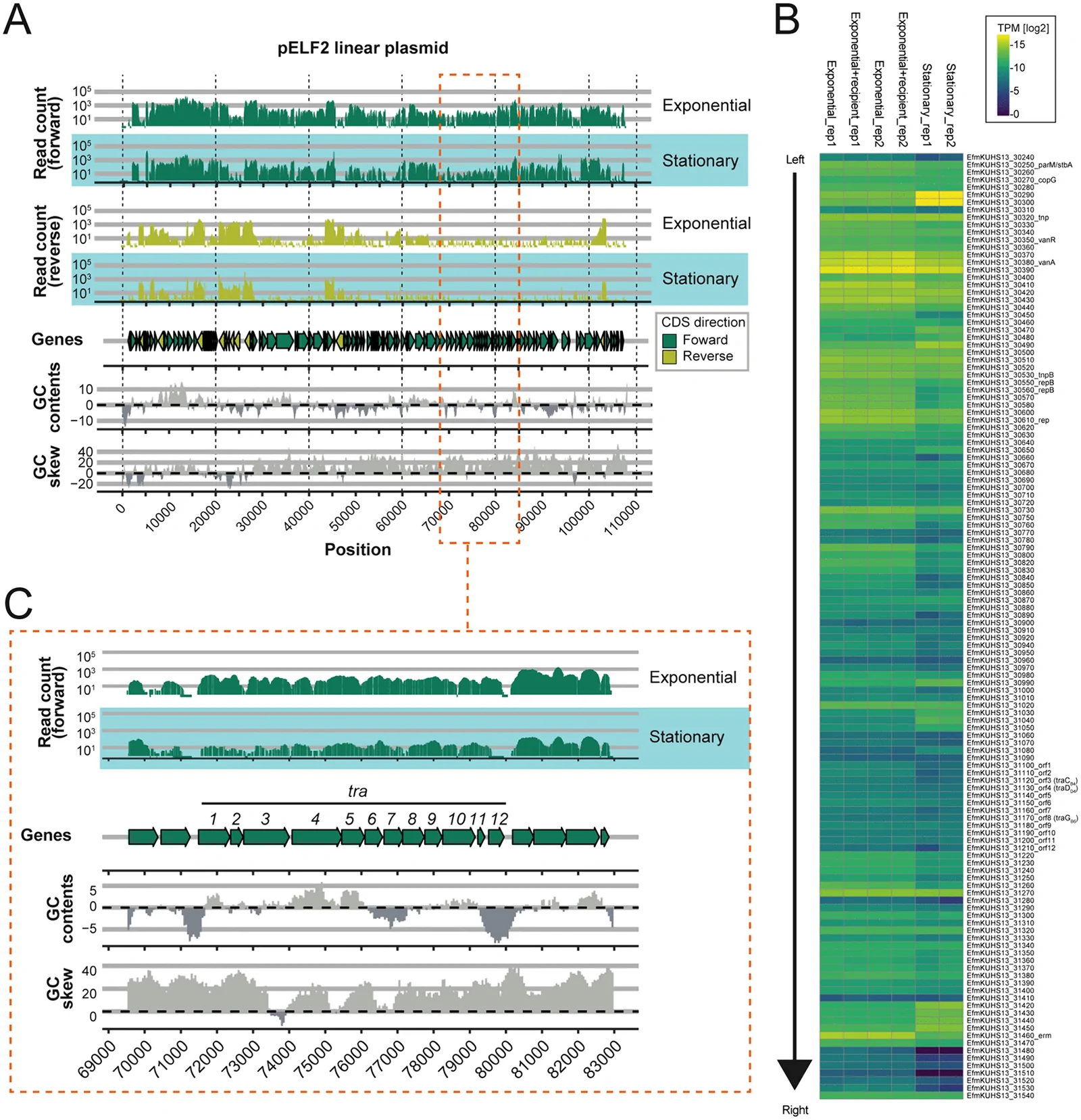
Transcriptional profile of the pELF2 linear plasmid. (A) Schematic presentation of the RNA-seq coverage plot on pELF2 plasmid. Mapping data for the forward or reverse strand, as well as annotated coding sequences (CDSs; accession no. AP022343) located on the forward or reverse strands, are indicated in green and yellow, respectively. Bottom panels display GC contents and GC skew across the pELF2 nucleotide sequence. (B) Gene expression matrix for strain KUHS13 during the exponential growth phase (with or without recipient cell BM4105RF) and the stationary growth phase. Expression levels are shown as TPM (transcripts per million), a normalized unit that accounts for both gene length and sequencing depth. (C) Enlarged view of panel A, focusing on the *tra* genes region to provide greater detail of its features.

Strain KUHS13 is a clade A1-lineage *E. faecium* isolate that harbors the pELF2 plasmid (AP022343.1), which is a pELF-type linear plasmid carrying Tn*1546* and encodes the *vanA*-type VAN resistance operon [48]. To analyze the transcriptional profile of genes encoded on the pELF2 plasmid, total RNA was isolated from KUHS13 during the exponential (4 h) or stationary (8 h) phases of culture in BHI broth, with or without co-culture with the plasmid-null recipient strain BM4105RF, a laboratory model E. faecium strain exhibiting rifampicin and fusidic acid resistance (S1 Fig) [16]. Growth phase-dependent transcriptional changes were observed among several plasmid-encoded genes (S2 Fig, S1 and S2 Tables), whereas co-culture with recipient cells had limited effect on the transcriptional profile (Fig 1A and 1B).

EfmKUHS13_31130 in pELF2 encodes a homolog of the FtsK-type ATPase, also known as type IV coupling protein (T4CP) [49]. RNA sequencing (RNA-seq) read coverage suggested that EfmKUHS13_31130 was transcribed as part of an operon-like structure containing 12 putative coding sequences (CDS), designated *orf1–12* (Table 1). This putative conjugation (*tra*) operon was designated as a candidate genetic element involved in the conjugative transfer of pELF-type plasmids (Fig 1C). In contrast to the T4CP homolog (*orf4*), the remaining CDSs showed no similarity to the conventional plasmid conjugation genes. Transmembrane domains were detected in the gene products of *orf3*, *orf8*, *orf10*, and *orf12*. The *orf1* gene product contains a nonspecific endonuclease domain. Although *orf3* does not exhibit an overall similarity to known proteins, it possesses a predicted ATPase domain at its C-terminus. The *orf7* encodes an unknown protein with a DNA-binding domain similar to that of Tc3 transposase. The *orf8* gene product contains a rhomboid-family intramembrane serine protease domain and multiple transmembrane domains. The *orf11* encodes a small protein with a DNA-binding motif. The remaining gene products (*orf2*, *orf5*, *orf6*, *orf9*, *orf10* and *orf12*) lacked the known functional domains.

**Table 1:** Genes encoded in *tra* operon.

Gene	Locus tag	Start^1^	End^1^	length (nt)	length (a.a.)	Essentiality on conjugation^2^	TM domain^3^	Predicted functional domain^4^	Predicted interaction with^5^
*orf1*	EfmKUHS13_31100	71’490	72’359	870	289	NT	None	DNA/RNA nonspecific endonuclease	
*orf2*	EfmKUHS13_31110	72’379	72’726	348	115	NT	None		
*orf3_traC*	EfmKUHS13_31120	72’739	74’001	1’263	420	Essential	2	ATPase (partial)	TraD
*orf4_traD*	EfmKUHS13_31130	74’081	75’457	1’377	458	Essential	None	FtsK-type ATPase	TraC
*orf5*	EfmKUHS13_31140	75’454	76’083	630	209	Non essential	None		
*orf6*	EfmKUHS13_31150	76’102	76’581	480	159	Non essential	None		
*orf7*	EfmKUHS13_31160^6^	76’623	77’162	540	179	Non essential	None	DNA-binding domain of Tc3 transposase	
*orf8_traG*	EfmKUHS13_31170	77’140	77’739	600	199	Essential	6	GlpG-like intramembrane rhomboid family serine protease	
*orf9*	EfmKUHS13_31180	77’755	78’231	477	158	Non essential	None		
*orf10*	EfmKUHS13_31190	78’248	79’150	903	300	Non essential	2		
*orf11*	EfmKUHS13_31200	79’223	79’417	195	64	NT	None	DNA-binding protein	
*orf12*	EfmKUHS13_31210	79’522	79’950	429	142	Non essential	1		

^1^Positions are indicated from nucleotide sequence of pELF2 plasmid (NZ_AP022343.1).

^2^Roles in conjugation refer to the results in Fig 2.

^3^Transmembrane (TM) domains were detected with TMHMM v2.0 (https://services.healthtech.dtu.dk/services/TMHMM-2.0/).

^4^Functional domains were predicted with HHPred (https://toolkit.tuebingen.mpg.de/tools/hhpred).

^5^Protein-protein interactions were predicted with alphafold 3 (https://alphafoldserver.com).

^6^Open reading frame was re-annotated in this study.

NT, Not tested.

**Fig 2:**
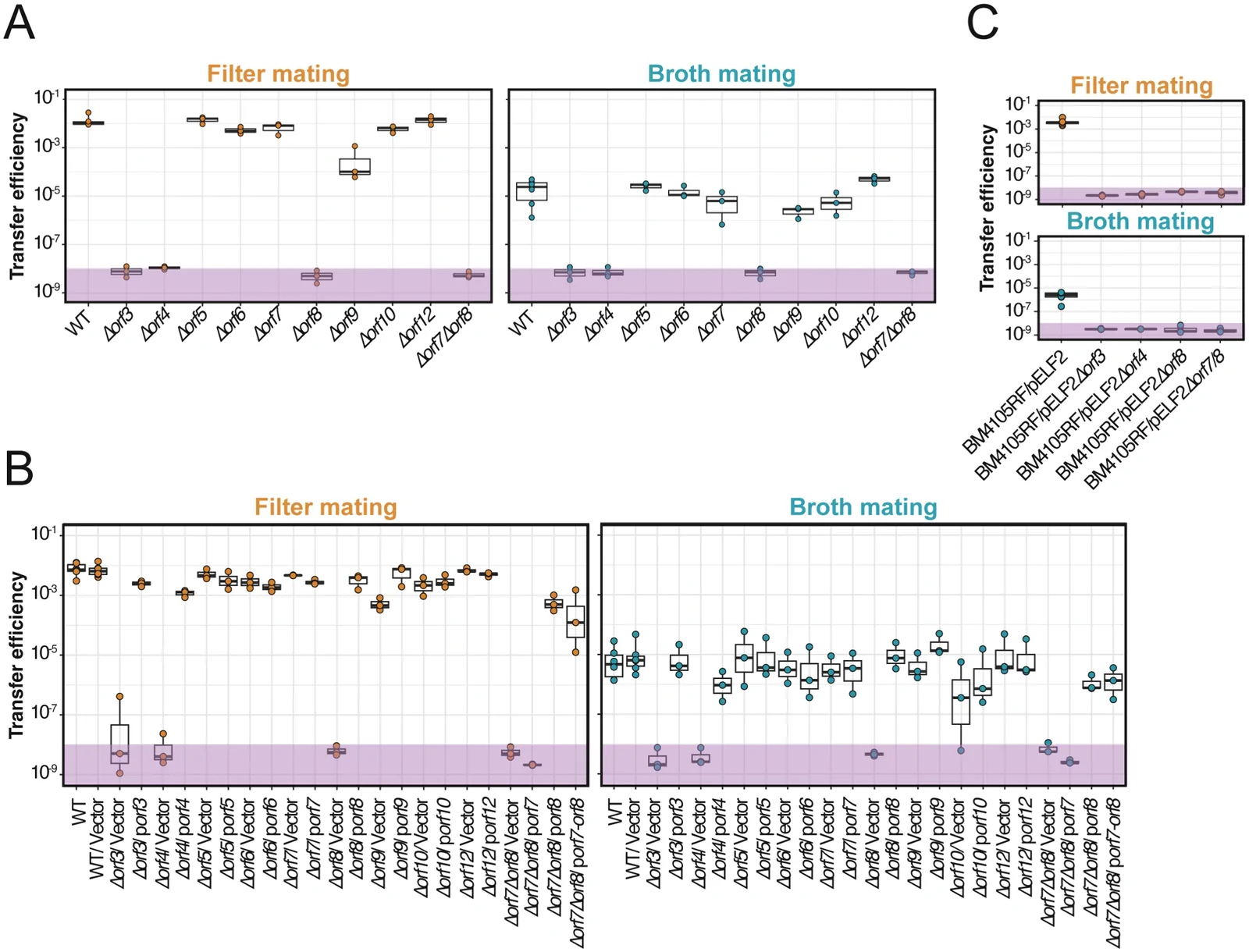
Effect of the *tra* gene deletions on the conjugative transfer of pELF2. (A) Conjugation efficiency of pELF2 plasmid derivatives with individual in-frame deletions. Donor strain KUHS13 harboring pELF2 derivatives and recipient strain BM4105RF were mixed and incubated under filter (left panel) or broth (right panel) conditions. (B) Conjugation efficiency of the pELF2 plasmid with individual in-frame deletions harboring cognate complementation vectors. Donor strain KUHS13 harboring pELF2 derivatives and recipient strain BM4105RF were mixed and incubated under filter (left panel) or broth (right panel) conditions. IPTG induction was performed during pre-incubation and co-cultivation for conjugation. The parental strain without the vector (KUHS13) and the strain harboring the empty vector (vector) are shown as controls. (C) Secondary conjugation efficiency of BM4105RF donors harboring pELF2 derivatives. Donor strain BM4105RF harboring pELF2 derivatives and recipient strain BM4105SS were mixed and incubated under filter (top panel) or broth (bottom panel) conditions. For values below the detection limit, the limit point is plotted as a proxy (purple area). Data represent the means and error bars from three independent replicates.

### Essentiality of the *tra* genes for the conjugative transfer of pELF2

Although the *tra* operon contains 12 CDSs, the encoded proteins exhibited no similarity to known plasmid-conjugated proteins. To investigate the essentiality of the *tra* genes, isogenic deletion mutants were tentatively constructed for every gene within the *tra* operon. To construct an in-frame deletion mutant without selection markers, a suicide plasmid (pMGJK72) that enabled an improved targeted genetic recombination system optimized for *E. faecium* was developed (see Materials and Methods for details; S3 Fig). Considering that the coding sequences of *orf7* and *orf8* overlap, with the 3′ end of *orf7* overlapping the 5′ end of *orf8*, a double-deletion mutant (Δ*orf7/8*) was also constructed to evaluate potential polar effects. Deletion mutants of *orf1*, *orf2*, or *orf11* could not be generated. After verification that these gene deletions did not affect bacterial growth (S4 Fig), the pELF2 deletion mutant collection was evaluated as donor strain in conjugative transfer experiments under both filter and broth conditions, using BM4105RF as the recipient strain (Fig 2A). The wild-type parent strain KUHS13 exhibited transfer efficiencies of approximately 1.0 × 10^−2^ and 1.0 × 10^−5^ in filter and broth mating, respectively. Deletion of *orf3* (∆*orf3*), *orf4* (∆*orf4*), and *orf8* (∆*orf8*) completely abolished conjugative transfer. The remaining mutants (∆*orf5, ∆orf6*, ∆*orf7*, ∆*orf9*, ∆*orf10* and ∆*orf12*) showed no notable differences compared to the wild-type strain (KUHS13) (S3 Table).

To confirm these phenotypes, an isopropyl-D-1-thiogalactopyranoside (IPTG)-inducible ectopic expression vector (pMGJK53) optimized for *E. faecium* was developed (S5 and S6 Figs) and complementation assays were performed for each deletion mutant (Fig 2B). No differences in conjugation efficiency were detected between wild-type KUHS13 with and without the control pMGJK53 vector (pMGJK53) (S3 Table). This indicated that neither the introduction of the pMGJK53 vector nor the presence of IPTG interfered with conjugative transfer efficiency. Complementation using either pMGJK53::*orf3*, pMGJK53::*orf4*, or pMGJK53::*orf8* restored the impaired conjugation phenotype in the *∆orf3*, *∆orf4* or *∆orf8* strains, respectively. Notably, the resulting transconjugants BM4105RF harboring pELF2*∆orf3*, pELF2*∆orf4*, or pELF2*∆orf8* were incapable of secondary conjugation to the BM4105SS strain (Fig 2C). This result eliminated the possibility that unintended mutations or integration events in pELF2*∆orf3, ∆orf4, ∆orf8*, or *∆orf7/∆orf8* were responsible for the restoration of the conjugation capacity. Furthermore, the ectopic overexpression of individual genes in the wild-type KUHS13 background yielded no considerable differences compared to the wild-type KUHS13 lacking an expression vector or harboring the control pMGJK53::*gfp* vector (S7 Fig and S3 Table).

Collectively, these data demonstrate that *orf3, orf4*, and *orf8* are essential for the conjugative transfer of pELF2. No involvement of *orf5*, *orf6*, *orf7*, *orf9*, *orf10* or *orf12* in the conjugative transfer of pELF2 was detected under the conditions tested. Based on these results, the essential genes *orf3*, *orf4*, and *orf8* were designated as *traC*, *traD*, and *traG*, respectively.

### Identification of a functional promoter upstream of the *tra* gene

**Fig 3:**
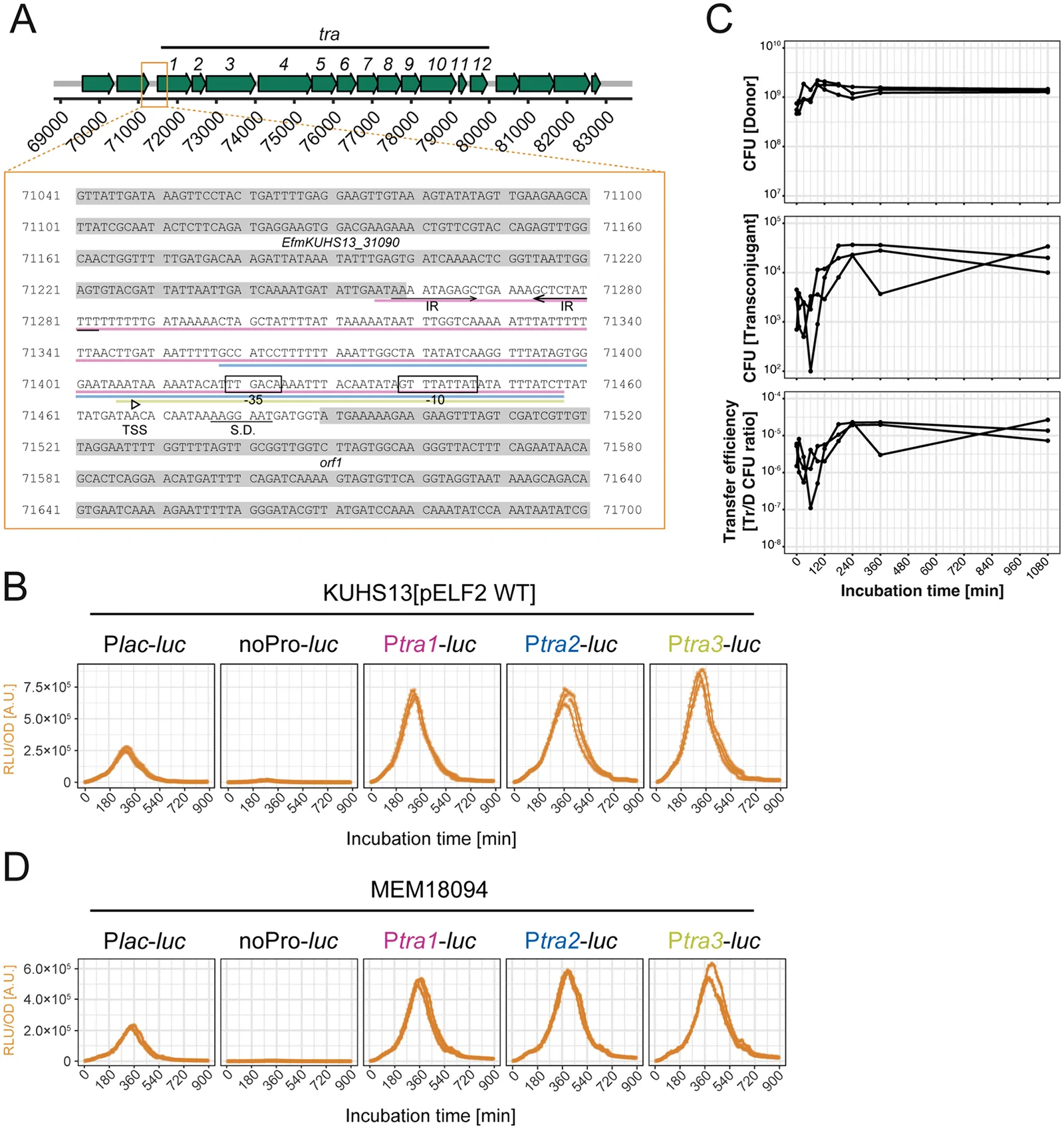
Promoter activity of the upstream region of the putative *tra* operon. (A) Nucleotide sequence of upstream region of *orf1* gene. The putative transcription start site (TSS) was determined from the RNA-seq coverage. Cloned promoter regions (P1, P2, and P3) are highlighted with pink, blue and yellow underlines, respectively. (B) Kinetics of promoter activation. OD-normalized bioluminescence activity of the wild-type KUHS13 harboring pMGJK55::P*tra*1 (pink), pMGJK55::P*tra*2 (blue), or pMGJK55::P*tra*3 (yellow) in the presence of 10 µg/mL D-luciferin. Plots represent values of three replicates. (C) Kinetics of pELF2 transfer. Broth mating experiment was performed as described above, with sampling for plating at each time point. Plots represent values of three replicates. (D) Kinetics of promoter activation as panel B but MEM18094,which is clade A1 strain without pELF-type plasmid, was used as host instead KUHS13. Plots represent values of three replicates.

Pheromone-responsive conjugative plasmids in *E. faecalis* are the most widely studied type of enterococcal plasmids, and their conjugation is transcriptionally induced in response to quorum-sensing peptide pheromones produced by recipient cells. The operon-like expression pattern observed in the RNA-seq data suggests a transcriptional start site upstream of the orf1 gene, at position 71,468 in the AP022343.1 reference sequence (Figs 1 and 3A). To assess the putative promoter activity of the *tra* operon, fragments of the putative promoter region were cloned into the pMGJK55 plasmid, which is a luciferase reporter vector designed for promoter assays (S8 Fig). Fragments P*tra*1 (pink), P*tra*2 (blue), and P*tra*3 (yellow) were generated by amplifying regions from positions 71,256–71,457, 71,358–71,457, and 71,406–71,457 of the pELF2 plasmid, respectively (Fig 3A). Wild-type KUHS13 was transformed with reporter constructs, and bioluminescence activity was monitored during bacterial growth using a plate reader (Fig 3B). In this experimental setup, it is noted that a technical lag between the initiation of reporter (luciferase) transcription and signal detection, which involves time for protein maturation and substrate (D-luciferin) degradation. Consequently, even for the constitutive positive control, signal peaks were detected only 3–4 hours after the start of measurement. The promoter-less negative control construct produced no detectable bioluminescence signals. All tested promoter-fragment constructs exhibited similar kinetics but higher signal intensities than those of the positive control (P*lac* promoter in the absence of the *lacI* repressor gene) (Fig 3B).

The kinetics of conjugative transfer in broth conditions also supported a constitutive expression model; transconjugants were generated immediately after mixing, suggesting that the transfer machinery is already pre-assembled during monoculture (Fig 3C). On the other hand, a temporary decrease in transfer frequency was observed between 30 and 120 minutes, corresponding to the exponential growth phase. This likely reflects a transient “dilution” of the conjugation machinery components, as the rate of its assembly may not keep pace with the speed of active cell division. These observations suggest that the putative *tra* operon is expressed constitutively, independent of the presence of recipient cells or the growth phase, which is consistent with the transcriptomic results described above (Fig 1).

To investigate whether P*tra* activity is affected by elements encoded on the pELF2 plasmid, we performed promoter assays in *E. faecium* MEM18094, a clade A VRE strain that lacks pELF-type plasmid (Fig 3D) [26]. Robust P*tra* promoter activity was observed in MEM18094 to an extent completely comparable to that in KUHS13, strongly suggesting that P*tra* activity is independent of the pELF-type plasmid.

### Structural modeling of TraC and TraD

**Fig 4:**
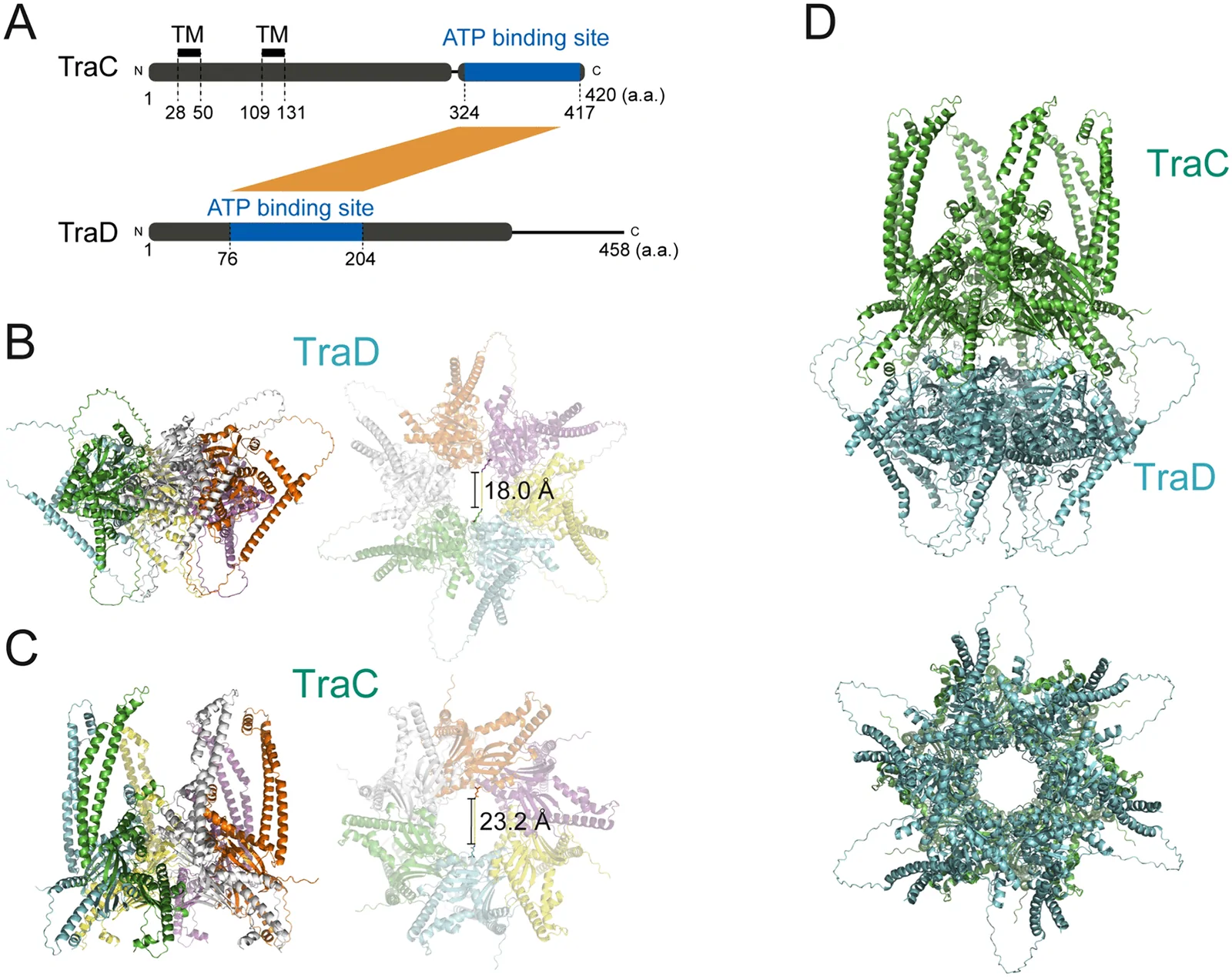
Protein structure modeling of TraC and TraD ATPase for conjugation. (A) Molecular structures of TraC (420 amino acids, top) and TraD (458 amino acids, bottom) proteins with conserved functional domains. TraC and TraD contain an ATP-binding domain, whereas TraC contains two transmembrane domains. The orange link indicates predicted protein–protein interactions. (B, C) Protein structure modeling of TraD (B) or TraC (C) homohexamer generated from six copies of each protein sequence using AlphaFold3. Diameters of the pore ring complex estimated based on predicted model were shown. (D) TraC and TraD each form stable homohexamers ring complex.

Generally, the VirD4-type ATPase serves as a core component of the conjugation machinery spanning the cytoplasmic membrane of the donor cell and acts as a coupling protein that links the DNA substrate to the secretion pore [50]. Hidden Markov Model-based searches and amino acid sequence analysis indicated that TraD is a homolog of the VirD4/FtsK-type ATPase family, thus sharing conserved motifs required for DNA translocation, but lacking transmembrane domains (Fig 4A). In contrast, although TraC exhibited no significant sequence similarity to known proteins, a distinct ATPase domain was detected at its C-terminus, and two transmembrane helices were identified (Fig 4A). To gain further mechanistic insights, structural modeling of TraC and TraD was performed using AlphaFold3. Consistent with other FtsK/VirD4-family proteins, TraD was predicted to form a homohexameric ring (Fig 4B), whereas TraC was predicted to form a stable hexamer (Fig 4C). Protein docking simulations suggested that these hexamers assembled into stable stacked ring complexes (Fig 4D).

### Phylogenetic and pangenomic landscape of pELF-type plasmids

**Fig 5:**
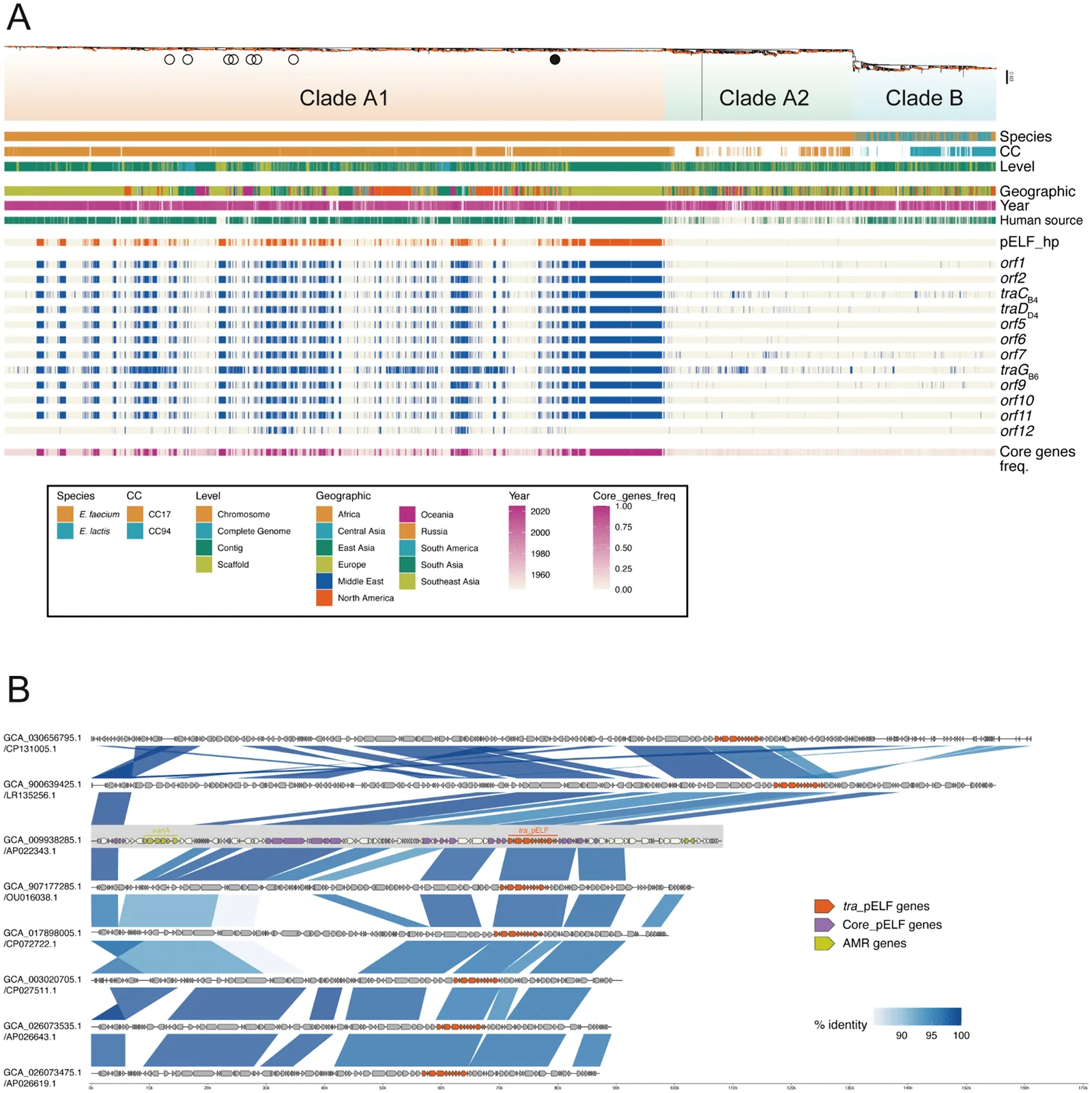
Phylogenetic analysis of the tra genes among pELF-like plasmid sequences (A) Neighbor-joining tree based on pairwise Mash distances of 9,174 E. faecium (Taxonomy ID: 1352) and E. lactis (Taxonomy ID: 357441) genomes. Orange dots at nodes indicate jackknife support values: light dots ≥50% and solid dots ≥70%. Scale bar: 0.03 Mash distance. Metadata annotations (species, assembly level, geography, year, human source) were retrieved from NCBI. CC designations and *tra/core* gene detection (BLASTn, ≥ 70% nucleotide identity) are described in Materials and Methods. For the detection of core genes excluding the *tra* genes, the number of hits relative to the total number of core genes was scored and displayed as “core gene frequency” (n = 36). Closed and open dots indicate the reference strain KUHS13 (pELF2) and other pELF-like plasmid contigs selected for synteny analysis in panel B, respectively. (B) Synteny comparison of pELF-like plasmid contigs. Arrows indicate CDSs; homologous regions identified by all-versus-all BLASTn are shown as links (length ≥ 3 kb, identity ≥ 80%). Link shading indicates percent identity. CDSs are colored by category: *tra* operon (red), pELF-core genes (purple), antimicrobial resistance genes (yellow), and reference pELF2-specific annotations (custom colors); CDSs on other plasmids are shown in grey.

To elucidate the prevalence of pELF-type plasmids within *Enterococcus* species, a large-scale comparative genomic analysis was conducted using 9,174 genomes of *E. faecium*/*E. lactis* complex, encompassing all assembly levels from draft to complete (S4 Table). The phylogenetic tree based on pairwise Mash distances provided a comprehensive overview of the genomic diversity of *E. faecium* and *E. lactis* (Fig 5A). The population structure consistently exhibited a three-cluster arrangement, as previously reported. Specifically, Clade A1 predominantly comprised the human-associated high-risk clone CC17. Clade B included a mixture of *E. faecium* and *E. lactis* associated with CC94. This mixed composition is likely due to the historical classification of these strains before E. lactis was formally recognized as a distinct species, as the registered *E. faecium* strains in this clade actually share closer phylogenetic proximity to the *E. lactis* type strain. Clade A2 was phylogenetically related to Clade A1 but typically originated from non-human (animal and environmental) sources.

To identify pELF-type-positive genomes, the presence of the 500-bp left-hand hairpin nucleotide sequence (pELF_hp), which is a hallmark of pELF-type plasmids, was used as a diagnostic marker, along with the distribution of the 12 open reading frames in the *tra* operon (Fig 5A). Mapping revealed that the pELF-type plasmids were substantially enriched within Clade A1, where they co-occurred with most *tra* genes. A small number of *traG*-like hits were also detected in genomes lacking pELF-like plasmids; a possible explanation is discussed below. Statistical analysis further demonstrated that the presence of pELF_hp strongly correlated with the enrichment of *tra* operon-encoded genes and pELF core genes across the entire dataset (S9 Fig). Although other common enterococcal plasmid replicons (e.g., *rep2*, *repUS7*, *repUS43*, *rep11a*, *repUS15*, and *rep17*) showed no pronounced association with these components, the pELF-type marker (pELF_hp) served as a highly reliable indicator of the presence of the *tra* operon and associated core genes (S9 Fig).

To investigate syntenic preservation of the *tra* operon, complete genome assemblies were analyzed to extract plasmid contigs harboring pELF_hp (S10 Fig). Pangenomic analysis of these contigs was performed using Roary, with a 70% BLASTp identity threshold to accommodate the characteristic sequence divergence of plasmid-encoded proteins. A total of 45 core genes defined by a prevalence of ≥80% across all pELF-type plasmids were identified, including plasmid maintenance genes and 9 of the 12 tra operon genes (S5 Table). While most genes in this core set were of unknown function, the inclusion of most *tra* genes underscores the stability of the conjugation system. Three *tra* genes fell below the core gene threshold: *orf1* was detected in 72.5% of pELF-like plasmids, falling marginally below the threshold; *orf11* was initially underestimated owing to its small size in Roary-based pangenomic analysis, but manual BLASTn searches confirmed its high prevalence, consistent with other *tra* genes; and *orf12* was confirmed as a rare accessory gene (Fig 5A). Furthermore, synteny plots of eight representative plasmids selected from diverse phylogenetic positions confirmed that the *tra* genes were consistently encoded as conserved blocks (Fig 5B). Collectively, these findings strongly suggest that the pELF-type conjugation system is highly stable and is predominantly maintained within the high-risk enterococcal lineages associated with human clinical settings.

## Discussion

In this study, RNA-seq data revealed a putative operon likely related to the conjugative transfer of pELF2 was identified. We designated this operon as the pELF-type linear plasmid-related *tra* operon. The operon encodes 12 CDSs, *orf1–12*, including the VirD4/FtsK-type ATPase motor homolog *traD*. By constructing isogenic gene deletion derivatives of the pELF2 plasmid, the study confirmed that *traD* is essential for the conjugative transfer of the pELF2 plasmid. Additionally, the hypothetical protein-encoding genes *traC* and *traG* were found to be novel essential conjugative genes of pELF2. The large-scale analysis of 9,174 genomes further revealed that the *tra* operon is remarkably conserved (11 of 12 genes detected across pELF-like plasmid-positive genomes) and markedly enriched in the human-associated high-risk Clade A1 lineage. However, a small number of *traG*-like hits were detected in genomes lacking pELF-like plasmids (Fig 5A). This likely reflects cross-detection of chromosomally encoded rhomboid-family proteases, as *traG* contains a rhomboid intramembrane protease domain that is widely conserved across bacterial genomes. Although these genes are highly conserved within the synteny block across pELF-like plasmids, deletion mutants of *orf5, orf6, orf7*, *orf9*, *orf10* and *orf12* were obtained but did not show statistically significant effects on conjugation (Fig 2 and S3 Table). The strong conservation of these genes, despite the lack of a discernible mutant phenotype, suggests they may fulfill essential roles in natural environments that are not captured under the current in vitro experimental conditions. Although *orf12* is located at the 3’ terminus of the putative operon, its distinct transcriptional profile, which is characterized by decreased expression during the stationary phase (Fig 1B), combined with its low phylogenetic conservation and lack of a mutant phenotype, suggests that *orf12* is not a functional component of the core Tra system. The roles of *orf1*, *orf2,* and *orf11* in the conjugative transfer of pELF2 were not assessed in this study because deletion mutants of these genes could not be constructed. Therefore, this aspect remains a subject for future research.

Promoter analysis of the region upstream of *orf1* suggested that the transcription of the putative *tra* operon is coordinated by at least a 100-bp region from the inferred transcriptional start site (Fig 3). Transcription appeared to be constitutively induced throughout bacterial growth, regardless of the presence of recipient cells (Fig 1). Recipient-dependent induction of conjugative gene expression has been well studied in pheromone-responsive conjugative plasmids in *E. faecalis* [51]. In this system, a peptide composed of eight amino acids is chromosomally produced by *E. faecalis* and recognized by a cognate pheromone-responsive plasmid to induce the transcription of the conjugative operon [52,53]. However, induction modules stimulated by external signals have not been identified in *E. faecium* conjugative plasmids (e.g., pMG1-like plasmids) [54]. Such a mechanism may be discovered in the future as research on *E. faecium* plasmids progresses. Furthermore, direct evidence, such as Cappable-Seq, 5′-RACE assay, or northern hybridization, is necessary to establish transcriptional operon structure for the *tra* gene cluster.

In most bacterial conjugative machinery, two distinct multimeric ATPases, typically referred to as VirD4 (the coupling protein) and VirB4, play central roles as core engines of the DNA translocation complex [55]. These ATPases coordinate to provide the mechanical energy required for the assembly of the secretion apparatus and active transport of the DNA substrate across the cell envelope. The study proposed that TraC and TraD in pELF2 function as essential motor units corresponding to the VirB4/VirD4-like assembly. Protein structure modeling using AlphaFold3 predicted a stable interaction between the C-terminal portion (approximately 300–400 a.a. out of 420 a.a.) of TraC and the ATPase domain (approximately 30–320 a.a. out of 458 a.a.) of TraD (Fig 4), thereby suggesting that TraD was anchored to the cytoplasmic membrane via its interaction with TraC. Notably, our experimental and computational data strongly suggest that TraC is a functional analog of the VirB4-like ATPase. Although it lacks substantial sequence homology with conventional VirB4, TraC is essential for transfer, belongs to the FtsK family of ATPases, and is predicted to adopt a homohexameric architecture. Furthermore, the study modeling suggests a stacking arrangement of TraC and TraD hexamer complexes (Fig 4D), which is highly reminiscent of the dual-ATPase motor organization characterized by canonical T4SSs.

Although no direct physical interaction was predicted between TraG and the TraC/D motor complex, TraG likely represents a functional analog of the VirB6-like scaffold. VirB6 is an inner membrane-embedded protein that contributes to the formation and stabilization of the T4SS complex in gram-negative bacteria. However, VirB6 does not necessarily participate as a direct component of the central ATPase assembly. This is consistent with the observation of TraG functioning independently of the TraC/D motor stack. Interestingly, TraG exhibited structural similarity to rhomboid-family proteases. This suggests that it may form a specialized membrane channel. In accordance with the recently proposed unified nomenclature for T4SS subunits, we propose the designations *traD*_D4_, *traC*_B4_, and *traG*_B6_ [56]. We note that this assignment rests on different levels of evidence: TraD shows clear sequence-level homology to the VirD4/FtsK-type ATPase family, whereas TraC and TraG lack detectable sequence homology to VirB4 and VirB6, and their designations are based on architectural and functional analogy rather than demonstrated orthology.

These features are broadly consistent with the evolving model of conjugation systems identified in various gram-positive bacteria (*Streptomyces* and *Mycobacteria*), *Mycoplasma*, *Thermococcus*, and *Pseudomonas aeruginosa* [33,35,57–60]. In these systems, a minimal FtsK-type motor unit, centered on a universally conserved translocator (VirD4/FtsK-like), in some cases accompanied by a partner ATPase (VirB4-like), functionally interacts with variable numbers of integral membrane proteins (putative functional counterparts of VirB6 or VirB8) to mediate the transfer of linear fragments of chromosomes, plasmids, or integrative conjugative elements. This shared architectural principle is further supported by recent studies on the *Streptomyces* linear plasmid SAP1, in which a minimal core consisting of two FtsK-family proteins was identified as sufficient for DNA translocation [35]. While these minimal translocation modules share functional similarities, their underlying genetic architectures vary significantly across different bacterial species (S11 Fig). Furthermore, the topology of the transferred DNA substrates is highly diverse, encompassing circular or linear plasmids as well as Integrating and Conjugative Elements (ICEs). This suggests that such noncanonical minimal transfer systems may represent versatile modules that are widely distributed across diverse biological lineages. Our observations regarding the TraC_B4_/D_D4_/G_B6_ system in pELF2 reinforce the hypothesis that these noncanonical conjugation systems across different domains of life utilize a variable yet functionally analogous motor–channel module to drive the transfer of diverse DNA substrates.

In general, bacterial conjugative plasmid transfer is performed using a relatively large protein complex that localizes to the donor cell membrane to form a molecular channel that passes the plasmid DNA substrate to the recipient cell [29]. The classical conjugative transfer model for circular plasmids states that a plasmid-encoded nickase generates single-stranded DNA and loads it into the conjugative T4SS [46,61]. However, because this model relies on the rolling-circle replication (RCR) mechanism, the model is not applicable to the transfer of linear plasmid DNA. In contrast to conventional circular plasmids, knowledge of the conjugation mechanisms of linear plasmids is limited. However, genetic studies of SLP2 from *Streptomyces* have provided insights into linear plasmid transfer. Conjugation of SLP2 is thought to occur via the first-end model, in which the linear plasmid molecule is transferred to the recipient in a double-stranded form, starting from one end [33]. Given that no nickase homolog was present in the pELF-type plasmids, a first-end model similar to that of SLP2 appears to be the most plausible explanation.

Interestingly, the diameter of the ring pore in the TraC_B4_/D_D4_ complex (estimated as 23.2 Å for TraC and 18.0 Å for TraD) is close to that of the FtsK-like ATPase ring in SLP2 (14.5 Å) and the recently described TcpA from *Clostridium perfringens* pCW3 (22.0 Å), while being substantially larger than that of the circular pIP501 plasmid (8.2 Å) (S12 Fig). This difference likely reflects distinct substrate sizes. The pIP501 plasmid transfers single-stranded DNA via an RCR-like mechanism, which further supports the idea that pELF-type plasmids, such as SLP2 and pCW3, are transferred via the first-end model as double-stranded DNA substrates [33,62,63].

Genetic manipulation is a powerful method to establish direct evidence of gene function. In *E faecalis*, sophisticated genetic engineering tools, established based on the knowledge of pheromone-responsive conjugative plasmids, have allowed extensive investigation of plasmid and cellular functions in *E. faecalis* [64]. Despite the recent increase in its clinical importance, genetic engineering methodologies for *E. faecium* remain underdeveloped [65]. In this study, effective genetic engineering tools were successfully developed and implemented for *E. faecium* and are expected to provide new options for genetic studies and advance the field [66,67]. Further understanding of the conjugation mechanism through the identification of minimal essential components will require the construction of a minimal plasmid system, as previously demonstrated for the most extensively studied linear plasmid transfer system SAP1. Achieving this for the pELF family necessitates extensive research on the comprehensive plasmid life cycle, including replication, partitioning, and conjugative transfer. The pELF-type linear plasmid family is one of the most common plasmid families in *E. faecium*, and acts as a carrier of critical ARGs worldwide. The insights into plasmid biology established in this study will facilitate improved understanding of plasmid-mediated adaptive evolution of *E. faecium* strains.

## Materials and methods

### Bacterial strains, plasmids, and antimicrobial agents

The bacterial strains, plasmids, and oligonucleotides used in this study are listed in S6, S7, and S8 Tables, respectively. Enterococcal strains were routinely grown in BHI broth (Difco, Detroit, MI, USA) at 37°C [68] or 30°C to maintain temperature-sensitive plasmids. Negative selection for the plasmid-harboring strain pheS* was performed on MM9YG agar containing 10 mM *p*-chlorophenylalanine. *Escherichia coli* strains were grown in Luria-Bertani medium (Difco) at 37°C or 30°C to maintain temperature-sensitive plasmids. The antibiotics used to select *E. coli* included 100 mg/L ampicillin, 30 mg/L chloramphenicol, and 20 mg/L gentamicin (GEN). The concentrations used for the routine selection of *E. faecium* harboring pMGJK72, pMGJK53, or pMGJK55 derivatives were 500 mg/L GEN. All antibiotics were obtained from Sigma-Aldrich (St. Louis, MO, USA).

### Total RNA isolation

An overnight bacterial culture was inoculated into BHI at a 10-fold dilution and pre-incubated at 37°C for 1 h. Subsequently, 500 µL of the preculture broth was inoculated into 4.5 mL of fresh BHI broth and incubated at 37°C for 4 or 8 h to reach exponential and stationary phases, respectively. For the co-culture conditions, the recipient strain was pre-incubated and mixed at an equal volume. After incubation, an equal volume (5 mL) of RNAprotect reagent (Qiagen, Hilden, Germany) was added to the culture broth and mixed via inversion. Stabilized cells were collected via centrifugation at 5,000 × *g* for 10 min. Total RNA was extracted from the pellet using an RNeasy Plus Kit (Qiagen) according to the manufacturer’s instructions. The obtained RNA was analyzed using a Qubit fluorometer or nanophotometer to determine its concentration and purity, respectively.

### RNA sequencing and data analysis

Starting with 100 ng of total RNA, rRNA was depleted using the NEBNext rRNA Depletion Kit (Bacteria) (NEB, Ipswich, MA, USA). Stranded cDNA libraries were prepared using the Illumina Stranded mRNA Prep Ligation kit (Illumina, San Diego, CA, USA) according to the manufacturer’s instructions and sequenced on a NovaSeq 6000 platform using the NovaSeq 6000 SP Reagent Kit v1.5 (300 cycles) (Illumina) with a 151-bp paired-end protocol. Raw reads were analyzed using FastQC and mapped to the pELF2 sequence (AP022343.1) using Bowtie2 (v.2.5.3) with default parameters. Differential expression analyses were performed using DESeq2 (v.1.48.0). Data visualization was performed using the ggplot2 package in R.

### Plasmid constructions

#### Construction of the pMGJK72 plasmid and its derivatives for genetic manipulation.

To construct a vector for improved site-directed genetic engineering of *E. faecium*, three DNA fragments were assembled. The first fragment, which contained the temperature-sensitive origin (*ori*_*pWV01*_*TS*), was polymerase chain reaction (PCR)-amplified using the primer pair (JK773/JK804) and pGPA1 [69] as a template. The second fragment, containing the GEN resistance gene *aacA-aphD* for selection (*gen*^*r*^) was PCR-amplified using a primer pair (JK774/JK747) with pGPA1 as a template. The third fragment containing the suicide gene (a mutant form of *pheS*) and a multiple cloning site (MCS) for Golden Gate Assembly (pheS* + MCS) was PCR-amplified using a primer pair (JK465/JK748) and pMGJK09 as a template. The first fragment was digested with *Bsa*I, and the second and third fragments were digested with *BsmB*I. The fragments were ligated using T4 DNA ligase. The resulting plasmid after GEN selection in *E. coli* HST08 was confirmed via whole-plasmid sequencing and designated pMGJK72.

To construct pMGJK72 derivatives for site-directed mutagenesis of each *tra* gene, 1-kb DNA fragments flanking the upstream or downstream regions of each gene were PCR-amplified using the primer pairs JK857/JK858 and JK934/JK860, JK898/JK899 and JK900/JK901, JK902/JK903 and JK904/JK905, JK906/JK907 and JK908/JK909, JK930/JK931 and JK1151/JK933, JK938/JK936 and JK912/JK913, JK914/JK915 and JK916/JK917, JK918/JK919 and JK920/JK921, JK926/JK927 and JK928/JK929, or JK930/JK931 and JK912/JK913 for *orf3*, *orf4*, *orf5*, *orf6*, *orf7*, *orf8*, *orf9, orf10, orf12* or *orf7/orf8*, respectively and the KUHS13 genomic DNA as a template. The resulting fragments and pMGJK72 plasmid were digested with *Bsa*I and ligated using T4 DNA ligase. The resulting plasmids were selected GEN in *E. coli* HST08 and confirmed via Sanger sequencing using the primer pair of JK881 and JK882.

#### Construction of the pMGJK53 plasmid and its derivatives for IPTG-inducible expression.

To construct IPTG-inducible expression, a linear DNA fragment was generated using the primer pair JK704/JK705 and the pRecT plasmid as a template. The DNA fragment containing the entire pRecT plasmid, [66] excluding the *recT* gene, was digested with *Bsa*I and dephosphorylated using Quick CIP (NEB). The superfolder *gfp* (*sfgfp*) gene was PCR-amplified using the primer pair JK532/JK533 and a synthetic DNA fragment (Integrated DNA Technologies, Inc., Coralville, IA, USA) as a template. The obtained *sfgfp* DNA fragment was digested with *BsmB*I and ligated into the pRecT-derived DNA fragment using T4 DNA ligase. The resulting plasmid after spectinomycin selection in *E. coli* HST08 was designated pMGJK51. Notably, the cloned *sfgfp* gene contained an internal *Bsa*I site that generated sticky ends for AGTC and GATT for the Golden Gate Assembly.

To promote the expression efficiency of the cloned genes via efficient transcriptional termination, pMGJK51 was linearized via PCR using the primers JK533 and JK708, followed by digestion with *Bsa*I. The *rrnB* terminator sequence was amplified using the primer pair JK530/JK531 and pTurbo as templates, followed by digestion with *BsmB*I. The *rrnB* terminator fragment was ligated into the linearized pMGJK51 using T4 DNA ligase. The resulting plasmid selected for spectinomycin in *E. coli* HST08 was designated pMGJK52.

To replace the spectinomycin selection marker with the GEN selection marker, pMGJK52 was linearized via PCR using the primer pair JK711/JK712. The obtained DNA fragment, which contained the entire pMGJK52 plasmid, except for the *spc*^*r*^ gene, was digested with *Bsa*I. A fragment of the GEN -resistance gene *aacA-aphD* for selection (*gen*^*r*^) was amplified using the primer pair JK524/JK525 with pGPA1 as a template. The obtained *gen*^*r*^ DNA fragment was digested with *Bsa*I and ligated into a pMGJK52-derived DNA fragment using T4 DNA ligase. The resulting plasmid, after GEN selection in *E. coli* HST08, was confirmed by whole-plasmid sequencing and designated pMGJK53.

To construct pMGJK53 derivatives for ectopic expression of individual *tra* genes, each gene was PCR-amplified using a combination of primer pairs JK993/JK994, JK995/JK996, JK885/JK886, JK997/JK998, JK999/JK1000, JK1001/JK1002, JK1013/JK1014, JK1003/JK1004, JK1005/JK1006, JK1007/JK1008, JK1009/JK1010, JK1011/JK1012, and JK1013/JK1004 for *orf1*, *orf2*, *orf3*, *orf4*, *orf5*, *orf6*, *orf7*, *orf8*, *orf9, orf10*, *orf11*, *orf12*, and *orf7*/*orf8*, respectively, using KUHS13 genomic DNA as a template. The resulting fragments and the pMGJK53 plasmid were digested with *Bsa*I and ligated using T4 DNA ligase. The resulting plasmids after GEN selection in *E. coli* HST08 were confirmed via Sanger sequencing using the primer pair of JK1061 and JK1062.

#### Construction of the pMGJK55 plasmid and its derivatives for promoter assay with luciferase reporter gene.

To construct a promoter activity reporter plasmid based on pMGJK53, a synthetic luciferase gene codon-optimized for *L. lactis* was PCR-amplified using the primer pair JK757/JK758 (Integrated DNA Technologies Inc.). The amplified fragment was then ligated into pMGJK53 via Golden Gate Assembly using *Bsa*I as described above. To remove the *lacI* repressor gene, pMGJK53::*luc* was linearized via PCR using JK735 and JK812 and subsequently re-circularized via sequential treatment with *Bsa*I and T4 DNA ligase. The resulting plasmid pMGJK53::*luc*_∆*lacI* was again linearized via PCR using the primer pair JK745/JK746 and digested with *Bsa*I. Annealed oligonucleotides JK1015 and JK1016 were digested with *Bsa*I and mixed with the linearized pMGJK53::*luc*_∆*lacI*, followed by ligation using T4 DNA ligase. After GEN selection in *E. coli* HST08, the resulting plasmid was confirmed via whole-plasmid sequencing and designated pMGJK55.

To construct the pMGJK55 derivative harboring the *tra* promoter regions (P*tra*), the upstream regions of *orf1* of various lengths, P*tra*1, P*tra*2, or P*tra*3, were PCR-amplified using primers JK1022 and JK1019, JK1020, and JK1021, respectively, and KUHS13 genomic DNA as a template. These fragments were assembled via digestion with *Bsa*I, ligated using T4 DNA ligase, and subsequently cloned into pMGJK55 as described above. The resulting plasmids after GEN selection in *E. coli* HST08 were confirmed via Sanger sequencing using the primer pair of JK1017 and JK1018.

### Site-directed recombination using pMGJK72 derivatives

The pMGJK72 derivatives were introduced into *E. faecium* KUHS13 via electroporation, followed by selection on BHI agar containing GEN (500 mg/L) at 30°C. Selected colonies obtained at the permissive temperature (30°C), where the plasmids are supposed to be maintained outside the host genome, were then streaked on pre-warmed BHI agar containing GEN (500 mg/L) and incubated at 42°C. Visible colonies obtained at a non-permissive temperature, where the plasmids were supposed to be integrated into the host genome, were collected and restreaked at least twice under the same conditions to ensure integration. The resulting colonies were inoculated into 5 mL of BHI broth without selection and incubated at 37°C overnight. A 100-fold dilution of the overnight culture (100 µL) was plated on MM9YG agar containing 10 mM *p*-chlorophenylalanine, followed by incubation at 37°C overnight. Colonies obtained from negative selection, in which the plasmids were supposed to be cured, were genotypically screened using PCR. Desired genetic deletions were confirmed using Sanger sequencing.

### Conjugative transfer

Conjugation assays were performed as previously described [68]. KUHS13 and its isogenic derivatives were used as the donor strains, and *E. faecium* BM4105RF was used as a recipient strain. Briefly, donor and recipient strains were grown overnight in BHI at 37°C. The overnight culture was diluted 10-fold in fresh BHI and incubated for an additional 1 h at 37°C. A mixture was prepared by combining 250 μL of donor and recipient cultures. For filter mating, 500 µL of the donor and recipient mixture was passed through a 0.22-μm nitrocellulose filter (Merck Millipore, Darmstadt, Germany) and placed onto BHI agar without antibiotic selection. After incubation at 37°C for 5 h, the bacterial cells on the nitrocellulose filter were collected in 1 mL of phosphate-buffered saline (PBS) using vortexing. For broth mating, 500 µL of the donor and recipient mixture was incubated in a microcentrifuge tube without agitation at 37°C for 6 h. After incubation under either condition, serial dilutions of the cell suspension were plated on selective BHI agar containing rifampicin (25 mg/L), fusidic acid (25 mg/L), and VAN (16 mg/L) or BHI agar containing VAN (16 mg/L) to count the colonies of the transconjugant or donor, respectively. The plates were then incubated at 37°C for 48 h. The colony count on the agar plate was determined, and the estimated ratio of the transconjugant colony-forming unit count to that of the donor was calculated as conjugative transfer efficiency.

For statistic evaluation, conjugation frequencies were compared between each donor strain and the wild-type reference using paired *t*-tests on log₁₀-transformed values (n ≥ 3 biological replicates). A pseudocount (one-tenth of the minimum observed positive value) was added prior to log-transformation. *P*-values were adjusted for multiple comparisons using the Benjamini–Hochberg method. Exact adjusted *P*-values are reported. Samples in which the majority of replicates fell below the detection limit were not subjected to statistical testing. All statistical analyses were performed in R (v4.5.0).

### Growth analysis and luciferase expression assay

To measure the kinetics of bacterial growth and reporter gene expression, an overnight culture of the *E. faecium* strain was inoculated into fresh BHI medium at a 100-fold dilution. To detect bioluminescence activity derived from the luciferase reporter gene expression, D-luciferin was added to the medium at a final concentration of 10 µg/mL. The bacterial suspension (200 µL) was aliquoted into each well of a white 96-well microplate with a clear bottom, with three replicates per sample. Bioluminescence and optical density at 620 nm were monitored every 10 min during incubation at 37°C using a heater-equipped plate reader (Spark, Tecan, Switzerland).

### Protein structural prediction and interaction analysis

Structural models for *tra* gene products and their potential interactions were predicted using AlphaFold 3 with default settings (AlphaFold Server, Google DeepMind). The reliability of the generated models was evaluated based on the predicted local distance difference test scores and predicted aligned error (PAE) matrices. High-confidence interactions were identified based on low interdomain PAE values and visual inspection of the interface. All structural visualizations and diameter estimations of the protein complex pores were performed using PyMOL (v2.5).

### Phylogenetic analysis

All available nucleotide sequences of *E. faecium* (Taxonomy ID: 1352) and *E. lactis* (Taxonomy ID: 357441) were retrieved from the NCBI GenBank microbial genome database on 18^th^ March 2026, using SeqKit (v.2.10.0) [70]. For phylogenetic reconstruction, pairwise genomic distances were estimated using Mash (v.2.3), with a k-mer size of 21 and a sketch size of 10,000 [71]. A neighbor-joining (NJ) tree was inferred from the resulting Mash distance matrix using the ape (v.5.8-1) package in R [72]. A subsampling (jackknife-like) pseudo-support procedure was employed to assess node support for this large-scale dataset. Briefly, in each of the 50 replicates, 80% of the genomes were randomly sampled without replacement, and an NJ tree was reconstructed from the corresponding distance submatrix. The support value for each internal clade in the full NJ tree was defined as the percentage of replicates from which the same clade was recovered. The final phylogenetic tree, integrated with metadata and gene presence/absence profiles, was visualized using the ggtree package [73,74]. From the total sequence pool, contigs harboring the 500-bp terminal hairpin sequence of pELF2 were identified using BLASTn (v.2.16.0) [75] and categorized as pELF-like plasmids. The plasmid sequences were annotated using Prokka (v.1.14.6) [76]. Pangenomic analysis was performed using Roary (v.3.13.0) with a 70% amino acid identity parameter setting [77]. A total of 45 genes with a prevalence of ≥80% across pELF-type plasmids were defined as core genes, of which 9 correspond to tra operon genes (*orf2*–*orf10*). Three additional *tra* genes (*orf1*, *orf11*, and *orf12*) fell below the core gene threshold but were included in the conservation analysis. Clonal complex (CC) designations were determined using pubMLST (https://pubmlst.org/). The presence of tra operon genes (orf1–12) and pELF-core genes (n = 36, excluding the *tra* genes) was assessed by BLASTn with a threshold of ≥70% nucleotide identity. The core gene frequency was calculated as the number of detected core genes divided by the total number of core genes. Synteny analysis and visualization were performed using the gggenomes package (v. 1.1.3) [78].

## Supporting information

S1 Fig Growth curve of *E. faecium* KUHS13 strain in BHI broth. The overnight cultures of the *E. faecium* KUHS13 strain was inoculated into fresh BHI broth at a 10-fold dilution, followed by incubation at 37°C for 1 h. The preculture was further inoculated into fresh BHI broth at a 10-fold dilution, followed by incubation at 37°C with monitoring of turbidity every 10 min during the incubation period. The time point of 4 h or 8 h was indicated as sampling point for RNA-seq analysis, which is shown in main Fig 1. Data points from an experiment performed in triplicate are shown.
(TIF)

S2 Fig Differential expression of pELF2-encoded genes among various conditions. Volcano plot presents differential expression of pELF2-encoded genes between conditions of exponential and stationary phase (A) or with or without recipient cells (BM4105RF) (B).
(TIF)

S3 Fig Plasmid structure of pMGJK72. Plasmid map of constructed plasmid vector for targeted genetic recombination, pMGJK72. Multiple cloning site (MCS) is designed for Golden Gate cloning where *Bsa*I digestion generates sticky end with AGTC and GATT. Adjacent *Eco*RI or *Bam*HI site is unique in this plasmid so that double digestion with *Eco*RI and *Bam*HI enables to check the insert fragment after construction of a derivative of pMGJK72.
(TIF)

S4 Fig Growth curves of *E. faecium* KUHS13 isogenic mutant derivatives in BHI broth. The overnight cultures of the *E. faecium* KUHS13 strains, wild type (KUHS13), *∆orf3*, *∆orf4*, *∆orf5*, *∆orf6*, *∆orf7*, *∆orf8*, *∆orf9*, *∆orf10*, *∆orf12* or *∆orf7/∆orf8* was inoculated into fresh BHI broth at a 10-fold dilution, followed by incubation at 37°C for 1 h. The preculture was further inoculated into fresh BHI broth at a 10-fold dilution, followed by incubation at 37°C with monitoring of turbidity every 10 min during the incubation period. Constructed mutants show no difference in growth in BHI compared with wild type. Data are presented in triplicates for each mutant.
(TIF)

S5 Fig Plasmid structure of pMGJK53. Plasmid map of constructed plasmid vector for promoter assay, pMGJK53. Multiple cloning site (MCS) filled with super-folder *gfp* gene (*sfgfp*) is designed for Golden Gate cloning where *Bsa*I digestion excises *sfgfp* gene out and generates sticky end with AGTC and GATT. The *lacI* gene is expressed under the control of constitutive promoter P*lep* to repress P*lac* promoter. Expression of the cloned gene of interest is induced by IPTG.
(TIF)

S6 Fig IPTG-induced expression of cloned gene in the pMGJK53 plasmid vector. Proof of concept ectopic IPTG-induction system of pMGJK53. The overnight cultures of the *E. faecium* KUHS13 strains, wild type (KUHS13) with or without pMGJK53::*luc* was inoculated into fresh BHI broth at a 10-fold dilution, followed by incubation at 37°C with 10 µg/ml D-luciferin in the absence or the presence of IPTG at various concentrations. During the incubation period, turbidity and luminescence, which is an indicator of luciferase (*luc*) gene expression, were monitored every 10 min. Expression of *luc* gene was induced in a dependent manner of IPTG concentration without significant effect on growth curve. Data are presented in triplicates for each mutant.
(TIF)

S7 Fig Effect of the *tra* genes overexpression on the conjugative transfer of pELF2. Conjugation efficiency of the pELF2 plasmid during the overexpression of individual *tra* genes in the KUHS13 host with wild-type pELF2. Donor strain KUHS13 (harboring the overexpression vector) and recipient strain BM4105RF were mixed and incubated under filter (top panel) or broth (bottom panel) conditions. For values below the detection limit, the limit point is plotted as a proxy (purple area). Data represent the means and error bars from three independent replicates. The parental strain (KUHS13) and empty vector controls for these experiments are presented in Fig 2B.
(TIF)

S8 Fig Plasmid structure of pMGJK55. Plasmid map of constructed plasmid vector for promoter assay, pMGJK55. Form pMGJK53::*luc*, *lacI* gene and promoter to control *luc* were removed in pMGJK55. Multiple cloning site (MCS) is placed upstream of *luc* gene and designed for Golden Gate cloning where *Bsa*I digestion which generates sticky end with TCAA and AGTC.
(TIF)

S9 Fig Statistic evaluation of co-occurrence between the *tra* operon-encoded genes or pELF core genes and pELF_hp or other plasmid *rep* genes. Statistical evaluation of co-occurrence between *tra* or pELF core genes and *rep* genes. Rows and columns denote KUHS13 core genes (B) and replicons/markers (A), respectively. Cell color represents the conditional frequency (*P*(B = 1∣A = 1)), the fraction of (A)-positive genomes carrying gene (B). The “Overall” column indicates background frequency (*P*(B = 1)) across all genomes. Column labels show the number of (A)-positive genomes (*n*). Asterisks indicate significant enrichment of (B) in (A)-positive versus (A)-negative genomes (one-sided Fisher’s exact test with Benjamini–Hochberg FDR correction). Asterisks are shown only for pairs meeting the effect size threshold (Δ = *P*(B = 1∣A = 1)−*P*(B = 1)≥0.20). Significance: (*) *q* < 0.05, (**) *q* < 0.01, (***) *q* < 0.001.
(TIF)

S10 Fig Genetic characterizations of pELF-like plasmid contigs. The NJ tree, constructed from a pairwise Mash distance matrix including 109 pELF-like plasmid contigs extracted from the complete genomes is mapped with various features. Pseudo-support values (%) were calculated as the clade recovery rate across 70 NJ trees (B = 70) reconstructed from 80% taxon subsamples; light dots represent 50–69% support, while solid dots represent ≥70% support (Scale bar: 0.03). Annotations for species, and isolation metadata (Geography, Year, and Human source) were retrieved from the metadata associated with each genomic sequence record. Clonal complex (CC) designations were obtained through analysis via pubMLST. The presence of *tra* operon-encoded genes (*orf1–12*) and other pELF-core genes (N = 36) was detected using BLASTn. For the detection of core genes excluding the tra genes, the number of hits relative to the total number of core genes was scored and displayed as “core gene frequency.” The closed or open dots indicate pELF2 or selected pELF-like plasmid contigs analyzed in Fig 5.
(TIF)

S11 Fig Comparison of genetic organizations for minimal conjugation genes from various bacterial species. Each horizontal track represents one complete MGE contig, and arrows indicate CDS features. Left panels represent plasmids or ICE names and their host species. Genetic annotations are retrieved from NCBI database; pELF2: NZ_AP022343.1; pCW3: NC_010937.1; SAP1: AP005645.1; pSN22: NC_001425.2; SLP2: NC_004933.1; pRAW: HG917973; ICEA: FP671138.1; PAPI-1: AY273869.1; pT33-3: NZ_OX281346.1. The VirB4 and VirD4 genes in PAPI-1 or pT33-3 are encoded at distant genetic loci. CDSs for homologue of *virD4*, *virB4*, *virB6* and *virB8* (including putative analogues) are shown in orange, blue, green or yellow, respectively.
(TIF)

S12 Fig Distinct pore size of hexamer ring complex composed of VirD4-type ATPase homologues from linear or circular plasmid. Hexamer structure modeling of VirD4/FtsK-type ATPase from SLP2 (NC_004933; **A**), pIP501 (AJ505823.1; **B**) and pCW3 (ABC96297.1; **C**) by AlphaFold 3. Side and top view with minimum diameter of the ring pore of the hexamer are shown.
(TIF)

S1 Table Top 20 up-regulated genes in the stationary phase compared to exponential phase. (XLSX)

S2 Table Top 20 down-regulated genes in the stationary phase compared to exponential phase. (XLSX)

S3 Table Statistical analysis of conjugation frequencies for pELF2 deletion mutants (Fig 2A), complementation strains (Fig 2B), and overexpression strains (S7 Fig) compared with the respective wild-type references. (XLSX)

S4 Table Genome list used in Fig 5A. (XLSX)

S5 Table Core genes list for pELF-like plasmids. (XLSX)

S6 Table Bacterial strains used in this study. (XLSX)

S7 Table Plasmids used in this study. (XLSX)

S8 Table Oligonucleotides used in this study. (XLSX)
