# Supplementary material for: Identification of essential genes for conjugative transfer in antimicrobial resistance-associated pELF-type linear plasmids of opportunistic pathogen Enterococcus faecium
Source: PLoS Pathog. 2026 Aug 12;22(8):e1013937. doi: 10.1371/journal.ppat.1013937 (PMC13492992; doi:10.1371/journal.ppat.1013937)
Supplement: Supplementary file 1 [file ppat.1013937.s021.pdf]

In this manuscript, Kurushima *et al.* identify key genetic determinants involved in the transfer of the linear conjugative plasmid pELF2 in *Enterococcus faecium*.
The factors mediating the transfer of pELF-like plasmids remain poorly characterized. Through transcriptomic analysis, the authors show that the expression levels of plasmid-encoded genes do not significantly change in the presence of a recipient strain. This observation suggests that, unlike pheromone-responsive systems described in other enterococci, the transfer of pELF2 may not rely on recipient-induced transcriptional activation. Rather, the conjugation machinery encoded by the trapELF operon appears to be constitutively expressed under the tested conditions, raising interesting questions regarding the regulatory mechanisms governing its activity and timing. They focus on a set of genes organized in a putative operon, which they rename as tra_{pELF} genes, and hypothesize that this cluster may correspond to a novel conjugation module. Systematic single-gene deletions identify two genes, *traC* and *traD*, as essential for conjugative transfer, suggesting that they may form part of the transfer channel of this conjugative element.
This study is of particular interest because conjugation in Gram-positive bacteria is far less studied than in Gram-negative organisms, and even less so in the context of linear plasmids. In addition, the authors developed new genetic tools for *E. faecium*, a species that remains challenging to manipulate genetically. These tools will undoubtedly be valuable for the community.

Below are several points that would benefit from clarification or revision.

Major and specific comments
1- The terminology “conjugative plasmid” would be more appropriate than “transconjugative plasmid,” in line with the terminology commonly used in the conjugation field
Similarly, “conjugation-associated genes” would be preferable to “transconjugation-associated genes” to maintain consistency with the established nomenclature in the field.

2- Line 66: The abbreviation ARGs refers to antibiotic resistance genes, which is conceptually distinct from “horizontal gene transfer of AMR genes” mentioned earlier. This should be clarified to avoid confusion.

3- Lines 141–142 and Figure 2: Based on Figure 2A, the transfer frequency appears to be approximately 1×10^{-2} in filter mating and 2.5×10^{-5} in broth mating. However, the text reports 1×10^{-3} and 2.5×10^{-6}, respectively. Could the authors clarify whether this discrepancy reflects a normalization difference or a typographical error?

4- Figure 2: The *traI* mutant appears to show a detectable phenotype in broth mating, possibly even more pronounced than the *traH* mutant (Fig. 2B). The statement in lines 166-167 that “No involvement of *traE*, *traF*, *traI*, or *traK* genes in the conjugative transfer of pELF2 was detected, at least under the tested conditions” may therefore be somewhat categorical. It might be preferable to moderate this conclusion or provide statistical support clarifying the absence of a significant effect.

5- Figure 4B: What is the activity of the *tra1-3* promoters in the absence of pELF2? Including this control would help determine whether plasmid-encoded factors contribute to promoter activation.

In addition, there appears to be a peak of expression around 360 min. Considering that RNA-seq data (Fig. 1C) suggest differential expression patterns depending on growth phase, have the authors tested whether conjugation frequency differs when donor cells are used from stationary phase compared to exponential phase? This could provide further insight into the regulation of transfer.

6- Figure 4D: Although mentioned in the legend, this panel does not appear to be clearly presented or described in the Results section. Please clarify and ensure that the corresponding data are properly introduced and discussed in the main text.

7- Figure 5A: What does the orange-highlighted region between TraC and TraD represent? From the Discussion, it seems to correspond to a predicted interaction interface. This should be explicitly described in the figure legend for clarity.

8- Figure 5C: The microscopy images show the localization of TraC and TraD. However, only representative images are presented. A quantitative localization analysis would be necessary to support the conclusion stated in lines 206-207: "TraC localizes to the cytoplasmic membrane and that TraD assembles into discrete foci in a traC-dependent manner". Image analysis software such as MicrobeJ or similar tools would allow quantification across a large population (e.g., >1000 cells), enabling evaluation of global fluorescence distribution or foci formation. As acknowledged in the Discussion (lines 291–295), fluorescence appears heterogeneous across cells. Therefore, it would be advisable to temper the conclusions or to provide quantitative support for the proposed localization pattern.

Minor comments

- Table 1: "strat" should be corrected to "start".
- Line 130: The text states that the tra operon contains 11 CDSs. However, based on Table 1, the operon comprises 12 CDSs, including *traD*, which is a homolog of a T4CP/FtsK-type ATPase, while the remaining 11 are mostly of unknown function. This should be corrected for accuracy.
- Line 177 - Figure 4A: When referring to fragments Ptra1, Ptra2, and Ptra3, it would improve readability to specify "pink," "blue," and "yellow," respectively, as shown in the figure.
- Figure 5C: "mScrlet-I" should be corrected to "mScarlet-I."
- Line 226: The term trapELF has already been defined in line 118. Please revise the wording to avoid redundancy. Additionally, lines 226-228 appear to contain an incomplete sentence and would benefit from rephrasing for clarity.
