# Supplementary material for: Identification of essential genes for conjugative transfer in antimicrobial resistance-associated pELF-type linear plasmids of opportunistic pathogen Enterococcus faecium
Source: PLoS Pathog. 2026 Aug 12;22(8):e1013937. doi: 10.1371/journal.ppat.1013937 (PMC13492992; doi:10.1371/journal.ppat.1013937)
Supplement: Supplementary file 2 [file ppat.1013937.s023.pdf]

The authors have adequately addressed my previous comments and revised the manuscript accordingly.

I only have a few minor points that should be corrected before the manuscript can be accepted for publication.

Figure 2A. The authors state that deletion of *orf9* resulted in a slight reduction in transfer efficiency. However, according to Figure 2A, the deletion of *orf6* or *orf12* appears to have a similar effect on transfer efficiency as the deletion of *orf9*. Did the authors perform any statistical analyses to support the conclusion that these differences are significant? This comment also applies to the other graphs presented in the manuscript.

Consequently, the interpretation of the deletion and complementation effects in **Figure 2B** should be moderated (lines 194-195), as the observed effect does not appear to be substantially different from that observed for the *orf6* and *orf12* deletion mutants. The same consideration applies to all statements throughout the manuscript that specifically emphasize an effect of *orf9* (e.g., lines 201 and 319-320).

Figure 1C. The last gene of the *tra* operon should be labeled ***orf11*** rather than ***orf12***. Otherwise, two genes are designated as *orf12* in the figure.

Line 189. The sentence "phenotype in the Δ*orf3* and Δ*orf4* strains" should also include the *orf7* mutant. I suggest replacing it with: "phenotype in the Δorf3, Δorf4, and Δorf7 strains".
