# Supplementary material for: Identification of essential genes for conjugative transfer in antimicrobial resistance-associated pELF-type linear plasmids of opportunistic pathogen Enterococcus faecium
Source: PLoS Pathog. 2026 Aug 12;22(8):e1013937. doi: 10.1371/journal.ppat.1013937 (PMC13492992; doi:10.1371/journal.ppat.1013937)
Supplement: Supplementary file 3 [file ppat.1013937.s024.docx]

**Manuscript Number:** PPATHOGENS-D-26-00224

**Title:** Identification of the essential conjugation machinery in antimicrobial resistance-associated pELF-type linear plasmids of opportunistic pathogen *Enterococcus faecium*

Dear Editors and Reviewers,

We would like to thank the reviewers for their constructive critiques and for recognizing the medical significance of our study and the novelty of the minimized conjugation machinery. We have addressed all the concerns raised, performing several additional experiments and expanding our genomic analyses. We believe these changes have significantly strengthened the manuscript.

1. Refocused Narrative and Moderated Claims: Following the critique regarding the definition of a "novel system," we have revised the title and refocused the manuscript on the identification and characterization of the essential machinery within the pELF-type plasmid family. We now describe it as a distinct member of the minimized conjugation systems.
2. Identification of a Third Essential Gene (*orf7*/*traG*): As anticipated by Reviewer #1, we successfully constructed a deletion mutant for *orf7* (formerly *traG*). Our data confirms that *orf7* is indispensable for transfer, identifying it as a functional analog of VirB6.
3. Restructuring of Structural Modeling Data: Structural insights provided by AlphaFold3 have been moved from the Discussion to the Results section to better integrate our computational and experimental findings, as suggested by Reviewer #2.
4. Quantitative Genomic Analysis: We significantly expanded our genomic screening to include 9,174 genomes, applying rigorous statistical enrichment analysis to confirm the conservation of the *tra*_pELF_ cluster. The complete list of genomes and genes is provided in new Supplementary Tables.
5. Refined Imaging and Localization Study: While we confirmed the localization of TraC in the wild-type background, we have moderated our claims regarding assembly hierarchy by removing inconclusive data from deletion backgrounds, as suggested by Reviewer #1.
6. Extensive Language and Consistency Review: The entire manuscript has undergone professional English editing to ensure linguistic precision and resolve any typographical errors noted during the initial review.

**Response to Summary comments**

Reviewer #1: This manuscript reports on the interesting characterization of a tra region of the linear conjugative plasmid pELF2, found mainly in Enterococcus faecium. These linear plasmids are medically important in their common roles as disseminators of antibiotic resistance, most notably, to vancomycin. Despite their generally high prevalence and transmission rates, little is known of how linear plasmids conjugatively transfer. In the present study, the authors identify a putative tra operon composed of 12 or so genes, and they go on to show that deletions of two, traC and traD, abolish pELF2 transfer. TraD is an FtsK/VirD4-type ATPase, a highly conserved subunit of all conjugation systems, which are a major subfamily of the type IV secretion systems (T4SSs). TraC is a likely VirB4 ATPase ortholog, although the authors do not name it as such. Interestingly, deletions of 5 other tra genes had no or a very modest effect on plasmid transfer. Deletions of 5 other tra genes could not be generated, which for traG was unfortunate because this is most likely a member of the polytopic VirB6-like subunits that are also highly-conserved among all conjugation systems. Even so, the finding that this linear plasmid requires VirB4- and VirD4-like subunits, and does not code for other clear T4SS components possibly besides the VirB6 subunit, distinguishes this tra system from most others described to date. In fact, several conjugation systems have been reported to transfer circular or linear plasmids/chromosomal fragments via systems composed only of VirB4, VirD4 and one or more integral membrane protein – interestingly, these are found in other G+ species, including Streptomyces and Mycobacteria, as well as Mycoplasma and archaeal Thermococcus. This makes pELF2 tra system highly interesting as a genetically tractable model for a growing group of unusually minimized conjugation machines that have evolved to traffic linear or circular DNA substrates in G+s and some other species. That pELF2-like plasmids are responsible for dissemination of AB resistance among Enterococci makes them medically important subjects for studies aimed at elucidating their unusual transmission mechanism. Overall, the studies are well-developed, with the exception that the authors could not report effects of deleting several tra genes. The findings should attract a broad interest among readership interested in mobile elements, AB resistance transmission, and genome evolution.

**Response:** We are grateful for the positive assessment regarding the novelty and medical relevance of the pELF2 transfer system. As noted, the lack of data on *traG* was a limitation in our original submission. In this revision, we have successfully generated a *traG* (now designated as *orf7*) deletion mutant. Our experiments confirm that *traG* is indeed essential for transfer, identifying it as the third core subunit of this minimized apparatus alongside *traC* and *traD*. We believe this finding strongly supports the "minimized conjugation machine" model suggested by the reviewer.

Reviewer #2: In their manuscript entitled "A Novel Conjugation System in AMR-associated pELF-type Linear Plasmids of Enterococcus faecium", Kurushima ete al. used a combination of RNA-Seq, molecular constructions, confocal analysis, phylogenetic representations and in silico folding techniques to investigate the conjugative transfer machinery of a van-carrying linear plasmid of E. faecium. They could detect two genes essential for plasmid transfer and delineate the way they interact within the bacterial cell. They finally suggest that they are facing a novel conjugative system and propose a mechanistic working model.

Unfortunately, I think that the extensive work presented here fails to reach the proposed objective, i.e. describing a "novel conjugation system". The authors inferred a transfer operon of their linear plasmid based solely on the distant homology of one protein to coupling proteins of T4SS conjugative machinery and co-expression in RNA-seq experiments. Although they show that three of the 12 genes from the operon are indeed involved in transfer (with two essential), four seems not to be, and no information could be provided for the remaining six. The requirement of other proteins from outside the operon was also not tested. In the present state of the study, the conjugation system of pELF-type plasmids is still far from resolved and may actually not be novel (see below).

**Response:** We appreciate the rigorous evaluation of our manuscript. To address the concern that our initial findings were "preliminary," we have significantly strengthened the study in three key areas. First, we successfully completed the deletion analysis of traG, identifying an additional essential factor for transfer. Second, we performed a systematic structural analysis of all 12 ORFs using AlphaFold3 and HMM-based comparisons to better infer their functions and evolutionary origins. Third, we expanded our genomic analysis to 9,174 genomes, demonstrating that this tra cluster is a highly conserved synteny block across the pELF-type plasmid family. Furthermore, following your suggestion, we have moderated our claims of "novelty" and now describe the pELF2 machinery as a distinct member of the growing family of minimized conjugation systems.

Reviewer #3: In this manuscript, Kurushima et al. identify key genetic determinants involved in the transfer of the linear conjugative plasmid pELF2 in Enterococcus faecium. The factors mediating the transfer of pELF-like plasmids remain poorly characterized. Through transcriptomic analysis, the authors show that the expression levels of plasmid-encoded genes do not significantly change in the presence of a recipient strain. This observation suggests that, unlike pheromone-responsive systems described in other enterococci, the transfer of pELF2 may not rely on recipient-induced transcriptional activation. Rather, the conjugation machinery encoded by the trapELF operon appears to be constitutively expressed under the tested conditions, raising interesting questions regarding the regulatory mechanisms governing its activity and timing. They focus on a set of genes organized in a putative operon, which they rename as trapELF genes, and hypothesize that this cluster may correspond to a novel conjugation module. Systematic single-gene deletions identify two genes, traC and traD, as essential for conjugative transfer, suggesting that they may form part of the transfer channel of this conjugative element. This study is of particular interest because conjugation in Gram-positive bacteria is far less studied than in Gram-negative organisms, and even less so in the context of linear plasmids. In addition, the authors developed new genetic tools for E. faecium, a species that remains challenging to manipulate genetically. These tools will undoubtedly be valuable for the community.

**Response:** We thank Reviewer #3 for the positive and encouraging assessment of our work. We are particularly gratified that the reviewer recognized the significance of our findings regarding the constitutive expression of the *tra*_pELF_ operon, which stands in contrast to the well-known pheromone-responsive systems in enterococci. We also appreciate the acknowledgment of the genetic tools we developed for *E. faecium*; we share the hope that these will be a valuable resource for the research community. We have addressed the reviewer's specific comments and suggestions in this revised manuscript.

**Point-by-point response (Major comments)**

Reviewer #1: 1. L. 101. Fig. 1. This outcome(s) of the transcriptome analysis was only cursorily discussed – only limited changes in transcriptional profiles were observed and there was some evidence for an operon-like structure to the tra region. Yet, there are several regions/genes that appear to be highly differentially expressed in one or more growth conditions relative to other growth conditions. The authors should consider expanding briefly on some of these findings and perhaps including a suppl table identifying differentially or highly expressed genes and describing any known or predicted functions.

**Response:** We appreciate this constructive suggestion. As suggested, we have conducted a more comprehensive analysis of the transcriptomic data to better characterize the expression profiles under different growth conditions. To visualize these changes, we generated volcano plots comparing the exponential and stationary phases (new Supplementary Figure S2)

Our analysis revealed significant differential expression among several genes on pELF2; specifically, we identified numerous genes that were significantly upregulated or downregulated during the transition between growth phases. We have now included a list of the top 20 differentially expressed genes (DEGs) in new Supplementary Table S2 and S3, along with their predicted or known functional annotations. These findings provide a more detailed landscape of how the pELF2-encoded genes, including the *tra* region, respond to the host’s physiological state. We have described this in the Results section. (Lines 130–142)

2. Construction and phenotypic analyses of a traG mutation (a probable VirB6 ortholog that is essential for transfer) is likely to expand the number of required subunits of this system to three.

**Response:** We are pleased to report that, as suggested, we have successfully generated the traG (now designated as *orf7*) deletion mutant, which previously presented technical difficulties. As the reviewer correctly anticipated, our results confirm that *orf7* is indeed essential for the conjugative transfer of pELF2.

To ensure the rigor of this finding, we also addressed the potential polar effects arising from the sequence overlap between *orf12* and *orf7*. We constructed single and double deletion mutants, along with comprehensive complementation clones. Our data show that while *orf7* is indispensable, *orf12* is not required for transfer. We have updated Figure 2 and the Results section (Lines 168–205) to include these new findings, which identify *orf7* as the third essential subunit of this minimized conjugation system. Furthermore, we have expanded the Discussion section (Lines 371–380) to address the potential role of the Orf7 product (designated as TraG) as a functional analogue of VirB6.

3. Fig. 3 data are uninformative and could either be deleted or moved to a supplemental file.

**Response:** We agree with the reviewer’s assessment. Since the over-expression of individual tra genes did not significantly alter transfer efficiency, these results provide limited mechanistic insight. Accordingly, we have moved this data to the Supplementary Information (new Supplementary Figure S7) to maintain the focus of the main manuscript on the essential transfer machinery.

4. L. 169. Fig. 4. Fig. 4B/C – Why does luciferase activity only begin to increase several hours after initiation of the incubation period and peak at 5-6 h? This seems to be a very slow induction period, especially considering that several rounds of cell division presumptively occur during this time frame.

**Response:** The delay in luciferase activity (peaking at 5–6 h) reflects a technical lag inherent to the reporter system rather than the biological timing of transcriptional initiation. The delay in luciferase activity reflects a technical lag inherent to the reporter system. Our proof-of-concept experiments using IPTG induction for p53-derived plasmids (Supplementary Figure S6) demonstrate that luciferase activity peaks approximately 2 hours after induction. Furthermore, the constitutive expression control (lacking *lacI*) and the Ptra promoter exhibit similar kinetics, suggesting that the *tra* operon is likely expressed constitutively under physiological conditions. This discrepancy is attributed to the cumulative time required for mRNA translation, protein folding, and maturation of the luciferase enzyme. We have updated the Results to clarify that this temporal gap is a characteristic of the assay. On the other hand, this discrepancy is primarily attributed to the cumulative time required for mRNA translation, protein folding, and biochemical maturation of the luciferase enzyme. Additionally, while we used an *L. lactis* codon-optimized luciferase, such optimization does not always yield ideal real-time kinetics in *E. faecium*. We have updated the Results section (Lines 219–236) to clarify that this temporal gap is a characteristic of the reporter assay, whereas the transfer assay (New Fig. 3C) provides a more sensitive measure of the earliest stages of conjugation.

5. L. 169. Fig. 4. Fig. 4D is not mentioned in the text and should be, but it seems to be showing the transfer kinetics over time – with transfer rates increasing substantially within 60-120 minutes. Accordingly, transfer is occurring well before substantial transcriptional activation of the tra region. Please describe the panel D data in the manuscript and account for the wide difference in kinetics of promoter expression and plasmid transfer.

**Response:** As noted by the reviewer, Figure 4D (now Figure 3C) shows that conjugation is initiated within 60–120 minutes, preceding the peak of reporter activity. We have added a detailed description of these data to the manuscript (Lines 219–236). This discrepancy suggests that a very low "threshold" level of Tra protein expression—below the level required for a robust luciferase signal—is sufficient to assemble functional conjugation machinery and initiate DNA transfer.

6. L. 200. And L. 290. Fig. 5. It’s important to establish that the fusion proteins are stably produced, as many such fusions are proteolytically cleaved between the protein of interest and reporter. This could be done by westerns with AB’s against the reporter, or by adding an epitope tag along with the reporter sequence. Also, the apparent dependence of TraD localization on TraC should be strengthened by quantifying the instances of TraD diffuse vs localized patterns in the absence or presence of TraC among a large number of cells. It is mentioned in the discussion that only a limited number of cells clearly exhibit localization, without some type of quantitative comparison, any suggestion that TraD punctate localization patterns depend on TraC production is premature and should be deleted. Which raises a question of whether the data in Fig. 5 should even be presented.

**Response:** As suggested, we have performed additional experiments to establish the stability of the fusion proteins and the quantitative nature of the localization patterns.

Stability of Fusion Proteins: We performed immunoblotting using an anti-RFP (mScarlet-I) antibody. In both TraC-mScarlet-I and TraD-mScarlet-I samples, we detected bands corresponding to their predicted full-length sizes, strongly suggesting that these fusion proteins are produced without significant proteolytic cleavage in *E. faecium* (new Supplementary Figure S9).

Quantitative Imaging and Data Selection: We improved our imaging protocols to sample a large number of cells. This allowed us to quantitatively confirm the localization patterns of TraC-mScarlet-I in the wild-type background. However, as noted in the previous version, the localization signal for TraD remains extremely weak, making reliable quantitative comparison between strains technically difficult.

Revised Presentation of Data: Following the reviewer’s constructive suggestion that conclusions regarding the inter-dependency of these proteins might be premature, we have deleted the localization data in the deletion backgrounds. In the revised manuscript, we present only the localization patterns in the wild-type background (formerly Fig. 5, now integrated into Figure 4D alongside the structural modeling) (Lines 252–271).

By focusing on the wild-type background, we now present these data as experimental support for the cellular presence and potential clustering of the core components (TraC and TraD), aligned with our structural predictions, rather than as a definitive study on their assembly hierarchy. We believe this focus significantly strengthens the reliability of our conclusions.

Reviewer #2: The full characterization of all genes required for transfer is necessary. It could be achieved by construction of a minimal non conjugative linear plasmid, with only genes essential for replication and partitioning into which genes are successively added until transfer is restored (starting with genes from the operon of interest), as was done successfully for the Streptomyces linear plasmid SAP1 into which only two genes appeared to be essential (10.1093/bbb/zbaf090).

The authors should also consider functional homologies between the SAP1 conjugative system and pELF.

**Response**: Thank you for pointing out this very important point. We fully agree that the experimental data we presented this time does not support our proposal of a new conjugation system. We believe that only through experiments to identify the minimal essential factors you suggested will we be justified in claiming the “identification of a new system.” This approach has already been applied to actinomycetes, and it appears feasible to examine the system in *Enterococcus* using a similar approach. However, there are several reasons why implementation is difficult at this stage. First, the minimal unit of replication for this plasmid (as well as the molecular mechanism) has not been identified, making it impossible to experimentally construct an artificial minimal plasmid. Second, as noted in this paper, the development of genetic tools for *E. faecium* is lagging, and we anticipate that significant hurdles remain for the large-scale modification or introduction of DNA regions. For these reasons, we have decided to refrain from claiming the existence of a “novel conjugation system” in this paper.

Additionaly, HMM protein or alphafold structural comparisons of all genes of the operon to known databases could help to infer their putative functions and provide a consistent mechanistic model.

**Response:** We have conducted a systematic HMM and AlphaFold3-based analysis of the 12 ORFs, as summarized in the revised Table 1. Interestingly, while these genes are indispensable for conjugation, our structural analysis revealed that they lack significant structural homology to canonical T4SS components (e.g., VirB3, VirB4, VirB6, VirB11, etc.).

For instance, although *orf7* (*traG*) is essential and contains multiple transmembrane helices typical of a membrane scaffold, AlphaFold3 failed to find a structural match with known VirB6 proteins. Similarly, the ATPases *orf3* (*traC*) and *orf4* (*traD*) show greater similarity to simple ATPase domain and FtsK-family proteins, respectively, than to classical T4SS subunits.

Instead, following suggestion from Reviewer#1, we have described these genes as "functional analogues" rather than direct orthologs as described in the discussion section (Lines 351–380).

In introduction, current state of the art about conjugative systems in general, and of other linear plasmids, is critically needed. Although studies mainly focused on Streptomyces linear plasmids, some systems have been totally decrypted including the one stated above (10.1093/bbb/zbaf090).

**Response:** We thank the reviewer for this suggestion. We have updated the Introduction to include current knowledge of conjugative transfer systems and other linear plasmids reported in various bacterial species (Lines 87–105).

The phylogenetic analysis would require more precision. I don't understand where the 675 E. faecium genomes came from. In RefSeq there is currently more than 5000 genomes available for this speices and probably still more than 2000 two or three years ago. On the other hand there is only 413 complete ones, and not 675. pELF-like plasmids were collected using the 500bp terminal region but the authors ended up with only complete plasmids. I can't believe that no pELF plasmid was present in several contigs in draft genomes.

**Response:** We take the reviewer’s point regarding the genomic sample size seriously. To provide a more comprehensive and precise evolutionary analysis, we have significantly expanded our dataset to include both complete and draft genomes.

Specifically, we analyzed a total of 9,174 genomes of *E. faecium* and *E. lactis* available in public databases. Using this expanded dataset, we mapped the presence of the pELF-specific hairpin (pELF_hp) and the 12 candidate tra genes (Fig. 5A). Our results revealed that 11 of the 12 tra genes were significantly enriched in pELF_hp-positive genomes (p < 0.05, one-tailed test; Fig S10), whereas *orf11* showed low conservation. This supports the suggestion by Reviewer #2 that *orf11* may not be a core component of the conjugation machinery. To ensure full transparency and reproducibility of this large-scale screening, we have provided the complete list of the 9,174 genomes and the core and *tra* genes as Supplementary Tables S3 and S4, respectively.

Furthermore, from this large-scale screening, we focused 109 complete pELF-like plasmid contigs (Supplementary Fig. S11). Synteny analysis of representative plasmids confirmed that these 11 tra genes are highly conserved as a stable synteny block across the pELF plasmid family (Fig. 5B). We believe this large-scale analysis addresses the reviewer’s concerns regarding the representativeness of our samples and provides a robust genomic foundation for our study.

Comments from the reviewer #3

Major and specific comments

1- The terminology “conjugative plasmid” would be more appropriate than “transconjugative plasmid,” in line with the terminology commonly used in the conjugation field Similarly, “conjugation-associated genes” would be preferable to “transconjugation-associated genes” to maintain consistency with the established nomenclature in the field.

**Response**: We appreciate the helpful suggestion. We have replaced all instances of 'transconjugative' or 'transconjugation' in the paper with 'conjugation'.”

2- Line 66: The abbreviation ARGs refers to antibiotic resistance genes, which is conceptually distinct from “horizontal gene transfer of AMR genes” mentioned earlier. This should be clarified to avoid confusion.

**Response:** We appreciate this comment. To clarify the definition of ARGs and distinguish them from the process of horizontal gene transfer, we have revised the corresponding section as follows:

“**In *E. faecium*, horizontal gene transfer of antimicrobial resistance genes (ARGs)**” (Lines 69–70)

3- Lines 141–142 and Figure 2: Based on Figure 2A, the transfer frequency appears to be approximately 1 × 10-2 in filter mating and 2.5 × 10-5 in broth mating. However, the text reports 1 × 10-3 and 2.5 × 10-6, respectively. Could the authors clarify whether this discrepancy reflects a normalization difference or a typographical error?

**Response:** We apologize for the discrepancy. The values in the text were incorrect and have been updated to match the data presented in the figure as follows:

“**The wild-type parent strain KUHS13 exhibited transfer efficiencies of approximately 1.0 × 10−2 and 1.0 × 10−5 in filter and broth mating, respectively.**” (Lines 175–177)

4- Figure 2: The traI mutant appears to show a detectable phenotype in broth mating, possibly even more pronounced than the traH mutant (Fig. 2B). The statement in lines 166-167 that “No involvement of traE, traF, traI, or traK genes in the conjugative transfer of pELF2 was detected, at least under the tested conditions” may therefore be somewhat categorical. It might be preferable to moderate this conclusion or provide statistical support clarifying the absence of a significant effect.

**Response:** We thank the reviewer for this insightful observation. We have re-evaluated the phenotypes of *orf8* (formerly *traH*) and *orf9* (formerly *traI*). Although the reduction in efficiency is minor, we have moderated our conclusion in the revised manuscript to state that these genes may play an auxiliary role in enhancing conjugation efficiency, rather than being entirely non-involved, particularly in broth mating conditions. (Lines 178–181)

5- Figure 4B: What is the activity of the tra1-3 promoters in the absence of pELF2? Including this control would help determine whether plasmid-encoded factors contribute to promoter activation. In addition, there appears to be a peak of expression around 360 min. Considering that RNA-seq data (Fig. 1C) suggest differential expression patterns depending on growth phase, have the authors tested whether conjugation frequency differs when donor cells are used from stationary phase compared to exponential phase? This could provide further insight into the regulation of transfer.

**Response:** Response: We thank the reviewer for these insightful suggestions. We agree that analyzing reporter signals in a pELF2-absent background would be highly informative for identifying potential plasmid-encoded regulatory factors.

However, we currently do not possess an E. faecium strain devoid of pELF-type plasmids, and constructing such a strain from our clinical isolates was not feasible within the timeframe of this revision. Nevertheless, we observed significant bioluminescence during the construction and maintenance of these reporter plasmids in E. coli. This observation strongly suggests that the basal activity of the identified promoters relies on transcriptional machinery conserved across a broad range of bacterial species, rather than being strictly dependent on plasmid-specific activators.

Regarding the growth phase-dependent transfer, we observed a peak in transfer frequency during the later stages of growth (Fig 3C). However, our kinetic data show that transconjugants are generated immediately after mixing, which indicates that the transfer machinery is pre-assembled even in the early stages. The apparent fluctuation during the exponential phase (30–120 min) likely reflects a transient "dilution effect," where rapid cell division temporarily outpaces the assembly of the conjugation apparatus. This supports our conclusion that the system is constitutively expressed rather than being specifically induced at a particular growth point. We have added this interpretation to the Results section (Lines 227–236) to provide further insight into the regulation of transfer.

6- Figure 4D: Although mentioned in the legend, this panel does not appear to be clearly presented or described in the Results section. Please clarify and ensure that the corresponding data are properly introduced and discussed in the main text.

**Response:** We apologize for the omission of the description for Figure 4D (now Figure 4C) in the Results section. We have now added the corresponding explanation to properly introduce and discuss these data in the main text.

7- Figure 5A: What does the orange-highlighted region between TraC and TraD represent? From the Discussion, it seems to correspond to a predicted interaction interface. This should be explicitly described in the figure legend for clarity.

**Response:** We appreciate the suggestion. We have updated the figure legend to explicitly describe this feature as follows:

“The orange link indicates a predicted protein-protein interaction interface.” (Line 741–742)

8- Figure 5C: The microscopy images show the localization of TraC and TraD. However, only representative images are presented. A quantitative localization analysis would be necessary to support the conclusion stated in lines 206-207: “TraC localizes to the cytoplasmic membrane and that TraD assembles into discrete foci in a traC-dependent manner”. Image analysis software such as MicrobeJ or similar tools would allow quantification across a large population (e.g., >1000 cells), enabling evaluation of global fluorescence distribution or foci formation. As acknowledged in the Discussion (lines 291–295), fluorescence appears heterogeneous across cells. Therefore, it would be advisable to temper the conclusions or to provide quantitative support for the proposed localization pattern.

**Response:** We have improved our imaging protocol to enable larger sampling sizes and more robust analysis. This allowed us to quantitatively confirm the localization pattern of TraC-mScarlet-I in the wild-type background. However, as noted in the previous version, the localization signal for TraD remains extremely weak, making reliable quantitative comparisons between different genetic backgrounds technically challenging. Following the constructive suggestion from Reviewer #1 to avoid premature conclusions regarding the hierarchy of assembly, we have removed the localization data in the deletion backgrounds. In the revised manuscript, we now present the localization patterns only in the wild-type background (integrated into Figure 4E). By focusing on the wild-type background, we present these data as experimental support for the cellular presence and potential clustering of core components (TraC and TraD) consistent with our structural modeling, rather than a definitive study on assembly hierarchy. We believe this refined focus significantly strengthens the reliability of our conclusions.

**Point-by-point response (Minor comments)**

Reviewer #1: 1. L. 22. Transconjugative/transconjugation – this word is not generally used to refer to conjugation, suggest substituting conjugation for transconjugative or transconjugation throughout.

**Response**: We appreciate the helpful suggestion. We have replaced all instances of 'transconjugative' or 'transconjugation' in the paper with 'conjugation'.”

2. L. 51. Abstract. This overstates the findings; in fact, the authors identified VirB4 and FtsK/VirD4-like subunits and a likely VirB6 ortholog, and demonstrated that the first two are essential. This aligns the pELF2 system with other systems described in species mentioned above that appear to rely on only two or three of the 6 or more components characteristically found among other conjugation systems in G- and G+ species.

**Response**: Thank you for pointing that out. We have corrected the relevant section as follows:

“**By developing a genetic manipulation framework for *E. faecium*, a comprehensive mutational analysis of the *tra* region was performed and the following three essential genes were identified: *orf3_traC* (a putative VirB4 analog), *orf4_traD* (a VirD4-like coupling protein), and *orf7_traG* (a putative VirB6 analog). These genes are indispensable for conjugative transfer.**” (Lines 30–34)

3. L. 52. In fact, the authors have not confirmed the absence of the pELF2 genes among other conjugation systems, as this would entail an exhaustive analysis of the entire collection of tra systems. What the authors have shown is that the pELF2 tra genes are widely conserved among other pELF-type plasmids.

**Response**: We thank the reviewer for this important distinction. We agree that our analysis focused on the prevalence of these genes within the pELF-type plasmid family rather than an exhaustive comparison across all known conjugation systems. Accordingly, we have revised the text to accurately reflect the scope of our findings:

“**We confirmed that these genes are highly conserved among pELF-type plasmid sequences deposited in public database.**” (Lines 36­–38)

4. L. 54. As this system likely resembles those previously identified in a few other G+ species, it is not unique, but rather a new member of this unusual group of conjugation systems whose architectures and mechanisms of action remain unknown.

**Response**: Thank you for pointing that out. We have corrected the relevant section by removing indicated expression as follows:

“**The study findings revealed that pELF-type plasmids utilize highly minimized conjugation machinery, which is similar to unusual systems previously identified in other gram-positive bacteria, such as Streptomyces.**” (Lines 38–40)

5. L. 119. The authors should mention that TraC is a likely VirB4-like ATPase. The evidence for this rests on the fact that, like the VirD4-like subunits, it is essential for transfer and is a member of the FtsK-like ATPases; also, it adopts a predicted homohexameric architecture and modeling suggests stacking of TraC and TraD as also predicted for the VirB4/VirD4 arrangement.

**Response:** We agree with the reviewer’s assessment. As suggested, we have expanded the Discussion section to state that TraC is likely a functional analogue of VirB4. We emphasized that this is supported by its essentiality for transfer, possessing FtsK-like ATPase family, and structural modeling which predicts a homohexameric architecture that stacks with TraD—a configuration highly reminiscent of the canonical VirB4/VirD4 arrangement. (Lines 351–370)

6. L. 224. The discussion should highlight similarities in the present findings with the features of conjugation systems identified in a few other G+ systems (Streptomyces, Mycobacteria), Mycoplasma, Thermococcus, and Pseudomonas aeruginosa (PAP1 system). In all of these systems, an Ftsk/VirD4 motor protein functionally interacts with a VirB4-like ATPase and likely one or more integral membrane proteins (possible VirB6 orthologs) to mediate transfer of linear fragments of chromosome or circular plasmids.

**Response:** In the revised Discussion section, we have added a comprehensive comparison with conjugation systems identified in other species, such as *Streptomyces*, *Mycobacteria*, *Mycoplasma*, *Thermococcus*, and *Pseudomonas aeruginosa* (PAP1 system) along with genetic organization comparison (new Supplementary figure S12). (Lines 381–403)

7. L. 310. A recent paper described the structure of the TcpA, a T4CP associated with a conjugation system in Clostridium. This structure is more reminiscent of FtsK than VirD4, leading the authors to speculate that pCW3, the plasmid that codes for TcpA, is transferred as a dsDNA substrate rather than being converted to an ssDNA intermediate via RCR. Foor a broader comparison, the authors should consider presenting this structure in the figure along with the predicted structures of TraC/D and the pIP501 homolog.

**Response:** Following the reviewer’s suggestion, we performed pore-size estimation based on structural modeling of the TcpA hexamer (Supplementary Fig S13). Our results revealed that the pore size is large, similar to those of the ATPase motors in SLP2 and TraC/D in pELF2, both of which are proposed to utilize dsDNA as a substrate. This finding provides additional evidence supporting the hypothesis that pore diameter correlates with the substrate type and further suggests that the pELF conjugation system likely facilitates the transfer of dsDNA rather than an ssDNA intermediate (Lines 420–429).

8. Fig. S1. It should be mentioned in the legend that the three lines represent growth curve replicates for the same strain – presuming that this is what’s being shown.

**Response**: Thank you for pointing that out. We have revised the figure legend by adding the relevant section as follows: “**Data points from an experiment performed in triplicate are shown (n = 3).**”

Reviewer #2: other issues:

Genes of the suspected operon should not be named until their functions are characterized. In fact, the authors showed themselves that traE, traF, traL and traK seems to not be involved in transfer. Naming them tra is therefore highly speculative and misleading. I recommend naming genes of the operon as orfX, with X being ther rank in the operon or from the start of the plasmid sequence.

On the other hand, as TraD shows domain and structural similarities with VirD4 proteins, it would preferably be named VirD4 to match current nomenclature of conjugative system (10.1093/femsre/fuaf069), or at least TraB_D4 as proposed for Streptomyces plasmids. As of traC, it could be named VirB3 according to the two transmembrane proteins and C-terminal binding region functionally similar to other virB3.

**Response:** We appreciate the reviewer’s cautious approach toward nomenclature. We agree that naming genes before functional characterization can be misleading. To address this, we have revised the manuscript as follows:

Use of "orf" for non-essential genes: For the genes within the cluster that were found to be non-essential for transfer (*orf1*, *orf2*, *orf5*, *orf6*, *orf8*, *orf9*, *orf10*, *orf11*, *orf12*). We no longer use "*tra*" for these genes to avoid speculation. Naming of essential genes (*traC*, *traD*, and *traG*): For the three genes whose essentiality was experimentally confirmed.

Regarding the reviewer’s suggestion to name TraC as VirB3: while we acknowledge the presence of transmembrane regions, our structural modeling and the Editor’s feedback strongly suggest that TraC functions as the primary ATPase motor (VirB4-analogue), which is indispensable for the conjugation of pELF-type plasmids. To maintain consistency with the Editor's comments and to emphasize its role as a motor protein rather than a smaller accessory protein like VirB3, we have chosen to retain the VirB4-analogue designation.

We believe this combined approach—using "*orf*" for uncharacterized genes and a descriptive, dual name for essential ones—provides both scientific rigor and clarity for the reader.

traK is obviously less expressed in the stationnary phase (fig 1B). What was the rationale to still include it in the investigated operon?

**Response:** We appreciate the reviewer’s astute observation regarding the expression profile of *orf11* (previous *traK*). The rationale for initially including *orf11* in the putative tra operon was based on our initial RNA-seq coverage analysis, which showed a continuous transcriptional read extending from the upstream tra genes through orf11. Because there were no clear intergenic rho-independent terminators identified between orf10 and orf11 by our initial bioinformatic screening, we conservatively defined the operon to include these downstream genes. However, as the reviewer correctly pointed out, our expanded analysis in the revised manuscript—includinggrowth phase-dependent expression (Fig. 1B) and the large-scale genomic mapping (Fig. 5)—revealed that orf11 lacks the high conservation seen in the core *tra* genes and exhibits a distinct transcriptional pattern. Based on these findings, we now agree that *orf11* likely functions outside the core conjugation machinery or is subject to distinct regulatory control. We have reflected this by re-designating it as "*orf11*" and toning down the claim that it is a constitutive member of the core *tra* operon. (Lines 303–304, 327–331)

Numerous English grammar and typo errors.

**Response:** We apologize for the suboptimal quality of the previous manuscript. The manuscript has now been thoroughly self-edited and underwent professional English proofreading to meet the required academic standards.

The purpose of testing the regulation of the operon by any of the genes therein should be indicated. Conjugation is usually regulated by specific regulatory genes not related to the pilus construction, which react to other stimuli such as bacterial stress. Results of the analysis were therefore highly expected and the figure should be put in suppl. information.

**Response:** We agree with the reviewer’s assessment that the negative results regarding the auto-regulation of the *tra* operon are highly expected and do not significantly advance the manuscript’s primary conclusions. In accordance with your suggestion, we have removed the data and discussion concerning the transcriptional regulation by the various *tra* deletion mutants. This allows us to focus more clearly on the essential transfer machinery. However, we have retained the promoter activity assays conducted in the wild-type background using various fragment lengths. We believe these data are fundamental to the study, as they experimentally define the minimal promoter region and confirm the operon-like structure of the *tra* region.

Additionally, following the identification of *traG* as an essential gene (as requested by Reviewer #1), we have prioritized the functional characterization of this subunit over the regulatory analysis, which streamlines the manuscript and strengthens its primary focus.

Fig. 6: There is some inconsistency for some genomes between panel B and panel C. For instance, NZ_CP045014 have all conserved genes but show only a very small conserved region with the sequence just above also having all conserved genes. The five follows genomes also show similar inconsistencies.

**Response:** We acknowledge the inconsistencies noted by the reviewer in the previous version of the phylogenetic and synteny analysis. To fundamentally resolve these issues and provide a more robust evolutionary framework, we have completely overhauled the entire analysis by expanding our dataset to 9,174 genomes and refining our synteny mapping criteria.

In the revised manuscript, the previous Figure 6 has been replaced by new Figure 5, which utilizes the expanded dataset and a more precise alignment algorithm. This new analysis eliminates the technical inconsistencies caused by the limited sample size and manual annotation in the original figure. By mapping the tra gene cluster across over 100 complete pELF-type contigs (Fig. 5B), we now provide a more accurate and consistent representation of the genomic conservation and synteny of these plasmids.

AlphaFold analyses deserve to be moved into the results section and have a Method section.

**Response:** Response: We agree with the reviewer’s suggestion that the structural insights provided by AlphaFold are foundational to our characterization of the TraC and TraD proteins. Following this advice, we have moved the structural modeling data from the Discussion to a dedicated subsection in the Results (Lines 239–271). Furthermore, we have added a comprehensive description of the computational pipeline—including AlphaFold3 parameters and HMM-based structural similarity searches—to the Materials and Methods section (Lines 635–642).

In the methods sections, the threshold to consider a cluster as conserved is not given, as well as parameters for the tree construction (ML tree, NJ tree, bootstraps?). Bootstrap values for the reconstructed tree should also be given.

**Response:** We apologize for the lack of technical detail regarding the phylogenetic and pangenomic analyses in the previous version. In the revised manuscript, we have significantly expanded the dataset to include 9,174 genomes and have provided all relevant parameters and thresholds in the Materials and Methods section (Lines 673–691).

Specifically:

Phylogenetic Tree Construction: For computational tractability of the large-scale dataset, we employed a distance-based approach. Pairwise genomic distances were estimated using Mash (v.2.3) with a k-mer size (k) of 21 and a sketch size (s) of 10,000. A neighbor-joining (NJ) tree was then reconstructed from the distance matrix using the ape package.

Support Values: Since standard bootstrapping is computationally prohibitive for over 9,000 genomes, we assessed node stability using a jackknife-like pseudo-support procedure (B=50 replicates, with 80% random sampling of genomes). These support values have been added to the key nodes of the reconstructed tree in Figure 5.

Conservation Thresholds: For the pangenomic analysis of pELF-type plasmids, we used Roary (v.3.13.0) with a minimum BLASTp identity threshold of 70%. This threshold was chosen to account for the potential sequence divergence of plasmid-encoded proteins across diverse clinical isolates. "Core genes" for the pELF family were defined as those with a prevalence of 85% or higher across the identified pELF-type contigs.

We believe these details provide the necessary transparency and rigor requested by the reviewer.

Minor comments:

The authors consistently use the term "transconjugation" instead of "conjugation". Although the term may be acceptable (but not recommended in my opinion) when it refers to lab-controlled experiments, it should ne be used to describe the general process of conjugation (e.g. l.98 and later).

**Response**: We appreciate the helpful suggestion. We have replaced all instances of 'transconjugative' or 'transconjugation' in the paper with 'conjugation'.”

l 85. Why interestingly ? Please remove;

**Response**: Thank you for the comment. As suggested, we have removed the word 'interestingly' from the text to maintain a more objective tone.

l.96 [...] or even the linear [...] grammatically incorrect, can't understand the meaning of the sentence.
**Response**: Thank you for the comment. As suggested, we have revised the sentence to clarify our meaning as follows:

“**known linear conjugative plasmids from other bacterial species**” (Lines 117–118)

Fig1 -> B and C panels are inverted. Please provide the reference gene(s) for the gene expression matrix.

**Response:** Thank you for pointing this out. We apologize for the incorrect labeling in Figure 1. We have swapped the labels for panels B and C and updated the figure legend accordingly. Regarding the gene expression matrix, we did not use specific reference genes for normalization. Instead, we used TPM (Transcripts Per Million) values, which normalize for gene length and total RNA coverage within each sample. This method allows for a direct comparison of transcript abundance between samples without the need for a single reference gene.

What does TPM means?

**Response:** We apologize for the lack of clarity. TPM stands for Transcripts Per Million. It is a normalization method where the sum of all TPMs in each sample is equal to 1,000,000. This represents the number of transcripts of a specific gene for every million transcripts in the sample, accounting for both gene length and sequencing depth. We have now spelled out the term and added a brief explanation of its meaning in the revised manuscript to ensure it is clear to all readers. (Lines 706–708)

Fig 1C -> typos in traF and traL

**Response:** Thank you for your careful review. following our updated nomenclature, these genes are now labeled as *orf6* (formerly *traF*) and *orf12* (formerly *traL*). We have also double-checked all other gene labels in the figure to ensure they are consistent with the current *orf* naming convention and are accurate.

Table 1: typos in footnotes and headers. Provide Accession numbers in footnotes 1.

**Response:** Thank you for the observation. We have corrected the typographical errors in the headers and footnotes of Table 1 (e.g., 'Transmembrane' and 'Roles in conjugation'). Additionally, as requested, we have provided the GenBank accession number for the pELF2 plasmid in Footnote 1.

l. 124: upstream and not downstream.

**Response:** Thank you for the correction. We have revised the sentence to correctly state that *orf12* (formerly *traL*) is encoded upstream of orf7 (formerly *traG*) as follows:

“**Notably, orf12, which is encoded immediately upstream of orf7 with a slight sequence overlap, has not been previously annotated in publicly available data (AP022343.1) as a CDS.**” （Lines 153–155）

l. 131: essentiality of what?

**Response:** We have clarified that we are investigating the essentiality of these genes in the context of conjugation as follows:

“**To investigate the essentiality of the *tra* genes, isogenic deletion mutants were constructed for every gene within the *tra* operon**” (Lines 163–164)

l.141-142: transfer efficiency values do not match what shown in figure.

**Response:** We apologize for the discrepancy. The values in the text were incorrect and have been updated to match the data presented in the figure as follows: “**The wild-type parent strain KUHS13 exhibited transfer efficiencies of approximately 1.0 × 10−2 and 1.0 × 10−5 in filter and broth mating, respectively.**” (Lines 175–177)

l.150: results for with and without empty vector are not shown in figure. Please add them.

**Response:** We apologize for the confusion. These control data are included in Figure2B , but we recognize that they were not explicitly labeled. To clarify, we have updated the figure legend to clearly state that both the parental strain (KUHS13) and the strain carrying the empty vector are shown as controls as follows: “**The parental strain without the vector (KUHS13) and the strain harboring the empty vector (vector) are shown as controls.**” (Lines 718–719)

l.162: add the control KUHS13 control in figure 3.

**Response:** Thank you for this suggestion. We would like to clarify that the data originally presented in Figure 3 (which has been moved to Supplementary Figure S7 in the revised manuscript) is part of the same dataset shown in Figure 2B. Because the parental strain (KUHS13) control is already presented in Figure 2B, we have opted to maintain consistency and avoid redundancy by referring the reader to that figure. To ensure clarity, we have updated the legend for Supplementary Figure S7 to explicitly state that the control data for these experiments are the same as those shown in Figure 2B.

Revised Supplementary Figure Legend: “**The parental strain (KUHS13) and empty vector controls for these experiments are presented in Figure 2B.**”

l.217: traF is not much conserved compared to some other genes of the operon. Why give this one as example?

**Response:** We thank the reviewer for this observation. In the revised manuscript, we have updated our genomic analysis using broader search parameters to better capture the diversity of these genes across >9,000 genomes. Under these refined conditions, we found that *orf6* (formerly *traF*) is also highly conserved among pELF-type plasmids, showing a distribution pattern similar to other orf genes in the cluster. While *orf6* does exhibit slightly lower sequence conservation compared to the core ATPases (*traC/D*), our analysis (including the updated Supplementary Fig S10) demonstrates that it is consistently present as a specific component of this synteny block. We believe that its broad prevalence across the pELF family, despite the sequence-level divergence, justifies its inclusion as a representative member of the *tra*_pELF_ operon. We have clarified this point in the revised Results section (Lines 295–308).

l.221: 4 genomes lack the trapELF operon, not 3.

**Response:** Thank you for the correction. Please note that the corresponding figure has been completely reconstructed following your later suggestions. Therefore, this specific sentence and the associated numbers have been updated/removed in the revised manuscript to ensure overall consistency.

l.286-287. We don't known wether amino-acid ranges refer to positions in the protein or a region size. PLease calrify.

**Response:** We apologize for the ambiguity. We have clarified that these ranges refer to the specific residue positions within the proteins. To provide better context, we have also included the total length of each protein (TraC: 420 aa, TraD: 458 aa) as follows:

“**Protein structure modeling using AlphaFold3 predicted a stable interaction between the C-terminal portion (approximately 300–400 a.a. out of 420 a.a.) of TraCpELF and the ATPase domain (approximately 30–320 a.a. out of 458 a.a.) of TraDpELF (Fig 3),**” (Lines 358–361)

l.325. "[..] one of the most common plasmid families" -> add "in E. faecium".

**Response:** We have added the suggested phrase to clarify that this statement refers specifically to *E. faecium* as follows:

“**The pELF-type linear plasmid family is one of the most common plasmid families in *E. faecium*,**” (Lines 448–449)

Please re-organize the method section to Match the order of the results.

**Response:** As indicated, we have completely reorganized the Materials and Methods section to follow the logical flow and order of the Results section.

Please provide the primers used to confirm your constructions by sequencing.

**Response:** We have added the primers used for the confirmation of the constructions (via sequencing) to Supplementary Table S5.

Minor comments

- Table 1: “strat” should be corrected to “start”.

**Response:** We have corrected this typographical error.

- Line 130: The text states that the tra operon contains 11 CDSs. However, based on Table 1, the operon comprises 12 CDSs, including traD, which is a homolog of a T4CP/FtsK-type ATPase, while the remaining 11 are mostly of unknown function. This should be corrected for accuracy.

**Response:** Thank you for pointing this out. We have corrected the number of CDSs in the *tra*_pELF_ operon to 12 for accuracy.

- Line 177 - Figure 4A: When referring to fragments Ptra1, Ptra2, and Ptra3, it would improve readability to specify “pink,” “blue,” and “yellow,” respectively, as shown in the figure.

**Response:** We have revised the sentence for better readability:

“**Fragments Ptra1 (pink), Ptra2 (blue), and Ptra3 (yellow) were generated by amplifying the regions from positions 71,256, 71,358, and 71,406 to 71,457 of the pELF2 plasmid, respectively (Fig 3A).**” (Lines 215–217)

- Figure 5C: “mScrlet-I” should be corrected to “mScarlet-I.”

**Response:** We have corrected this typo.

- Line 226: The term trapELF has already been defined in line 118. Please revise the wording to avoid redundancy. Additionally, lines 226-228 appear to contain an incomplete sentence and would benefit from rephrasing for clarity.

**Response:** We have removed the redundant definition and rephrased the section to ensure the sentences are complete and clear.
