# Supplementary material for: Identification of essential genes for conjugative transfer in antimicrobial resistance-associated pELF-type linear plasmids of opportunistic pathogen Enterococcus faecium
Source: PLoS Pathog. 2026 Aug 12;22(8):e1013937. doi: 10.1371/journal.ppat.1013937 (PMC13492992; doi:10.1371/journal.ppat.1013937)
Supplement: Supplementary file 4 [file ppat.1013937.s025.docx]

**Manuscript Number:** PPATHOGENS-D-26-00224R1

**Original Manuscript Title:** "Identification of the essential conjugation machinery in antimicrobial resistance-associated pELF-type linear plasmids of opportunistic pathogen *Enterococcus faecium*"

**Revised Manuscript Title:** “Identification of essential genes for conjugative transfer in antimicrobial resistance-associated pELF-type linear plasmids of opportunistic pathogen *Enterococcus faecium*”

Dear Editors and Reviewers,

We would like to thank the reviewers for their constructive critiques and for recognizing the medical significance of our study and the novelty of the minimized conjugation machinery. We have addressed all the concerns raised, performing several additional experiments and expanding our genomic analyses. We believe these changes have significantly strengthened the manuscript.

All page and line numbers cited in this letter refer to the revised manuscript with all tracked changes accepted.

**Author corrections**

Before addressing the reviewers’ comments, we must report corrections that we identified during the revision process.

First, we discovered errors in the primer set used for the construction of the pMGJK53 plasmid and have corrected the relevant entries in Supplementary Table S8. The corrected primer set extends the flanking regions used for PCR amplification of the gentamicin resistance gene from the pGPA1 plasmid. Importantly, this correction does not alter the properties of either plasmid vector or affect any biological data presented in this study. As a consequence of the corrected primer set, the fragment encoding the gentamicin resistance gene is larger than previously reported, and the overall plasmid sizes have been revised accordingly: pMGJK53 from 7,131 bp to 7,612 bp, and pMGJK55 (derived from pMGJK53) from 6,553 bp to 7,030 bp.

Second, we identified a discrepancy in the reported number of core genes. The original manuscript stated “46 genes with a prevalence of ≥85%,” which has been corrected to “45 genes with a prevalence of ≥80%” based on re-examination of the pangenomic analysis (Supplementary Table S5). We also clarified that *orf1* (72.5% prevalence) falls marginally below the core gene threshold, in addition to the previously noted *orf11* and *orf12*.

These corrections do not affect any conclusions of the study. We sincerely apologize for these errors at this advanced stage of review.

**Note on gene numbering**

In response to a comment from Reviewer #2 concerning the annotation of EfmKUHS13_31160 in AP022343.1, the genes of the *tra* operon have been renumbered according to their positional order along the plasmid. As a result, the genes previously designated *orf7*–*orf11* are now designated *orf8*–*orf12*, and the re-annotated open reading frame is now designated *orf7*. Throughout this letter, gene names follow the revised numbering; where a reviewer’s comment refers to the previous numbering, the corresponding revised name is indicated in parentheses.

| Previous numbering | Revised numbering |
| --- | --- |
| *orf1*–*orf6* | unchanged |
| *orf7* (*traG*) | *orf8* (*traG*) |
| *orf8* | *orf9* |
| *orf9* | *orf10* |
| *orf10* | *orf11* |
| *orf11* | *orf12* |
| *orf12* (re-annotated ORF) | *orf7* |

**Point-by-point response (Major comments)**

**Reviewer #2:** Title still inaccurate : still no idea if the whole essential machinery is identified, since several genes could not be deleted and that the conjugative machinery may also require genes from outside the operon.

**Response:** We agree with the reviewer’s concern. We have revised the title from “Identification of the essential conjugation machinery…” to “Identification of essential genes for conjugative transfer in antimicrobial resistance-associated pELF-type linear plasmids of opportunistic pathogen *Enterococcus faecium*,” making it clear that the study identifies individual genes rather than claiming to have defined the complete conjugation system.

l.122 and abstract: I don’t agree with the statement that the transcriptomic analysis identified the conjugation operon, since no expression change was shown with/without recipient. In this revised manuscript, I feel that the whole transcriptomic analysis is not relevant for the question, and should be reduced to the operon boundaries part. The result section should start l.142.

**Response:** We agree with the reviewer that the absence of recipient-dependent expression changes does not support the claim that transcriptomic analysis identified the conjugation operon. We have made the following changes to address this comment:

(1) The abstract has been revised to “Transcriptomic data suggested that the FtsK/VirD4-type adenosine triphosphatase is encoded within a multi-gene operon,” replacing the previous claim of identification. (Page 2, Lines 28–29)
(2) The Results section heading has been revised from “Transcriptome analysis identified a putative conjugation operon” to “Transcriptomic analysis defined the boundaries of a putative conjugation operon in the pELF-type linear plasmid.” (Page 8, Lines 123–124)
(3) The Results section now opens with a brief description of the experimental conditions, followed directly by the operon definition. The detailed growth phase-dependent expression data, which were expanded in the previous revision at the request of Reviewer #1 (1st round, Major comment 1), have been condensed to a single sentence with reference to the supplementary materials, restoring a structure consistent with the original submission:“Growth phase-dependent transcriptional changes were observed among several plasmid-encoded genes (Supplementary Fig S2, Supplementary Tables S1 and S2), whereas co-culture with recipient cells had limited effect on the transcriptional profile (Fig 1A and B).” (Page 8, Lines 131–134)

Gene numbering is a bit awkward. the authors explain that orf12 was not annotated in the reference sequence AP022343.1 of pELF2 so they had to name this gene afterward (ll.152-154). But when looking at the Genbank entry for AP022343.1 (last modification 2020), the ORF is actually annotated under the locus tag EfmKUHS13_31160! It just doesn’t start at the exact same position. So this part should be corrected, and gene names should be ordered accordingly. Similarly, locus tags in Table 1/main text/figures/tables should match those of the AP022343.1 annotation.

**Response:** We thank the reviewer for this careful observation. We have confirmed that the gene previously described as unannotated is indeed present in the AP022343.1 entry under locus tag EfmKUHS13_31160, albeit with slightly different start coordinates. The statement regarding the absence of this annotation has been corrected. Accordingly, the *orf* numbering (*orf1*–*orf12*) has been reordered to reflect the genomic position on AP022343.1, as summarized in the “Note on gene numbering” above. Furthermore, all locus tags throughout the manuscript, including Table 1, main text, and figures, have been updated to match the AP022343.1 annotation, replacing the RefSeq-derived RS-prefix locus tags. The re-annotation of this open reading frame is now indicated in a footnote to Table 1.

I still consider that since a unified nomenclature was proposed for conjugation systems (Christie et al. 2025 DOI: 10.1093/femsre/fuaf069), one should follow it as much as possible. Which means that genes should be named traD_ (=subscript), traC_, and traG_. The operon doesn’t have to be called trapELF (i.e. discussion), tra is enough, unless when compared to other conjugation systems.

**Response:** We have adopted the proposed nomenclature and thank the reviewer for directing us to this reference, which has now been added to the reference list. Because the B4 and B6 assignments for TraC and TraG rest on architectural and functional analogy rather than demonstrated sequence homology, we introduce the subscripted designations formally in the Discussion, where the supporting arguments are presented:

“In accordance with the recently proposed unified nomenclature for T4SS subunits, we propose the designations *traD*_D4_, *traC*_B4_, and *traG*_B6_[57]. We note that this assignment rests on different levels of evidence: TraD shows clear sequence-level homology to the VirD4/FtsK-type ATPase family, whereas TraC and TraG lack detectable sequence homology to VirB4 and VirB6, and their designations are based on architectural and functional analogy rather than demonstrated orthology.” (Pages 23–24, Lines 364–370)

In the Abstract, the subscripted names are used together with an explicit statement of the corresponding analogs, so that the basis of each designation is clear to the reader (Page 2, Lines 29–33). In the Results, where the designations are first assigned on the basis of the deletion phenotypes alone, the genes are referred to as *traC*, *traD*, and *traG* without subscripts (Page 14, Lines 190–192).

We have also removed the *tra*_pELF_ designation throughout the manuscript, referring to the operon simply as the *tra* operon.

Figure 3B and related text: Why cloning the reporter vector only in the strain harbouring pELF2? I think this experiment requires an additional test with the reporter vectors cloned in one of the strains lackin pELF2. Such experiment should indicate if the tra operon expression requires regulatory genes located on the plasmid itself or not.

**Response:** We thank the reviewer for this critical suggestion. Although an isogenic pELF2-cured derivative of KUHS13 is not available, we addressed this question by introducing the reporter vectors into strain MEM18094, a clinical *E. faecium* isolate belonging to the same clade A lineage but lacking pELF-type plasmids. The results demonstrated strong P*tra* promoter activity in strain MEM18094, comparable to that observed in KUHS13, indicating that P*tra* activity is not dependent on *trans*-acting factors encoded on pELF2. These data have been added as Figure 3D, and the following text has been added to the Results:

“To investigate whether P*tra* activity is affected by elements encoded on the pELF2 plasmid, we performed promoter assays in *E. faecium* MEM18094, a clade A VRE strain that lacks pELF-type plasmid (Fig 3D). Robust P*tra* promoter activity was observed in MEM18094 to an extent completely comparable to that in KUHS13, strongly suggesting that P*tra* activity is independent of the pELF-type plasmid.” (Page 16, Lines 225–229)

Figure 5 show an unexpected prevalence of traG in numerous genomes lacking pELF2. It suggests that the detection threshold is too low, or that this same protein is also present in another MGE (through genetic exchange). Please provide some genetic explanation to this observation.

**Response:** We agree with the reviewer that *traG* appears to be detected in a number of genomes lacking pELF-like plasmids. We consider this most likely to represent false-positive detection arising from the relaxed BLASTn threshold (≥70% nucleotide identity), which was intentionally set to accommodate sequence polymorphism among pELF-encoded genes. Although we have not definitively identified the specific genes responsible for these spurious hits, the detections are distributed uniformly across phylogenetic clades without bias toward particular lineages, suggesting cross-detection of a conserved chromosomal gene rather than another mobile genetic element. Notably, *traG* encodes a rhomboid-family intramembrane protease domain, and rhomboid proteases are widely distributed across bacterial chromosomes, which may account for these low-level cross-detections. Importantly, the number of such hits is limited relative to the total dataset, and the conclusion that *traG* is conserved among pELF-harboring genomes remains unaffected.

As suggested, we have noted this observation in the Results and added a genetic explanation in the Discussion:

“A small number of *traG*-like hits were also detected in genomes lacking pELF-like plasmids; a possible explanation is discussed below.” (Page 18, Lines 264–266)

“However, a small number of *traG*-like hits were detected in genomes lacking pELF-like plasmids (Fig 5A). This likely reflects cross-detection of chromosomally encoded rhomboid-family proteases, as *traG* contains a rhomboid intramembrane protease domain that is widely conserved across bacterial genomes.” (Page 20, Lines 303–306)

-l.177-180: Deletion of orf8 indeed seems to have an effect in filter mating but not in broth, and for orf9 it is not that clear compared to the other genes. Require statistical analyses here between mutants and WT (with correction for multiple tests), or shorten this part to say that there is only marginal effects for the other genes.

-l.194-196: same remark about orf8 and orf9.

*(orf8 and orf9 in this comment correspond to orf9 and orf10 under the revised numbering.)*

**Response:** This point was also raised by Reviewers 1 and 3 as Minor comments; we provide a unified response below.

We thank the reviewers for this important and shared recommendation. We have now performed statistical analyses for all deletion and complementation mutants. Conjugation frequencies were compared with the respective wild-type references using paired *t*-tests on log₁₀-transformed values, with *P*-values adjusted for multiple comparisons using the Benjamini–Hochberg method. The statistical methods have been added to Materials and Methods, and the adjusted *P*-values are now displayed in Figure 2 and its legend.

Deletion mutants of the three essential genes (*traC*, *traD*, and *traG*) consistently showed conjugation frequencies below the detection limit across all replicates and conditions, precluding formal statistical testing. Similarly, in the secondary conjugation assay (Figure 2C), all mutant strains fell below the detection limit.

For the remaining deletion mutants, including Δ*orf9* and Δ*orf10* (former Δ*orf8* and Δ*orf9*), adjusted *P*-values did not consistently fall below 0.05 across experimental systems. For example, while the Δ*orf10* deletion yielded adjusted *P*-values of 0.034 (filter) and 0.050 (broth), the corresponding vector control strains showed adjusted *P*-values of 0.086 and 0.405, respectively. Similar inconsistencies were observed for Δ*orf9*. The magnitude and direction of effects for these genes, as well as for Δ*orf6* and Δ*orf12*, were not reproducible across independent experimental systems (deletion vs. vector control). On the basis of both the statistical results and the lack of consistent effects, we conclude that these genes do not play a substantial role in conjugal transfer under the conditions tested.

We have revised the manuscript accordingly, removing all specific claims of reduced transfer for individual non-essential genes:

“Deletion of *orf3* (∆*orf3*), *orf4* (∆*orf4*), and *orf8* (∆*orf8*) completely abolished conjugative transfer. The remaining mutants (∆*orf5*, ∆*orf6*, ∆*orf7*, ∆*orf9*, ∆*orf10* and ∆*orf12*) showed no notable differences compared to the wild-type strain (KUHS13).” (Pages 13, Lines 167–170)

“Collectively, these data demonstrate that *orf3*, *orf4*, and *orf8* are essential for the conjugative transfer of pELF2. No involvement of *orf5*, *orf6*, *orf7*, *orf9*, *orf10* or *orf12* in the conjugative transfer of pELF2 was detected under the conditions tested.” (Pages 14, Lines 188–190)

“Although these genes are highly conserved within the synteny block across pELF-like plasmids, deletion mutants of *orf5*, *orf6*, *orf7*, *orf9*, *orf10* and *orf12* were obtained but did not show statistically significant effects on conjugation (Figure 2 and Supplementary Table S3).” (Page 20, Lines 306–309)

**Point-by-point response (Minor comments)**

**Reviewer #1:**

1. Fig. 1C. Why are there two orf12’s? Are these identical genes, or is the second mislabeled (should it be orf11, since that is mentioned in the text but is not labeled in the figure.

**Response:** We apologize for the error in the figure. The mislabeled gene has been corrected in Fig 1C.

2. Table 1 is not configured properly in my pdf file, but I suspect it’s ok in the submitted manuscript.

**Response:** We apologize for the poor legibility of this table. The table was prepared in a spreadsheet and inserted into the Word file in accordance with the journal formatting policy; because it is wide, it did not render well in the compiled PDF. We have reformatted Table 1 to improve readability in the revised manuscript.

3. Pg. 14. L. 177. Differences in transfer frequencies by the orf8 and orf9 (also orf6 and orf12) deletion mutant relative to the WT strain should be evaluated using statistical analyses. If the differences are statistically significant, it’s ok to mention that slight reductions were observed. If not statistically significant, it should be mentioned that any observed slight reductions were not statistically significant.

**Response:** Please see our response to the Major comment from Reviewer #2 regarding l.177–180, which addresses this shared concern.

4. Pg. 9. L. 141. Fig. S1B. Should this be Fig. S2B? If so, there needs to be an A and a B added to the left and right transcriptional profiles, respectively, in Fig. S2.

**Response:** We apologize for the error in the figure citation. The citation has been corrected, and panel labels (A) and (B) have been added to the left and right transcriptional profiles, respectively, in Supplementary Fig S2. The legend now reads:

“Volcano plot presents differential expression of pELF2-encoded genes between conditions of exponential and stationary phase (A) or with or without recipient cells (BM4105RF) (B).” (legend for Supplementary Fig S2)

5. Pg. 15. L. 194. These slight reductions (more obvious for the orf8 deletion, not at all evident for the orf9 deletion) and the putative enhancement of transfer upon complementation should be evaluated for statistical significance.

**Response:** Please see our response to the Major comment from Reviewer #2 regarding l.177–180, which addresses this shared concern. Regarding the putative enhancement upon complementation, the complemented strains did not show statistically significant recovery of conjugation frequency relative to the respective deletion mutants, and these claims have been removed from the manuscript.

6. P. 15. L. 201. Delete all references to reductions in transfer by the orf8 and orf9 deletions unless shown to be statistically significant.

**Response:** Please see our response to the Major comment from Reviewer #2 regarding l.177–180, which addresses this shared concern. All references to reductions in transfer by these deletions have been removed from the manuscript (Page 13, Lines 167–170; Page 14, Lines 188–190; Page 20, Lines 308–311).

7. P. 17. L. 252. Fig 4C. The figure shows that each TraC monomer has 2 possible TM helices, not multiple. The hexamer is predicted to have 12. It’d be more accurate to state either that the monomer has two predicted TM’s or the hexamer has 12 predicted TM’s.

**Response:** The text has been corrected as follows:

“In contrast, although TraC exhibited no significant sequence similarity to known proteins, a distinct ATPase domain was detected at its C-terminus, and two transmembrane helices were identified (Fig 4A).” (Page 17, Lines 238–239)

8. P. 18. L. 257. This line of investigation doesn’t provide any evidence supporting the proposed interaction, which could be due to the transient nature of the interaction as the authors suggest. However, the diffuse membrane distribution of TraC is most likely an artefact resulting from overproduction of the reporter-tagged TraC – in all other characterized T4SSs, the T4SSs assemble as discrete machines at the cell envelope, they are not uniformly distributed around the cell. Assuming that the distribution pattern of TraC is an overproduction artefact, or that only a very small fraction of TraC actually assembles into an active machine, it’s premature to suggest that TraD associates only transiently with TraC. Indeed, the tagged TraC does seem to form regions of brighter fluorescence at certain locations, relative to the dim but uniform membrane fluorescence. Regardless, given that this line of investigation generates no firm conclusions one way or the other, I suggest deleting it. Clearly, further work, e.g., biochemical crosslinking/affinity chromatography, is needed to test the proposed TraD/TraC docking model, which is beyond the scope of this manuscript.

**Response:** We thank the reviewer for this insightful assessment. We agree that the apparent membrane localization of TraC likely represents an overproduction artifact, and that the fluorescence microscopy data are too preliminary for definitive conclusions at this stage. Accordingly, we have removed all fluorescence imaging data and the associated discussion from the manuscript, together with the corresponding Materials and Methods section, the abstract sentence, and the description of the mScarlet-I fusion constructs.

9. Pg. 23. L. 370. I suggest that the authors run an alphafold prediction model with 5 copies of TraG. This clearly shows a transmembrane channel, which potentially could also be docked onto the TraC/D homohexameric assembly. In other T4SSs, the VirB6 homologs invariably assemble as homopentamers that associate with the VirB4 hexamer-of-dimers at the membrane. Whether the authors want to include this additional information in the manuscript is up to them, but the TraG pentameric model certainly supports a proposal that a TraC/D/G ternary complex constitutes the bulk - and perhaps the entirety – of the transfer channel in this species.

**Response:** We thank the reviewer for this valuable suggestion. Following this recommendation, we performed AlphaFold structure prediction for multimeric TraG complexes (shown below). Interestingly, our analysis suggested that TraG forms a more stable homohexamer rather than the homopentamer typically observed for VirB6 homologs in other T4SSs. Furthermore, no interactions between TraG and TraC or TraD were predicted by AlphaFold. While these observations are intriguing, we consider the evidence insufficient to propose a definitive ternary complex model at this stage, and we prefer to address this as a subject for future investigation.

10. Pg. 24. L. 380. Based on the figure (S12), the text seems to be overstating the conserved nature of these systems, e.g., all systems are assembled from homologs or orthologs of VirD4, VirB4, and VirB6-like subunits. While most appear to have these homologs, some seem to lack one or both VirB4 or VirB6-like subunits, e.g., Streptomyces systems that only have VirD4-like TraB. The only universally conserved subunit is the FtsK/D4-like ATPase.

**Response:** We agree with the reviewer that the conserved nature of these systems was overstated. As the reviewer correctly points out, the only universally conserved subunit is the FtsK/VirD4-like ATPase, while VirB4 and VirB6-like subunits are variably present. The text has been revised as follows:

“In these systems, a minimal FtsK-type motor unit, centered on a universally conserved translocator (VirD4/FtsK-like), in some cases accompanied by a partner ATPase (VirB4-like), functionally interacts with variable numbers of integral membrane proteins (putative functional counterparts of VirB6 or VirB8) to mediate the transfer of linear fragments of chromosomes, plasmids, or integrative conjugative elements.” (Page 24, Lines 374–380)

**Reviewer #2:**

**Abstract**

-l.31: correct “comprehensive” by “extensive”, since not all tra genes were mutated.

**Response:** The text has been corrected as follows:

“By developing a genetic manipulation framework for *E. faecium*, an extensive mutational analysis of the *tra* region was performed and the following three essential genes were identified” (Page 2, Lines 29–32)

-l.36: remove “of the protein fusions” and add “for the encoded proteins” at the end of sentence

**Response:** Following Reviewer #1’s comment 8, the fluorescence microscopy data have been removed in their entirety; the corresponding sentence has therefore been deleted from the abstract rather than revised.

-l.42-43: assertion too imprecise. transmission of linear plasmids is a very specific case, and do not help understanding evolution and disemination of AMR in general.

**Response:** The text has been revised as follows:

“This study provides the first molecular insights into the transmission of these clinically important linear plasmids in enterococci and lays a foundation for understanding the dissemination of resistance determinants mediated by atypical mobile genetic elements.” (Pages 2–3, Lines 39–42)

**Author summary**

-l.52: “several” essential genes.

**Response:** The text has been revised as follows:

“In this study, the several essential genes that drive the conjugative transfer of pELF-type plasmids were identified.” (Page 4, Lines 51–52)

-l.54-55: Same comment, do not agree since not all mutants were provided. Perhaps the last 3 genes are necessary too.

**Response:** The text has been revised as follows:

“The study findings reveal that these plasmids utilize a minimized set of transfer machinery, though the potential involvement of other uncharacterized genes warrants further investigation.” (Page 4, Lines 52–54)

-l.56: 9,000 “E. faecium” genomes

**Response:** The text has been revised as follows:

“By analyzing more than 9,000 *E. faecium* genomes, the study further demonstrated that this specific transfer machinery is highly conserved and serves as a hallmark of this plasmid family.” (Page 4, Lines 55–57)

-l.59: role of “these” unique

**Response:** The text has been corrected as follows:

“thereby offering crucial insights into the role of these unique mobile elements in the global spread of antibiotic resistance in hospital environments.” (Page 4, Lines 58–60)

**Introduction**

-l.105: recently discovered “in E. faecium”

-l.106: the plasmid by itself doesn’t allow the integration of MGEs. Please rephrase.

**Response:** Both points have been addressed in the same sentence, which has been corrected as follows:

“The pELF-type plasmid was recently discovered in *E. faecium* and is a large (>100 kb) linear DNA molecule that serves as a platform for the integration of multiple genetic elements.” (Page 7, Lines 105–107)

-l.117: “the genetic functions” is unclear. Please rephrase.

-l.118: remove “novel conjugation”.

**Response:** Both points have been addressed in the same sentence, which has been revised as follows:

“In this study, the conjugative transfer mechanisms of the pELF-type linear conjugative plasmid family were analyzed and genes essential for conjugative transfer of the pELF plasmid were identified.” (Page 7, Lines 117–120)

**Results**

-l.132: with “a” toxin-antitoxin system. The plasmid my carry more than one. But should not be present after correction.

**Response:** As the reviewer anticipated, this sentence is no longer present. In response to the reviewer’s major comment on the transcriptomic analysis, the detailed description of growth phase-dependent expression, including the sentence referring to the toxin–antitoxin system, has been condensed into a single sentence with reference to the supplementary materials (Page 8, Lines 131–134).

-Table 1: barely readable with the current formatting.

**Response:** We apologize for the poor legibility of this table, which was also noted by Reviewer #1. Table 1 has been reformatted to improve readability. The locus tags have also been updated to the AP022343.1 annotation, and a footnote indicating the re-annotated open reading frame has been added.

-l.163: were “tentatively” constructed for every gene

**Response:** The text has been corrected as follows:

“To investigate the essentiality of the *tra* genes, isogenic deletion mutants were tentatively constructed for every gene within the *tra* operon.” (Page 12, Lines 154–156)

-l.169: “could not be generated”

**Response:** The text has been corrected as follows:

“Deletion mutants of *orf1*, *orf2*, or *orf11* could not be generated.” (Page 13, Lines 161–162)

-l.170-173: for clarification I would suggest to rephrase these two sentences as follows: “After verification that these gene deletions did not affect bacterial growth (Fig S4), the pELF2 deletion mutant collection was evaluated as donor strain in congugative […]”

**Response:** We thank the reviewer for this suggestion, which we have adopted:

“After verification that these gene deletions did not affect bacterial growth (Supplementary Fig S4), the pELF2 deletion mutant collection was evaluated as donor strain in conjugative transfer experiments under both filter and broth conditions, using BM4105RF as the recipient strain (Fig 2A).” (Page 13, Lines 162–165)

-l.196-199: Figure 2B doesn’t show transfer efficiencies of the WT KUHS13 with + cloned genes in pMGJK53. So the sentence is not correct.

**Response:** We apologize for this inaccuracy. The sentence has been corrected to refer to Supplementary Fig S7, in which these data are presented:

“Furthermore, the ectopic overexpression of individual genes in the wild-type KUHS13 background yielded no considerable differences compared to the wild-type KUHS13 lacking an expression vector or harboring the control pMGJK53::*gfp* vector (Supplementary Fig S7 and Supplementary Table S3).” (Page 14, Lines 184–187)

-l.210-211: replace by “transcriptional start site upstream of the orf1 gene, at position 71,468 in the AP022343.1 reference sequence”.

**Response:** We thank the reviewer for this suggestion, which we have adopted:

“The operon-like expression pattern observed in the RNA-seq data suggests a transcriptional start site upstream of the *orf1* gene, at position 71,468 in the AP022343.1 reference sequence (Figs 1 and 3A).” (Page 15, Lines 197–200)

-l.215: please indicate the end position for each cloned promoter region.

**Response:** The end positions have been added as follows:

“Fragments P*tra*1 (pink), P*tra*2 (blue), and P*tra*3 (yellow) were generated by amplifying regions from positions 71,256–71,457, 71,358–71,457, and 71,406–71,457 of the pELF2 plasmid, respectively (Fig 3A).” (Page 15, Lines 202–205)

-l.262: “exhibited an unstable”

**Response:** We thank the reviewer for noting this error. Following Reviewer #1’s comment 8, the fluorescence microscopy data and the associated text have been removed in their entirety; this sentence is therefore no longer present in the manuscript.

-l.272: remove “evolutionary relationships” since it is not the purpose of the study, and it was not really investigated.

-l.274: E. faecium/lactic complex only.

**Response:** Both points have been addressed in the same sentence, which has been revised as follows:

“To elucidate the prevalence of pELF-type plasmids within *Enterococcus* species, a large-scale comparative genomic analysis was conducted using 9,174 genomes of *E. faecium*/*E. lactis* complex, encompassing all assembly levels from draft to complete (Supplementary Table S4).” (Pages 17, Lines 247–250)

-l.276: “provided”

**Response:** The text has been corrected as follows:

“The phylogenetic tree based on pairwise Mash distances provided a comprehensive overview of the genomic diversity of *E. faecium* and *E. lactis* (Fig 5A).” (Page 17, Lines 250–251)

-l.280: Phylogenetically speaking, it is not consistent to have both E. faecium and E. lactis mixed in clade B. It is likely caused by strain identification before the formal separation between the two species. When looking at some clade B E. faecium provided in suppl. Table 3, it appears that on their NCBI Assembly page, they are more related to the E. lactis type strain than E. faecium type strain. It should be stated in the text.

**Response:** We thank the reviewer for this insightful observation, with which we fully agree. The presence of *E. faecium* strains within Clade B is a phylogenetic artifact resulting from historical nomenclature used before *E. lactis* was formally separated as a distinct species, and inspection of the NCBI Assembly data confirms that these database-registered strains are more closely related to the *E. lactis* type strain. We have stated this taxonomic context explicitly in the revised text:

“This mixed composition is likely due to the historical classification of these strains before *E. lactis* was formally recognized as a distinct species, as the registered *E. faecium* strains in this clade actually share closer phylogenetic proximity to the *E. lactis* type strain.” (Page 17–18, Lines 255–257)

-Figure 5: “orf12” and not “tra12”

**Response:** We apologize for the error in the figure. The gene label has been corrected in Figure 5.

-l.287: remove “frequently”.They always co-occur with most genes of the tra operon.

**Response:** The text has been corrected as follows:

“Mapping revealed that the pELF-type plasmids were substantially enriched within Clade A1, where they co-occurred with most *tra* genes.” (Page 18, Lines 23–264)

-l.288: add “(Suppl. Fig S10)” after “the entire dataset”

**Response:** The citation has been added as follows:

“Statistical analysis further demonstrated that the presence of pELF_hp strongly correlated with the enrichment of *tra* operon-encoded genes and pELF core genes across the entire dataset (Supplementary Fig S9).” (Page 18 Lines 266–269)

-l.303-304: don’t agree. genes with best prevalence (>93%) are of unknown function in table S4.

**Response:** We agree with the reviewer and have corrected this statement:

“A total of 45 core genes defined by a prevalence of ≥80% across all pELF-type plasmids were identified, including plasmid maintenance genes and 9 of the 12 *tra* operon genes (Supplementary Table S5). While most genes in this core set were of unknown function, the inclusion of most *tra* genes underscores the stability of the conjugation system.” (Page 19, Lines 277–281)

- Figure 5b: it is not clear how “core genes” were defined

**Response:** Core genes were defined as genes present in ≥80% of all pELF-type plasmid contigs analysed. This definition has been stated explicitly in the Results (Page 19, Lines 277–279) and in the legend of Figure 5.

**Discussion**

-l.336: “inferred” instead of “speculated”.

**Response:** The text has been revised as follows:

“Promoter analysis of the region upstream of *orf1* suggested that the transcription of the putative *tra* operon is coordinated by at least a 100-bp region from the inferred transcriptional start site.” (Page 21, Lines 319–321)

-l.346: “in the future”

**Response:** The text has been revised as follows:

“Such a mechanism may be discovered in the future as research on *E. faecium* plasmids progresses.” (Pages 22, Lines 330–331)

-l.420: “is close” and not “closer”

**Response:** The text has been corrected as follows:

“Interestingly, the diameter of the ring pore in the TraC_B4_/D_D4_ complex (estimated as 23.2 Å for TraC and 18.0 Å for TraD) is close to that of the FtsK-like ATPase ring in SLP2 (14.5 Å).” (Page 26, Lines 411–413)

-l.438-440: move this part in the first section of the discussion, when discussing about conservation of the tra operon genes. It current position in the middle of molecular construction consideration is not logical.

**Response:** We agree that the previous position was not logical. This sentence has been moved to the first paragraph of the Discussion, where the conservation of the *tra* operon is discussed:

“The large-scale analysis of 9,174 genomes further revealed that the *tra* operon is remarkably conserved (11 of 12 genes detected across pELF-like plasmid-positive genomes) and markedly enriched in the human-associated high-risk Clade A1 lineage.” (Page 20, Lines 300–302)

**Methods**

-l.526-527 and 529-530: repeated sentence. Romve one.

**Response:** We apologize for this duplication. The repeated sentence has been removed:

“The obtained *sfgfp* DNA fragment was digested with *Bsm*BI and ligated into the pRecT-derived DNA fragment using T4 DNA ligase. The resulting plasmid after spectinomycin selection in *E. coli* HST08 was designated pMGJK51. Notably, the cloned *sfgfp* gene contained an internal *Bsa*I site that generated sticky ends for AGTC and GATT for the Golden Gate Assembly.” (Page 31, Lines 512–517)

-.673: Please provide the date at which genomes were retrieved.

**Response:** The retrieval date (18 March 2026) has been added to Materials and Methods (Page 37, Line 628).

-l.686: Please provide similarity/coverage thresholds for blastn hits.

**Response:** BLASTn was performed with a minimum nucleotide identity threshold of 70%. No explicit query coverage threshold was applied at the search stage; however, the default BLASTn e-value cutoff (10) effectively limits spurious short matches. The presence/absence matrix was constructed from all hits meeting the identity criterion. These thresholds have been added to Materials and Methods and to the legend of Figure 5.

- l.688: 70% amino acid or nucleotide identity?

**Response:** We have clarified that the 70% identity threshold refers to amino acid identity, as Roary uses BLASTp for gene clustering. The text has been revised accordingly:

“Pangenomic analysis of these contigs was performed using Roary, with a 70% BLASTp identity threshold to accommodate the characteristic sequence divergence of plasmid-encoded proteins.” (Page 18–19, Lines 275–277)

**Figures**

Figure 1: (A) in legend, provide the accession number of the assembly from which CDS annotation were extracted.

(B) traC is given twice on the figure
(C) orf12 is given twice.

**Response:** (A) The accession number has been added to the legend: “annotated coding sequences (CDSs; accession no. AP022343)”. (B) and (C) The duplicated labels have been corrected in the figure.

Figure 2: (B) in legend, correct “top” and “bottom” panels by “left” and “right”

(C) in legend, correct “left” and “right” panels by “top” and “bottom”

**Response:** Both errors have been corrected in the legend of Figure 2. The legend now also describes the statistical annotations added in response to the comments on statistical analysis.

Figure 4: (A) in legend, “top” and “bottom” switched. Both traC and traD contain an ATP-biding domain.

**Response:** Both errors have been corrected. The legend now reads: “Molecular structures of TraC (420 amino acids, top) and TraD (458 amino acids, bottom) proteins with conserved functional domains. TraC and TraD contain an ATP-binding domain, whereas TraC contains two transmembrane domains.”

Figure 5: (A) in legend, species names need to be in italic. How orange dots can give the jackknife value? By its size? dots are drawn only for a minimum jackknife value? Please be more precise. The scalebar is not linked to the jackknife value but to the tree branch sizes. ST identification and tra/core genes conservation procedure should be moved to methods.

Please provide similarity+coverage threshold for Blastn hits.

**Response:** We thank the reviewer for these detailed suggestions and have revised the Figure 5 legend as follows: (1) species names are now italicized; (2) the jackknife support values are now explicitly described with two thresholds (light dots ≥50%, solid dots ≥70%); (3) the scale bar is clarified as representing Mash distance; (4) methodological details for CC designation (pubMLST) and *tra*/core gene detection (BLASTn, ≥70% nucleotide identity) have been moved to Materials and Methods, with the BLASTn threshold also noted in the legend; (5) the BLASTn thresholds for synteny links in panel (B) were already provided in the original legend (length ≥ 3 kb, identity ≥ 80%).

**Supplementary Figures**

Figure S4: correct the gene names in the legend

Figure S6: remove “as expected” in the legend (l.1106)

FIgure S7: correct the gene names in the figure, and be more precise: indicate the name of the plasmid carrying the the overexpressed gene. Remove the pELF2 WT labeling from the figure and put it in the legend (l.1112).

Figure S9: (A) and (B) inverted in the legend.

**Response:** The gene names in the legends and figures of Supplementary Figs S4 and S7 have been corrected to the revised numbering. “As expected” has been removed from the legend of Supplementary Fig S6. In Supplementary Fig S7, the name of the plasmid carrying each overexpressed gene is now indicated, and the pELF2 WT labelling has been moved from the figure to the legend. The inverted panel labels in the legend of Supplementary Fig S9 have been corrected.

Figure S10: (B) is not defined.

**Response:** We apologize for this omission. The variable (B), referring to KUHS13 core genes (rows), was not explicitly defined in the legend. This has been corrected as follows:

“Rows and columns denote KUHS13 core genes (B) and replicons/markers (A), respectively.” (legend for Supplementary Fig S9)

Figure S11: The open or solid red dots are almost invisible on the figure. Please draw them bigger. And be more precise in the legend: “open and solid red dots”.

It would have been useful to mark on Figure 5A the strain from which complete pELF2 sequences were extracted. The authors set up a minimal thresold to aa 70% similarity for protein detection, but we have no idea of the actual diversity in pELF2 plasmids. A blue gradient depicting the similarity compared to the each gene from KUHS13 pELF2, instead of just presence/absence, could provide this evolutionary/epiemiological information.

**Response:** We thank the reviewer for these constructive suggestions. We have implemented all three changes: (1) the jackknife support dots in Supplementary Fig S10 have been enlarged and the legend now specifies “light dots (≥50%) and solid dots (≥70%)”; (2) strains from which complete pELF-like plasmid sequences were extracted for synteny analysis (Figure 5B) are now marked with blue diamonds on Figure 5A; (3) the binary presence/absence display for *tra* genes has been replaced with a blue gradient depicting BLASTn nucleotide identity relative to each gene from KUHS13 pELF2, providing information on evolutionary diversity across pELF-like plasmids.

Figure S12: l.1170 replace “plasmid” by “MGE”.

**Response:** The term has been replaced as requested in the legend of Supplementary Fig S11.

Table S5 and S6: missing references for strains and plasmids.

**Response:** References for the strains and plasmids have been added to Supplementary Tables S6 and S7.

**Reviewer #3:**

Figure 2A. The authors state that deletion of orf9 resulted in a slight reduction in transfer efficiency. However, according to Figure 2A, the deletion of orf6 or orf12 appears to have a similar effect on transfer efficiency as the deletion of orf9. Did the authors perform any statistical analyses to support the conclusion that these differences are significant? This comment also applies to the other graphs presented in the manuscript.

Consequently, the interpretation of the deletion and complementation effects in Figure 2B should be moderated (lines 194-195), as the observed effect does not appear to be substantially different from that observed for the orf6 and orf12 deletion mutants. The same consideration applies to all statements throughout the manuscript that specifically emphasize an effect of orf9 (e.g., lines 201 and 319-320).

*(orf9 in this comment corresponds to orf10 under the revised numbering.)*

**Response:** Please see our response to the Major comment from Reviewer #2 regarding l.177–180, which addresses this shared concern. As additionally noted by this reviewer, the effects observed for Δ*orf6* and Δ*orf12* were comparable to those for Δ*orf10* and were similarly not statistically significant. All statements emphasizing an effect of these genes have been removed (Page 13, Lines 167–170; Page 14, Lines 188–190; Page 20, Lines 308–311).

Figure 1C. The last gene of the tra operon should be labeled orf11 rather than orf12. Otherwise, two genes are designated as orf12 in the figure.

**Response:** We apologize for the error in the figure. The mislabeled gene has been corrected in Fig 1C.

Line 189. The sentence “phenotype in the ∆orf3 and ∆orf4 strains” should also include the orf7 mutant. I suggest replacing it with: “phenotype in the ∆orf3, ∆orf4, and ∆orf7 strains”.

*(orf7 in this comment corresponds to orf8 under the revised numbering.)*

**Response:** The text has been corrected as follows:

“Complementation using either pMGJK53::*orf3*, pMGJK53::*orf4*, or pMGJK53::*orf8* restored the impaired conjugation phenotype in the ∆*orf3*, ∆*orf4* or ∆*orf8* strains, respectively.” (Page 14, Lines 178–180)
