# Supplementary material for: Identification of essential genes for conjugative transfer in antimicrobial resistance-associated pELF-type linear plasmids of opportunistic pathogen Enterococcus faecium
Source: PLoS Pathog. 2026 Aug 12;22(8):e1013937. doi: 10.1371/journal.ppat.1013937 (PMC13492992; doi:10.1371/journal.ppat.1013937)
Supplement: Peer review history

# Peer review: Decision Letter 0

**DOI:** 10.1371/journal.ppat.1013937.r001

**Authors:** Michael Otto, Breck Duerkop

4 Mar 2026

A Novel Conjugation System in AMR-associated pELF-type Linear Plasmids of Enterococcus faecium.

PLOS Pathogens

Dear Dr. Kurushima,

Thank you for submitting your manuscript to PLOS Pathogens. After careful consideration, we feel that it has merit but does not fully meet PLOS Pathogens's publication criteria as it currently stands. Therefore, we invite you to submit a revised version of the manuscript that addresses the points raised during the review process.

Please submit your revised manuscript by May 03 2026 11:59PM. If you will need more time than this to complete your revisions, please reply to this message or contact the journal office at plospathogens@plos.org. When you're ready to submit your revision, log on to https://www.editorialmanager.com/ppathogens/ and select the 'Submissions Needing Revision' folder to locate your manuscript file.

Please include the following items when submitting your revised manuscript:

* A letter that responds to each point raised by the editor and reviewer(s). You should upload this letter as a separate file labeled 'Response to Reviewers'. This file does not need to include responses to any formatting updates and technical items listed in the 'Journal Requirements' section below.

* A marked-up copy of your manuscript that highlights changes made to the original version. You should upload this as a separate file labeled 'Revised Manuscript with Track Changes'.

* An unmarked version of your revised paper without tracked changes. You should upload this as a separate file labeled 'Manuscript'.

If you would like to make changes to your financial disclosure, competing interests statement, or data availability statement, please make these updates within the submission form at the time of resubmission. Guidelines for resubmitting your figure files are available below the reviewer comments at the end of this letter.

We look forward to receiving your revised manuscript.

Kind regards,

Breck A. Duerkop

Academic Editor

PLOS Pathogens

Michael Otto

Section Editor

PLOS Pathogens

Editor-in-Chief

PLOS Pathogens

Editor-in-Chief

PLOS Pathogens

orcid.org/0000-0002-7699-2064

**Additional Editor Comments:**

Thank you for submitting your paper to PLOS Pathogens. We have secured reviews from experts in the field. Despite the reviewer's agreeing that the transfer of linear plasmids that can encode antibiotic resistance is important topic of study, there were varying opinions related to the novelty and rigor of the work in proving that this is indeed a new system separate from those previously described. Specifically, reviewers point out that other systems that are similar have been reported in different G+ bacteria, Mycoplasma and an archaeal species that rely only on orthologs of VirB4, VirD4 and a polytopic VirB6-like subunit to transfer linear chromosomal fragments intercellularly. I would advise the authors pay careful attention to these comments and the requests for additional experimental evidence to support that this system truly stands out from other known systems, or whether it is related and is just the the first described for E. faecium. I believe either scenario rises to the level of consideration at PLOS Pathogens. Finally, I agree that a deeper assessment of other linear plasmid systems in bacteria and archaea be incorporated into both the introduction and discussion to better inform the reader. If you feel that you are able to address these points and others raised by the reviewers, we would look forward to receiving a revised manuscript.

**Journal Requirements:**

1) We ask that a manuscript source file is provided at Revision. Please upload your manuscript file as a .doc, .docx, .rtf or .tex. If you are providing a .tex file, please upload it under the item type u2018LaTeX Source Fileu2019 and leave your .pdf version as the item type u2018Manuscriptu2019.

2) Please upload all main figures as separate Figure files in .tif or .eps format. For more information about how to convert and format your figure files please see our guidelines:

https://journals.plos.org/plospathogens/s/figures

3) Tables should not be uploaded as individual files. Please remove these files and include the Tables in your manuscript file as editable, cell-based objects. For more information about how to format tables, see our guidelines:

https://journals.plos.org/plospathogens/s/tables

4) We have noticed that you have uploaded Supporting Information files, but you have not included a list of legends. Please add a full list of legends for your Supporting Information files after the references list.

**Reviewers' Comments:**

Reviewer's Responses to Questions

**Part I - Summary**

Please use this section to discuss strengths/weaknesses of study, novelty/significance, general execution and scholarship.

Reviewer #1: This manuscript reports on the interesting characterization of a tra region of the linear conjugative plasmid pELF2, found mainly in Enterococcus faecium. These linear plasmids are medically important in their common roles as disseminators of antibiotic resistance, most notably, to vancomycin. Despite their generally high prevalence and transmission rates, little is known of how linear plasmids conjugatively transfer. In the present study, the authors identify a putative tra operon composed of 12 or so genes, and they go on to show that deletions of two, traC and traD, abolish pELF2 transfer. TraD is an FtsK/VirD4-type ATPase, a highly conserved subunit of all conjugation systems, which are a major subfamily of the type IV secretion systems (T4SSs). TraC is a likely VirB4 ATPase ortholog, although the authors do not name it as such. Interestingly, deletions of 5 other tra genes had no or a very modest effect on plasmid transfer. Deletions of 5 other tra genes could not be generated, which for traG was unfortunate because this is most likely a member of the polytopic VirB6-like subunits that are also highly-conserved among all conjugation systems. Even so, the finding that this linear plasmid requires VirB4- and VirD4-like subunits, and does not code for other clear T4SS components possibly besides the VirB6 subunit, distinguishes this tra system from most others described to date. In fact, several conjugation systems have been reported to transfer circular or linear plasmids/chromosomal fragments via systems composed only of VirB4, VirD4 and one or more integral membrane protein – interestingly, these are found in other G+ species, including Streptomyces and Mycobacteria, as well as Mycoplasma and archaeal Thermococcus. This makes pELF2 tra system highly interesting as a genetically tractable model for a growing group of unusually minimized conjugation machines that have evolved to traffic linear or circular DNA substrates in G+s and some other species. That pELF2-like plasmids are responsible for dissemination of AB resistance among Enterococci makes them medically important subjects for studies aimed at elucidating their unusual transmission mechanism. Overall, the studies are well-developed, with the exception that the authors could not report effects of deleting several tra genes. The findings should attract a broad interest among readership interested in mobile elements, AB resistance transmission, and genome evolution.

Reviewer #2: In their manuscript entitled "A Novel Conjugation System in AMR-associated pELF-type Linear Plasmids of Enterococcus faecium", Kurushima ete al. used a combination of RNA-Seq, molecular constructions, confocal analysis, phylogenetic representations and in silico folding techniques to investigate the conjugative transfer machinery of a van-carrying linear plasmid of E. faecium. They could detect two genes essential for plasmid transfer and delineate the way they interact within the bacterial cell. They finally suggest that they are facing a novel conjugative system and propose a mechanistic working model.

Unfortunately, I think that the extensive work presented here fails to reach the proposed objective, i.e. describing a "novel conjugation system". The authors inferred a transfer operon of their linear plasmid based solely on the distant homology of one protein to coupling proteins of T4SS conjugative machinery and co-expression in RNA-seq experiments. Although they show that three of the 12 genes from the operon are indeed involved in transfer (with two essential), four seems not to be, and no information could be provided for the remaining six. The requirement of other proteins from outside the operon was also not tested. In the present state of the study, the conjugation system of pELF-type plasmids is still far from resolved and may actually not be novel (see below).

In sum, the data presented here are too preliminary to meets the standards required for PLOS Pathogens.

Reviewer #3: (No Response)

**********

**Part II – Major Issues: Key Experiments Required for Acceptance**

Please use this section to detail the key new experiments or modifications of existing experiments that should be absolutely required to validate study conclusions.

Generally, there should be no more than 3 such required experiments or major modifications for a "Major Revision" recommendation. If more than 3 experiments are necessary to validate the study conclusions, then you are encouraged to recommend "Reject".

Reviewer #1: 1. L. 101. Fig. 1. This outcome(s) of the transcriptome analysis was only cursorily discussed – only limited changes in transcriptional profiles were observed and there was some evidence for an operon-like structure to the tra region. Yet, there are several regions/genes that appear to be highly differentially expressed in one or more growth conditions relative to other growth conditions. The authors should consider expanding briefly on some of these findings and perhaps including a suppl table identifying differentially or highly expressed genes and describing any known or predicted functions.

2. Construction and phenotypic analyses of a traG mutation (a probable VirB6 ortholog that is essential for transfer) is likely to expand the number of required subunits of this system to three.

3. Fig. 3 data are uninformative and could either be deleted or moved to a supplemental file.

4. L. 169. Fig. 4. Fig. 4B/C – Why does luciferase activity only begin to increase several hours after initiation of the incubation period and peak at 5-6 h? This seems to be a very slow induction period, especially considering that several rounds of cell division presumptively occur during this time frame.

5. L. 169. Fig. 4. Fig. 4D is not mentioned in the text and should be, but it seems to be showing the transfer kinetics over time – with transfer rates increasing substantially within 60-120 minutes. Accordingly, transfer is occurring well before substantial transcriptional activation of the tra region. Please describe the panel D data in the manuscript and account for the wide difference in kinetics of promoter expression and plasmid transfer.

6. L. 200. And L. 290. Fig. 5. It’s important to establish that the fusion proteins are stably produced, as many such fusions are proteolytically cleaved between the protein of interest and reporter. This could be done by westerns with AB’s against the reporter, or by adding an epitope tag along with the reporter sequence. Also, the apparent dependence of TraD localization on TraC should be strengthened by quantifying the instances of TraD diffuse vs localized patterns in the absence or presence of TraC among a large number of cells. It is mentioned in the discussion that only a limited number of cells clearly exhibit localization, without some type of quantitative comparison, any suggestion that TraD punctate localization patterns depend on TraC production is premature and should be deleted. Which raises a question of whether the data in Fig. 5 should even be presented.

Reviewer #2: The full characterization of all genes required for transfer is necessary. It could be achieved by construction of a minimal non conjugative linear plasmid, with only genes essential for replication and partitioning into which genes are successively added until transfer is restored (starting with genes from the operon of interest), as was done successfully for the Streptomyces linear plasmid SAP1 into which only two genes appeared to be essential (10.1093/bbb/zbaf090).

The authors should also consider functional homologies between the SAP1 conjugative system and pELF.

Additionaly, HMM protein or alphafold structural comparisons of all genes of the operon to known databases could help to infer their putative functions and provide a consistent mechanistic model .

In introduction, current state of the art about conjugative systems in general, and of other linear plasmids, is critically needed. Although studies mainly focused on Streptomyces linear plasmids, some systems have been totally decrypted including the one stated above (10.1093/bbb/zbaf090).

The phylogenetic analysis would require more precision. I don't understand where the 675 E. faecium genomes came from. In RefSeq there is currently more than 5000 genomes available for this speices and probably still more than 2000 two or three years ago. On the other hand there is only 413 complete ones, and not 675. pELF-like plasmids were collected using the 500bp terminal region but the authors ended up with only complete plasmids. I can't believe that no pELF plasmid was present in several contigs in draft genomes.

Reviewer #3: (No Response)

**********

**Part III – Minor Issues: Editorial and Data Presentation Modifications**

Please use this section for editorial suggestions as well as relatively minor modifications of existing data that would enhance clarity.

Reviewer #1: 1. L. 22. Transconjugative/transconjugation – this word is not generally used to refer to conjugation, suggest substituting conjugation for transconjugative or transconjugation throughout.

2. L. 51. Abstract. This overstates the findings; in fact, the authors identified VirB4 and FtsK/VirD4-like subunits and a likely VirB6 ortholog, and demonstrated that the first two are essential. This aligns the pELF2 system with other systems described in species mentioned above that appear to rely on only two or three of the 6 or more components characteristically found among other conjugation systems in G- and G+ species.

3. L. 52. In fact, the authors have not confirmed the absence of the pELF2 genes among other conjugation systems, as this would entail an exhaustive analysis of the entire collection of tra systems. What the authors have shown is that the pELF2 tra genes are widely conserved among other pELF-type plasmids.

4. L. 54. As this system likely resembles those previously identified in a few other G+ species, it is not unique, but rather a new member of this unusual group of conjugation systems whose architectures and mechanisms of action remain unknown.

5. L. 119. The authors should mention that TraC is a likely VirB4-like ATPase. The evidence for this rests on the fact that, like the VirD4-like subunits, it is essential for transfer and is a member of the FtsK-like ATPases; also, it adopts a predicted homohexameric architecture and modeling suggests stacking of TraC and TraD as also predicted for the VirB4/VirD4 arrangement.

6. L. 224. The discussion should highlight similarities in the present findings with the features of conjugation systems identified in a few other G+ systems (Streptomyces, Mycobacteria), Mycoplasma, Thermococcus, and Pseudomonas aeruginosa (PAP1 system). In all of these systems, an Ftsk/VirD4 motor protein functionally interacts with a VirB4-like ATPase and likely one or more integral membrane proteins (possible VirB6 orthologs) to mediate transfer of linear fragments of chromosome or circular plasmids.

7. L. 310. A recent paper described the structure of the TcpA, a T4CP associated with a conjugation system in Clostridium. This structure is more reminiscent of FtsK than VirD4, leading the authors to speculate that pCW3, the plasmid that codes for TcpA, is transferred as a dsDNA substrate rather than being converted to an ssDNA intermediate via RCR. Foor a broader comparison, the authors should consider presenting this structure in the figure along with the predicted structures of TraC/D and the pIP501 homolog.

8. Fig. S1. It should be mentioned in the legend that the three lines represent growth curve replicates for the same strain – presuming that this is what’s being shown.

Reviewer #2: other issues:

Genes of the suspected operon should not be named until their functions are characterized. In fact, the authors showed themselves that traE, traF, traL and traK seems to not be involved in transfer. Naming them tra is therefore highly speculative and misleading. I recommend naming genes of the operon as orfX, with X being ther rank in the operon or from the start of the plasmid sequence.

On the other hand, as TraD shows domain and structural similarities with VirD4 proteins, it would preferably be named VirD4 to match current nomenclature of conjugative system (10.1093/femsre/fuaf069), or at least TraB_D4 as proposed for Streptomyces plasmids. As of traC, it could be named VirB3 according to the two transmembrane proteins and C-terminal binding region functionally similar to other virB3.

traK is obviously less expressed in the stationnary phase (fig 1B). What was the rationale to still include it in the investigated operon?

Numerous English grammar and typo errors.

The purpose of testing the regulation of the operon by any of the genes therein should be indicated. Conjugation is usually regulated by specific regulatory genes not related to the pilus construction, which react to other stimuli such as bacterial stress. Results of the analysis were therefore highly expected and the figure should be put in suppl. information.

Fig. 6: There is some inconsistency for some genomes between panel B and panel C. For instance, NZ_CP045014 have all conserved genes but show only a very small conserved region with the sequence just above also having all conserved genes. The five following genomes also show similar inconsistencies.

AlphaFold analyses deserve to be moved into the results section and have a Method section.

In the methods sections, the threshold to consider a cluster as conserved is not given, as well as parameters for the tree construction (ML tree, NJ tree, bootstraps?). Bootstrap values for the reconstructed tree should also be given.

Minor comments:

The authors consistently use the term "transconjugation" instead of "conjugation". Although the term may be acceptable (but not recommended in my opinion) when it refers to lab-controlled experiments, it should ne be used to describe the general process of conjugation (e.g. l.98 and later).

l 85. Why interestingly ? Please remove;

l.96 [...] or even the linear [...] grammatically incorrect, can't understand the meaning of the sentence.

Fig1 -> B and C panels are inverted. Please provide the reference gene(s) for the gene expression matrix. What does TPM means?

Fig 1C -> typos in traF and traL

Table 1: typos in footnotes and headers. Provide Accession numbers in footnotes 1.

l. 124: upstream and not downstream.

l. 131: essentiality of what?

l.141-142: transfer efficiency values do not match what shown in figure.

l.150: results for with and without empty vector are not shown in figure. Please add them.

l.162: add the control KUHS13 control in figure 3.

l.217: traF is not much conserved compared to some other genes of the operon. Why give this one as example?

l.221: 4 genomes lack the trapELF operon, not 3.

l.286-287. We don't known wether amino-acid ranges refer to positions in the protein or a region size. PLease calrify.

l.325. "[..] one of the most common plasmid families" -> add "in E. faecium".

Please re-organize the method section to Match the order of the results.

Please provide the primers used to confirm your constructions by sequencing.

Reviewer #3: (No Response)

**********

PLOS authors have the option to publish the peer review history of their article (what does this mean?). If published, this will include your full peer review and any attached files.

If you choose “no”, your identity will remain anonymous but your review may still be made public.

**Do you want your identity to be public for this peer review?**  For information about this choice, including consent withdrawal, please see our Privacy Policy.

Reviewer #1: No

Reviewer #2: **Yes:** Sebastien Leclercq

Reviewer #3: No

[NOTE: If reviewer comments were submitted as an attachment file, they will be attached to this email and accessible via the submission site. Please log into your account, locate the manuscript record, and check for the action link "View Attachments". If this link does not appear, there are no attachment files.]

**Figure resubmission:**

**Reproducibility:**

?>

Attachment Submitted filename: reviewing_PPATHOGENS-D-26-00224.pdf

# Author comment: Author response to Decision Letter 1

**DOI:** 10.1371/journal.ppat.1013937.r002

6 May 2026

Attachment Submitted filename: Responces_to_comments.docx

# Peer review: Decision Letter 1

**DOI:** 10.1371/journal.ppat.1013937.r003

**Authors:** Michael Otto, Breck Duerkop

15 Jun 2026

Identification of the essential conjugation machinery in antimicrobial resistance-associated pELF-type linear plasmids of opportunistic pathogen Enterococcus faecium

PLOS Pathogens

Dear Dr. Kurushima,

Thank you for submitting your manuscript to PLOS Pathogens. After careful consideration, we feel that it has merit but does not fully meet PLOS Pathogens's publication criteria as it currently stands. Therefore, we invite you to submit a revised version of the manuscript that addresses the points raised during the review process.

Please submit your revised manuscript by Aug 14 2026 11:59PM. If you will need more time than this to complete your revisions, please reply to this message or contact the journal office at plospathogens@plos.org. When you're ready to submit your revision, log on to https://www.editorialmanager.com/ppathogens/ and select the 'Submissions Needing Revision' folder to locate your manuscript file.

Please include the following items when submitting your revised manuscript:

* A letter that responds to each point raised by the editor and reviewer(s). You should upload this letter as a separate file labeled 'Response to Reviewers'. This file does not need to include responses to any formatting updates and technical items listed in the 'Journal Requirements' section below.

* A marked-up copy of your manuscript that highlights changes made to the original version. You should upload this as a separate file labeled 'Revised Manuscript with Track Changes'.

* An unmarked version of your revised paper without tracked changes. You should upload this as a separate file labeled 'Manuscript'.

We look forward to receiving your revised manuscript.

Kind regards,

Breck A. Duerkop

Academic Editor

PLOS Pathogens

Michael Otto

Section Editor

Editor-in-Chief

PLOS Pathogens

orcid.org/0000-0003-2946-9497

Editor-in-Chief

PLOS Pathogens

orcid.org/0000-0002-7699-2064

**Additional Editor Comments (if provided):**

Thank you for your patience while we reassessed your manuscript "Identification of the essential conjugation machinery in antimicrobial resistance-associated pELF-type linear plasmids of opportunistic pathogen Enterococcus faecium" at PLOS Pathogens. You'll see that although the reviewers are favorable of your revisions, there remain some underlying issues that need to be addressed. Reviewer's 1 and 3 are mostly satisfied with only minor remaining comments. Reviewer 2 has a few more critical issues to address before we can proceed. Please pay careful attention to these and the other points raised by the other reviewers. We will assess your revised manuscript and response to these reviewer comments, aiming to avoid further rounds of peer review if possible.

**Journal Requirements:**

If the reviewer comments include a recommendation to cite specific previously published works, please review and evaluate these publications to determine whether they are relevant and should be cited. There is no requirement to cite these works unless the editor has indicated otherwise.

1) Your Response to Reviewers should be uploaded as a single, individual file. Please remove your Response to Reviewers from your Cover Letter.

2) You have answered NO to the question "Do the authors confirm that all data underlying the findings described in their manuscript are fully available without restriction?". However, you have not indicated a reason for this policy exemption in your Data Availability Statement. Please kindly revise your statement to explain your reasoning, and we will seek the editor's input on an exemption. Please be assured that, once you have provided your new statement, the assessment of your exemption will not hold up the peer review process.

3) PLOS now requires that authors provide the original uncropped and unadjusted images underlying all blot or gel results reported in a submission’s figures or Supporting Information files. This policy and the journal’s other requirements for blot/gel reporting and figure preparation are described in detail at https://journals.plos.org/plosone/s/figures#loc-blot-and-gel-reporting-requirements and https://journals.plos.org/plosone/s/figures#loc-preparing-figures-from-image-files.

When you submit your revised manuscript, please ensure that your figures adhere fully to these guidelines and provide the original underlying images for all blot or gel data reported in your submission. See the following link for instructions on providing the original image data: https://journals.plos.org/plosone/s/figures#loc-original-images-for-blots-and-gels.

In your cover letter, please note whether your blot/gel image data are in Supporting Information or posted at a public data repository, provide the repository URL if relevant, and provide specific details as to which raw blot/gel images, if any, are not available. Email us at customercare@plos.org if you have any questions.

4) Your current Financial Disclosure states, “This work was supported by grants from the Japanese Ministry of Health, Labor and Welfare (Research Program on ensuring Food Safety, 21KA1004 and 24KA1005 to HT), the Japan Society for the Promotion of Science (JSPS) (22K07067,26K02051 to HT, 22K07052, 26K10014 to JK and 26K18620 to NO), the Naito Foundation to JK, Ohyama Health Foundation Inc. to JK, GSK Japan research grant to JK, Takeda Science Foundation to JK and Astellas Foundation for Research on Metabolic Disorders to JK. Research grant form The Association for the Advancement of Science and Technology, Gunma University, to NO. The funders had no role in study design, data collection and analysis, decision to publish, or preparation of the manuscript.” However, in your funding information on the submission form, "order of grants" mismatches. Please indicate by return email the full and correct funding information for your study and confirm the order in which funding contributions should appear. Please be sure to indicate whether the funders played any role in the study design, data collection and analysis, decision to publish, or preparation of the manuscript.

**Reviewers' Comments:**

Reviewer's Responses to Questions

**Part I - Summary**

Please use this section to discuss strengths/weaknesses of study, novelty/significance, general execution and scholarship.

Reviewer #1: The authors have presented a considerably strengthened revision of their previous submission, notably by carefully addressing the reviewers’ comments. They succinctly delineate the major modifications and improvements at the beginning of their rebuttal. The demonstration that a VirB6-like protein is required for transfer supports a more general conclusion that this system is representative of a small but growing family of T4SSs that rely on only a few of the components characteristically required for DNA transfer in Gram-negative species. Overall, the revised manuscript is well-written, and the findings significantly advance our understanding of how a large family of pELF plasmids disseminates among enterococci. The authors draw interesting comparisons with other Gram-positive systems, further enhancing the broad appeal of the work. The one major concern is that the fluorescence microscopy studies exploring the spatial localization of TraC and TraD do not contribute anything substantive to the manuscript, and I suggest deleting these analyses. Other than that, I have only relatively minor comments.

Reviewer #2: In this revision, Kurushima et al. did great improvement on their manuscript, and even identified a third essential gene. Most comments were properly answered, and the bioinformatics analysis was carried out on the all available E. faecium genomes, which provide more accurate results.

I still have some few critical and minor comments, listed below.

Reviewer #3: The authors have adequately addressed my previous comments and revised the manuscript accordingly.

I only have a few minor points that should be corrected before the manuscript can be accepted for publication.

**********

**Part II – Major Issues: Key Experiments Required for Acceptance**

Please use this section to detail the key new experiments or modifications of existing experiments that should be absolutely required to validate study conclusions.

Generally, there should be no more than 3 such required experiments or major modifications for a "Major Revision" recommendation. If more than 3 experiments are necessary to validate the study conclusions, then you are encouraged to recommend "Reject".

Reviewer #1: None

Reviewer #2: Title still inaccurate : still no idea if the whole essential machinery is identified, since several genes could not be deleted and that the conjugative machinery may also require genes from outside the operon.

l.122 and abstract: I don't agree with the statement that the transcriptomic analysis identified the conjugation operon, since no expression change was shown with/without recipient. In this revised manuscript, I feel that the whole transcriptomic analysis is not relevant for the question, and should be reduced to the operon boundaries part. The result section should start l.142.

Gene numbering is a bit awkward. the authors explain that orf12 was not annotated in the reference sequence AP022343.1 of pELF2 so they had to name this gene afterward (ll.152-154). But when looking at the Genbank entry for AP022343.1 (last modification 2020), the ORF is actually annotated under the locus tag EfmKUHS13_31160! It just doesn't start at the exact same position. So this part should be corrected, and gene names should be ordered accordingly. Similarly, locus tags in Table 1/main text/figures/tables should match those of the AP022343.1 annotation.

I still consider that since a unified nomenclature was proposed for conjugation systems (Christie et al. 2025 DOI: 10.1093/femsre/fuaf069), one should follow it as much as possible. Which means that genes should be named traD_<d4> (=subscript), traC_<b4>, and traG_<b6>. The operon doesn't have to be called trapELF (i.e. discussion), tra is enough, unless when compared to other conjugation systems.

Figure 3B and related text: Why cloning the reporter vector only in the strain harbouring pELF2? I think this experiment requires an additional test with the reporter vectors cloned in one of the strains lackin pELF2. Such experiment should indicate if the tra operon expression requires regulatory genes located on the plasmid itself or not.

Figure 5 show an unexpected prevalence of traG in numerous genomes lacking pELF2. It suggests that the detection threshold is too low, or that this same protein is also present in another MGE (through genetic exchange). Please provide some genetic explanation to this observation.

-l.177-180: Deletion of orf8 indeed seems to have an effect in filter mating but not in broth, and for orf9 it is not that clear compared to the other genes. Require statistical analyses here between mutants and WT (with correction for multiple tests), or shorten this part to say that there is only marginal effects for the other genes.

-l.194-196: same remark about orf8 and orf9.</b6></b4></d4>

Reviewer #3: (No Response)

**********

**Part III – Minor Issues: Editorial and Data Presentation Modifications**

Please use this section for editorial suggestions as well as relatively minor modifications of existing data that would enhance clarity.

Reviewer #1: 1. Fig. 1C. Why are there two orf12’s? Are these identical genes, or is the second mislabeled (should it be orf11, since that is mentioned in the text but is not labeled in the figure.

2. Table 1 is not configured properly in my pdf file, but I suspect it’s ok in the submitted manuscript.

3. Pg. 14. L. 177. Differences in transfer frequencies by the orf8 and orf9 (also orf6 and orf12) deletion mutant relative to the WT strain should be evaluated using statistical analyses. If the differences are statistically significant, it’s ok to mention that slight reductions were observed. If not statistically significant, it should be mentioned that any observed slight reductions were not statistically significant.

4. Pg. 9. L. 141. Fig. S1B. Should this be Fig. S2B? If so, there needs to be an A and a B added to the left and right transcriptional profiles, respectively, in Fig. S2.

5. Pg. 15. L. 194. These slight reductions (more obvious for the orf8 deletion, not at all evident for the orf9 deletion) and the putative enhancement of transfer upon complementation should be evaluated for statistical significance.

6. P. 15. L. 201. Delete all references to reductions in transfer by the orf8 and orf9 deletions unless shown to be statistically significant.

7. P. 17. L. 252. Fig 4C. The figure shows that each TraC monomer has 2 possible TM helices, not multiple. The hexamer is predicted to have 12. It’d be more accurate to state either that the monomer has two predicted TM’s or the hexamer has 12 predicted TM’s.

8. P. 18. L. 257. This line of investigation doesn’t provide any evidence supporting the proposed interaction, which could be due to the transient nature of the interaction as the authors suggest. However, the diffuse membrane distribution of TraC is most likely an artefact resulting from overproduction of the reporter-tagged TraC – in all other characterized T4SSs, the T4SSs assemble as discrete machines at the cell envelope, they are not uniformly distributed around the cell. Assuming that the distribution pattern of TraC is an overproduction artefact, or that only a very small fraction of TraC actually assembles into an active machine, it’s premature to suggest that TraD associates only transiently with TraC. Indeed, the tagged TraC does seem to form regions of brighter fluorescence at certain locations, relative to the dim but uniform membrane fluorescence. Regardless, given that this line of investigation generates no firm conclusions one way or the other, I suggest deleting it. Clearly, further work, e.g., biochemical crosslinking/affinity chromatography, is needed to test the proposed TraD/TraC docking model, which is beyond the scope of this manuscript.

9. Pg. 23. L. 370. I suggest that the authors run an alphafold prediction model with 5 copies of TraG. This clearly shows a transmembrane channel, which potentially could also be docked onto the TraC/D homohexameric assembly. In other T4SSs, the VirB6 homologs invariably assemble as homopentamers that associate with the VirB4 hexamer-of-dimers at the membrane. Whether the authors want to include this additional information in the manuscript is up to them, but the TraG pentameric model certainly supports a proposal that a TraC/D/G ternary complex constitutes the bulk - and perhaps the entirety – of the transfer channel in this species.

10. Pg. 24. L. 380. Based on the figure (S12), the text seems to be overstating the conserved nature of these systems, e.g., all systems are assembled from homologs or orthologs of VirD4, VirB4, and VirB6-like subunits. While most appear to have these homologs, some seem to lack one or both VirB4 or VirB6-like subunits, e.g., Streptomyces systems that only have VirD4-like TraB. The only universally conserved subunit is the FtsK/D4-like ATPase.

Reviewer #2: Abstract:

-l.31: correct "comprehensive" by "extensive", since not all tra genes were mutated.

-l.36: remove "of the protein fusions" and add "for the encoded proteins" at the end of sentence

-l.42-43: assertion too imprecise. transmission of linear plasmids is a very specific case, and do not help understanding evolution and disemination of AMR in general.

Author summary:

-l.52: "several" essential genes.

-l.54-55: Same comment, do not agree since not all mutants were provided. Perhaps the last 3 genes are necessary too.

-l.56: 9,000 "E. faecium" genomes

-l.59: role of "these" unique

Introduction:

-l.105: recently discovered "in E. faecium"

-l.106: the plasmid by itself doesn't allow the integration of MGEs. Please rephrase.

-l.117: "the genetic functions" is unclear. Please rephrase.

-l.118: remove "novel conjugation".

Results:

-l.132: with "a" toxin-antitoxin system. The plasmid my carry more than one. But should not be present after correction.

-Table 1: barely readable with the current formatting.

-l.163: were "tentatively" constructed for every gene

-l.169: "could not be generated"

-l.170-173: for clarification I would suggest to rephrase these two sentences as follows: "After verification that these gene deletions did not affect bacterial growth (Fig S4), the pELF2 deletion mutant collection was evaluated as donor strain in congugative [...]"

-l.196-199: Figure 2B doesn't show transfer efficiencies of the WT KUHS13 with + cloned genes in pMGJK53. So the sentence is not correct.

-l.210-211: replace by "transcriptional start site upstream of the orf1 gene, at position 71,468 in the AP022343.1 reference sequence".

-l.215: please indicate the end position for each cloned promoter region.

-l.262: "exhibited an unstable"

-l.272: remove "evolutionary relationships" since it is not the purpose of the study, and it was not really investigated.

-l.274: E. faecium/lactic complex only.

-l.276: "provided"

-l.280: Phylogenetically speaking, it is not consistent to have both E. faecium and E. lactis mixed in clade B. It is likely caused by strain identification before the formal separation between the two species. When looking at some clade B E. faecium provided in suppl. Table 3, it appears that on their NCBI Assembly page, they are more related to the E. lactis type strain than E. faecium type strain. It should be stated in the text.

-Figure 5: "orf12" and not "tra12"

-l.287: remove "frequently".They always co-occur with most genes of the tra operon.

-l.288: add "(Suppl. Fig S10)" after "the entire dataset"

-l.303-304: don't agree. genes with best prevalence (>93%) are of unknown function in table S4.

- Figure 5b: it is not clear how "core genes" were defined

Discussion:

-l.336: "inferred" instead of "speculated".

-l.346: "in the future"

-l.420: "is close" and not "closer"

-l.438-440: move this part in the first section of the discussion, when discussing about conservation of the tra operon genes. It current position in the middle of molecular construction consideration is not logical.

Methods

-l.526-527 and 529-530: repeated sentence. Romve one.

-.673: Please provide the date at which genomes were retrieved.

-l.686: Please provide similarity/coverage thresholds for blastn hits.

- l.688: 70% amino acid or nucleotide identity?

Figure 1: (A) in legend, provide the accession number of the assembly from which CDS annotation were extracted.

(B) traC is given twice on the figure

(C) orf12 is given twice.

Figure 2: (B) in legend, correct "top" and "bottom" panels by "left" and "right"

(C) in legend, correct "left" and "right" panels by "top" and "bottom"

Figure 4: (A) in legend, "top" and "bottom" switched. Both traC and traD contain an ATP-biding domain.

Figure 5: (A) in legend, species names need to be in italic. How orange dots can give the jackknife value? By its size? dots are drawn only for a minimum jackknife value? Please be more precise. The scalebar is not linked to the jackknife value but to the tree branch sizes. ST identification and tra/core genes conservation procedure should be moved to methods.

Please provide similarity+coverage threshold for Blastn hits.

Suppl. Figures:

Figure S4: correct the gene names in the legend

Figure S6: remove "as expected" in the legend (l.1106)

FIgure S7: correct the gene names in the figure, and be more precise: indicate the name of the plasmid carrying the the overexpressed gene. Remove the pELF2 WT labeling from the figure and put it in the legend (l.1112).

Figure S9: (A) and (B) inverted in the legend.

Figure S10: (B) is not defined.

Figure S11: The open or solid red dots are almost invisible on the figure. Please draw them bigger. And be more precise in the legend: "open and solid red dots".

It would have been useful to mark on Figure 5A the strain from which complete pELF2 sequences were extracted. The authors set up a minimal thresold to aa 70% similarity for protein detection, but we have no idea of the actual diversity in pELF2 plasmids. A blue gradient depicting the similarity compared to the each gene from KUHS13 pELF2, instead of just presence/absence, could provide this evolutionary/epiemiological information.

Figure S12: l.1170 replace "plasmid" by "MGE".

Table S5 and S6: missing references for strains and plasmids.

Reviewer #3: Figure 2A. The authors state that deletion of orf9 resulted in a slight reduction in transfer efficiency. However, according to Figure 2A, the deletion of orf6 or orf12 appears to have a similar effect on transfer efficiency as the deletion of orf9. Did the authors perform any statistical analyses to support the conclusion that these differences are significant? This comment also applies to the other graphs presented in the manuscript.

Consequently, the interpretation of the deletion and complementation effects in Figure 2B should be moderated (lines 194-195), as the observed effect does not appear to be substantially different from that observed for the orf6 and orf12 deletion mutants. The same consideration applies to all statements throughout the manuscript that specifically emphasize an effect of orf9 (e.g., lines 201 and 319-320).

Figure 1C. The last gene of the tra operon should be labeled orf11 rather than orf12. Otherwise, two genes are designated as orf12 in the figure.

Line 189. The sentence “phenotype in the ∆orf3 and ∆orf4 strains” should also include the orf7 mutant. I suggest replacing it with: “phenotype in the ∆orf3, ∆orf4, and ∆orf7 strains”.

**********

PLOS authors have the option to publish the peer review history of their article (what does this mean?). If published, this will include your full peer review and any attached files.

If you choose “no”, your identity will remain anonymous but your review may still be made public.

**Do you want your identity to be public for this peer review?**  For information about this choice, including consent withdrawal, please see our Privacy Policy.

Reviewer #1: No

Reviewer #2: **Yes:** Sebastien Leclercq

Reviewer #3: No

[NOTE: If reviewer comments were submitted as an attachment file, they will be attached to this email and accessible via the submission site. Please log into your account, locate the manuscript record, and check for the action link "View Attachments". If this link does not appear, there are no attachment files.]

**Figure resubmission:**

**Reproducibility:**

?>

Attachment Submitted filename: R1_reviewing_PPATHOGENS-D-26-00224.pdf

# Author comment: Author response to Decision Letter 2

**DOI:** 10.1371/journal.ppat.1013937.r004

30 Jul 2026

Attachment Submitted filename: Responses_to_comments_r2_v4.docx

# Editor report: Decision Letter 2

**DOI:** 10.1371/journal.ppat.1013937.r005

**Authors:** Michael Otto, Breck Duerkop

2 Aug 2026

Dear Dr. Kurushima,

We are pleased to inform you that your manuscript 'Identification of essential genes for conjugative transfer in antimicrobial resistance-associated pELF-type linear plasmids of opportunistic pathogen Enterococcus faecium' has been provisionally accepted for publication in PLOS Pathogens.

Before your manuscript can be formally accepted you will need to complete some formatting changes, which you will receive in a follow up email. A member of our team will be in touch with a set of requests.

Please note that your manuscript will not be scheduled for publication until you have made the required changes, so a swift response is appreciated.

IMPORTANT: The editorial review process is now complete. PLOS will only permit corrections to spelling, formatting or significant scientific errors from this point onwards. Requests for major changes, or any which affect the scientific understanding of your work, will cause delays to the publication date of your manuscript.

Should you, your institution's press office or the journal office choose to press release your paper, you will automatically be opted out of early publication. We ask that you notify us now if you or your institution is planning to press release the article. All press must be co-ordinated with PLOS.

Thank you again for supporting Open Access publishing; we are looking forward to publishing your work in PLOS Pathogens.

Best regards,

Breck A. Duerkop

Academic Editor

PLOS Pathogens

Michael Otto

Section Editor

PLOS Pathogens

Sumita Bhaduri-McIntosh

Editor-in-Chief

PLOS Pathogens

orcid.org/0000-0003-2946-9497

Michael Malim

Editor-in-Chief

PLOS Pathogens

orcid.org/0000-0002-7699-2064

***********************************************************

Reviewer Comments (if any, and for reference):

# Editor report: Acceptance letter

**DOI:** 10.1371/journal.ppat.1013937.r006

**Authors:** Michael Otto, Breck Duerkop

Dear Dr. Kurushima,

We are delighted to inform you that your manuscript, "Identification of essential genes for conjugative transfer in antimicrobial resistance-associated pELF-type linear plasmids of opportunistic pathogen Enterococcus faecium," has been formally accepted for publication in PLOS Pathogens.

We have now passed your article onto the PLOS Production Department who will complete the rest of the pre-publication process. All authors will receive a confirmation email upon publication.

The corresponding author will soon be receiving a typeset proof for review, to ensure errors have not been introduced during production. Please review the PDF proof of your manuscript carefully, as this is the last chance to correct any scientific or type-setting errors. Please note that major changes, or those which affect the scientific understanding of the work, will likely cause delays to the publication date of your manuscript. Note: Proofs for Front Matter articles (Pearls, Reviews, Opinions, etc...) are generated on a different schedule and may not be made available as quickly.

Soon after your final files are uploaded, the early version of your manuscript, if you opted to have an early version of your article, will be published online. The date of the early version will be your article's publication date. The final article will be published to the same URL, and all versions of the paper will be accessible to readers.

For Research Articles, you will receive an invoice from PLOS for your publication fee after your manuscript has reached the completed accept phase. If you receive an email requesting payment before acceptance or for any other service, this may be a phishing scheme. Learn how to identify phishing emails and protect your accounts at https://explore.plos.org/phishing.

Thank you again for supporting open-access publishing; we are looking forward to publishing your work in PLOS Pathogens.

Best regards,

Sumita Bhaduri-McIntosh

Editor-in-Chief

PLOS Pathogens

orcid.org/0000-0003-2946-9497

Michael Malim

Editor-in-Chief

PLOS Pathogens

orcid.org/0000-0002-7699-2064
